# An AI system to help scientists write expert-level empirical software

**DOI:** 10.1038/s41586-026-10658-6

**Published:** 2026-05-19

**Authors:** Eser Aygün, Anastasiya Belyaeva, Gheorghe Comanici, Marc Coram, Hao Cui, Jake Garrison, Renee Johnston, Anton Kast, Cory Y. McLean, Peter Norgaard, Zahra Shamsi, David Smalling, James Thompson, Subhashini Venugopalan, Brian P. Williams, Chujun He, Sarah Martinson, Martyna Plomecka, Lai Wei, Yuchen Zhou, Qian-Ze Zhu, Matthew Abraham, Erica Brand, Anna Bulanova, Jeffrey A. Cardille, Chris Co, Scott Ellsworth, Grace Joseph, Malcolm Kane, Ryan Krueger, Johan Kartiwa, Dan Liebling, Jan-Matthis Lueckmann, Paul Raccuglia, Xuefei Julie Wang, Katherine Chou, James Manyika, Yossi Matias, John C. Platt, Lizzie Dorfman, Shibl Mourad, Michael P. Brenner

**Affiliations:** 1 Google DeepMind, Montréal, Quebec Canada; 2 Google Research, Cambridge, MA USA; 3 Google Platforms and Devices, Mountain View, CA USA; 4 Massachusetts Institute of Technology, Cambridge, MA USA; 5 School of Engineering and Applied Sciences, Harvard University, Cambridge, MA USA; 6 Google DeepMind, New York, NY USA; 7 Faculty of Agricultural and Environmental Sciences, McGill University, Montréal, Quebec Canada; 8 California Institute of Technology, Pasadena, CA USA

**Keywords:** Computational science, Computer science

## Abstract

The cycle of scientific discovery is frequently bottlenecked by the slow, manual creation of software to support computational experiments^1^. To address this, we present Empirical Research Assistance (ERA), an artificial intelligence (AI) system that creates expert-level scientific software whose goal is to maximize a quality metric. The system uses a large language model (LLM) and tree search^2^ to systematically improve the quality metric and intelligently navigate the large space of possible solutions. ERA achieves expert-level results when it explores and integrates complex research ideas from external sources. The effectiveness of tree search is demonstrated across a diverse range of tasks. In bioinformatics, ERA discovered 40 new methods for single-cell data analysis that outperformed the top human-developed methods on a public leaderboard. In epidemiology, ERA generated 14 models that outperformed the Centers for Disease Control and Prevention (CDC) ensemble and all other individual models for forecasting COVID-19 hospitalizations. ERA also produced expert-level software for geospatial analysis, neural activity prediction in zebrafish and numerical solution of integrals, as well as a new rule-based construction for time-series forecasting. By devising and implementing new solutions to diverse tasks, ERA represents a notable step towards accelerating scientific progress.

## Abstract (web summary)

A new AI system uses a large language model combined with tree and evolution search to quickly create expert-level empirical software and accelerate scientific progress, as demonstrated in a wide range of experimental tasks.

## Main

Empirical software, designed to maximize a measurable quality score, is ubiquitous and central to many scientific endeavours. Empirical software has recently enabled several Nobel Prizes in Chemistry: in 1998 for density functional theory^3,4^, in 2013 for molecular dynamics simulation^5^ and in 2024 for protein structure prediction^6,7^. Empirical software underlies our ability to create models of complex systems, ranging from parameterizations of a vertical column of the Earth’s atmosphere for weather modelling^8^, to the parameterization of stress response in a turbulent fluid flow^9^, to the prediction of social systems^10,11^.

However, empirical software for science is slow and difficult to create. Domain-specific empirical software requires tedious work^1^, often over many years. When empirical software is used to test complex hypotheses, it becomes ever more difficult to write purely from first principles. There is usually no systematic search for alternative approaches. Design choices are often governed by intuition or expediency rather than exhaustive experimentation. Creating the software is so time-consuming that it severely limits the possibilities that can be productively explored^12^.

**Fig. 1:**
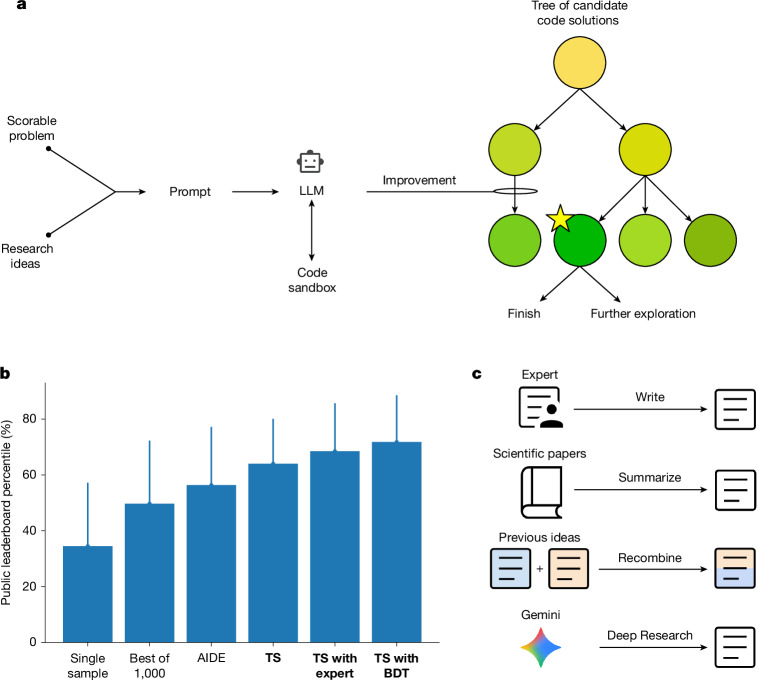
Schematic and performance of ERA. **a**, Schematic of the ERA algorithm. A scorable task, together with research ideas proposing methods to solve the task, are fed to a LLM, which produces code to evaluate the scorable task in a sandbox. This is then embedded within a tree search algorithm, in which new nodes are chosen balancing exploitation and exploration, sampling from the LLM (Methods). **b**, Performance of code generation methods on the Kaggle Playground benchmark. Results report the average public leaderboard percentile performance over 16 tasks. Methods based on ERA are listed in bold. BDT, boosted decision tree; TS, ERA-based tree search. Error bars indicate the standard deviation of performance between different competitions in the benchmark. **c**, The system automates empirical software development by iteratively optimizing predictive models and computational algorithms based on research ideas and a defined quality metric. We use LLM summaries of scientific papers or AI-assisted literature search as part of the prompt and also recombine successful implementations of ideas to create more powerful methods.

Here we present ERA, an AI-based system that systematically and automatically creates empirical software to solve scorable tasks. ERA is based on a LLM that rewrites software to attempt to improve its quality score. ERA creates several software candidate solutions and uses tree search^2^ to decide which candidates merit further exploration (Fig. 1a). Although there are many ways of designing a code mutation system^13–16^, we developed ERA by competing in Kaggle competitions (Fig. 1b), described below. We augment code mutation with research ideas obtained from a range of sources, including highly cited papers, specialized textbooks and search engine results (Fig. 1c). In practice, these ideas can be injected either directly by the user or automatically using a search engine to access research in the literature. The LLM uses this injected guidance in writing code.

We find that ERA produces software that outperforms the state of the art in scorable tasks spanning broad scientific disciplines. This expert-level performance arises because of the ability to exhaustively and tirelessly carry out solution searches at unprecedented scale, identifying needle-in-the-haystack, high-quality solutions.

## Overview of scorable tasks

We develop ERA on Kaggle Playground competitions and test it by selecting scorable tasks based on scientific or engineering problems of high relevance in diverse fields. These scorable tasks are listed below, with per-node computational costs outlined in Supplementary Table 1.

Single-cell RNA sequencing (scRNA-seq) batch integration^17^: this task requires distinguishing subtle biological signals from noise in high-dimensional sparse datasets. By removing confounding factors, we can enable large-scale multi-lab transcriptomic data integration.

CDC COVID forecasting^18^: this task requires predicting nonlinear disease dynamics from lagged and noisy real-time data. By predicting COVID cases several weeks in advance, we can inform public health policy and resource allocations.

Time-series forecasting^19^: this task requires predicting time-series outcomes across a range of datasets and applications.

Geospatial segmentation^20^: this task requires performing dense pixelwise multi-label semantic segmentation on complex satellite imagery. Better segmentation can lead to large improvements in environmental monitoring and disaster response.

ZAPBench^21^: this task requires predicting the activity of more than 70,000 neurons across an entire vertebrate brain. Performing well on this benchmark may lead to a systems-level understanding of brain function and behaviour.

Numerically solving difficult integrals: this task requires solving integrals that defy standard numerical algorithms. It is useful for modelling physical and engineering systems.

## Kaggle Playground benchmark

We designed ERA to score highly on a curated set of Kaggle competitions. Kaggle calibrates human performance with percentile rank on a leaderboard and we score code by submitting directly to Kaggle. Our benchmark consists of 16 Playground competitions from the 2023 season, encompassing regression and classification tasks (Supplementary Table 2). Playground competitions are an ideal benchmark because they offer fast iteration, simplicity and calibration against thousands of humans. Achieving a high score requires creating complex code without requiring solving a sophisticated scientific task.

Our basic strategy uses a simple prompting template (Supplementary Table 3) that concatenates the competition description with the previous trial. Figure 1b evaluates the performance of ERA with the average public percentile rank across all 16 Playground competitions: ERA substantially beats a single LLM call and best-of-1,000 LLM calls and also outperforms AIDE^13^, owing to its ability to maintain a diverse tree of candidates, allowing it to backtrack when a specific line of code mutation plateaus. During the search, the system discovers strategies leading to abrupt jumps in the score, with the accumulation of these jumps leading to the highest-quality solutions.

Problem-specific advice added to the prompt substantially improves performance. We illustrate this with two examples. In tree search with expert advice, we give ERA standard advice to win Kaggle competitions (Supplementary Table 4). In tree search with boosted decision tree, we tell ERA to implement a boosted decision tree library from scratch, without using standard packages (Supplementary Table 5). We manually verified in both cases that resulting codes followed the advice.

We now evaluate ERA on six benchmarks in different scientific fields, exploring distinct ways to incorporate research ideas to improve system performance (Fig. 1c and Methods).

## Genomics: batch integration of scRNA-seq data

scRNA-seq has revolutionized our ability to dissect cellular heterogeneity, discover new cell types, infer gene regulatory networks and developmental trajectories and improve therapeutic target prioritization^22^, enabling hundreds of millions of cells to be individually sequenced within thousands of datasets^23–25^. A notable challenge required to jointly analyse many disparate scRNA-seq datasets is to computationally remove complex batch effects present across samples while preserving biological signals^26^. Nearly 300 tools exist to perform batch integration of scRNA-seq data^27^ and several benchmarks have been developed for assessing metrics of batch effect removal and conservation of biological variability^28–30^.

**Fig. 2:**
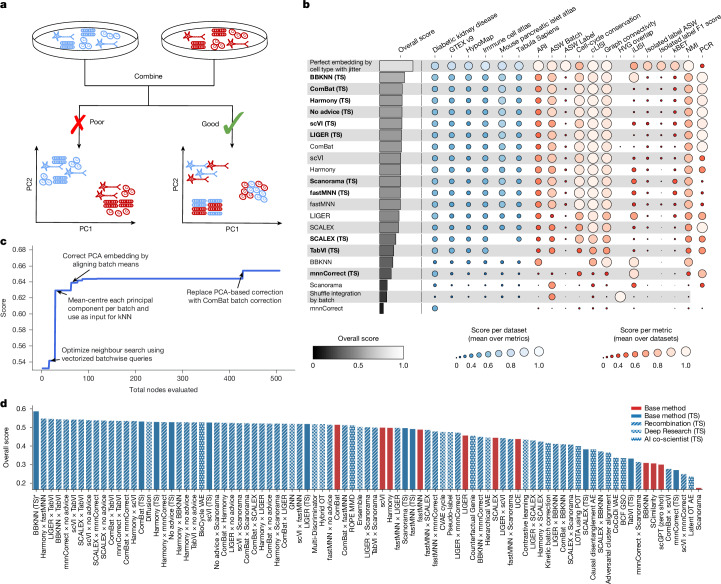
Performance of ERA on scRNA-seq batch integration. **a**, Schematic of the batch integration task, in which disparate datasets (blue and red) are processed to remove batch effects in the data while retaining biological variability. **b**, Performance of tree search (method names bolded and suffixed by ‘(TS)’) compared with the analogous published method on the OpenProblems v2.0.0 benchmark^30^. ‘Perfect embedding by cell type with jitter’ is a positive control method that represents the best possible performance and ‘Shuffle integration by batch’ is a negative control that does not perform any batch integration. Overall score is the mean over all datasets and metrics. Each ‘Datasets’ column shows the mean of all metrics computed over that dataset. Each ‘Metrics’ column shows the mean of that metric computed over all datasets. Metrics were assigned a value of 0 if they could not be computed or if their performance was worse than the lowest negative control; these are shown as empty cells. **c**, Performance improvements annotated with code innovation for the top-performing BBKNN implementation. ComBat-based embedding generation was introduced in implementation attempt 429. **d**, Overall score for the OpenProblems v2.0.0 benchmark (ref. ^30^) non-control methods, ERA with and without recombination of ideas, Gemini Deep Research^34^ and ERA with AI co-scientist^35^. *y*-axis lower bound is the overall score of the ‘Shuffle integration by batch’ negative control method. Seven recombination methods, five base methods and two AI co-scientist methods that do not match its performance are omitted. *The method is a recombination, even if not explicitly prompted for recombination. fastMNN, batchelor fastMNN; kNN, *k*-nearest neighbours; mnnCorrect, batchelor mnnCorrect; PC, principal component; TS, ERA-based tree search.

To assess ERA performance on this task, we used the OpenProblems v2.0.0 batch integration benchmark^30^. As of July 2025, this active benchmark evaluates 15 state-of-the-art methods and eight control methods on 13 different metrics that quantify both the ability to remove batch effects in the data and retain variability attributable to true biological differences in six datasets spanning human and mouse^25^ (Fig. 2a). To avoid overfitting to the benchmark, we used a separate dataset for ERA optimization (Methods and Supplementary Fig. 1). For each ERA run, we selected the best solution based on the performance on this training set and report the performance on the holdout OpenProblems datasets (*n* = 1,747,937 total cells). We prompt the LLM with a description of the single-cell batch integration problem, code for reading in the dataset, code for evaluation metrics and optional text with a particular research idea.

**Extended Data Fig. 1:**
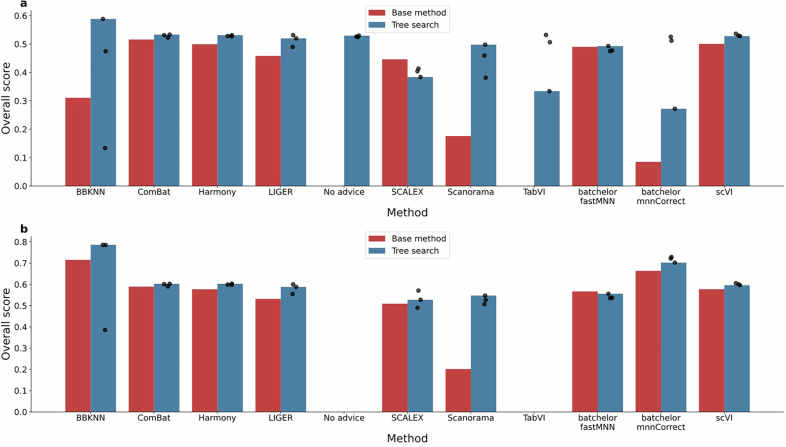
Relative performance of base methods and ERA replicates. **a**, Overall scores on the holdout OpenProblems datasets for all replicates of methods evaluated in Fig. 2. For tree search implementations, three replicates of the full process were performed. Dots indicate the overall score of the replicate on the holdout OpenProblems datasets. The bar shows the performance of the replicate with highest performance in the validation dataset (identical values to those shown in Fig. 2). The lowest-performing tree search replicates for BBKNN, Scanorama and TabVI only successfully computed 30, 57 and 45 of the 78 metrics, respectively. We note that failures owing to out-of-memory or compute time issues were not explicitly selected against in our algorithm, as all optimization was performed on datasets of only 20,000 cells. **b**, Average scores for each method when restricting to only (method, dataset, metric) combinations that have non-NaN values for the base method and all three tree search replicates. No advice and TabVI are absent because they have no base method comparator.

**Extended Data Fig. 2:**
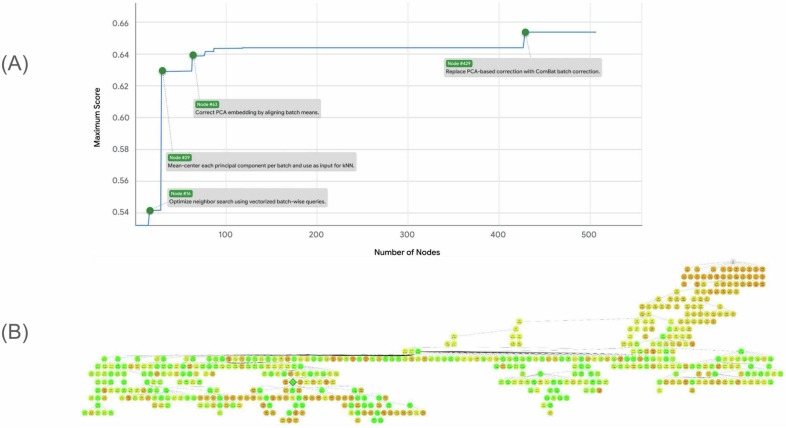
Breakthrough plot and solution tree for the scRNA-seq batch integration task. **a**, Breakthrough plot for the BBKNN (TS) tree search, showing the evolution of the maximum score as a function of the number of nodes. The green dots label places at which the score abruptly increases as a result of an improvement in the code and the label describes the change in the code that resulted in the score increase. **b**, Structure of the tree for this same search. The colour range consists of orange (lower scores) to green (higher scores), with the highest score denoted by a diamond node.

First we ran ERA without guidance and observed that its solution is conceptually similar to ComBat^31^ yet improved over the present OpenProblems leaderboard (‘No advice (TS)’ in Fig. 2b). We then evaluated whether ERA could improve on existing algorithms. We selected nine methods from the OpenProblems benchmark, including the six highest-performing methods (Methods). For each method, we obtained the paper PDF and used Gemini 2.5 Pro to add a brief summary to the prompt (Methods). In pairwise comparisons, ERA outperformed the corresponding published result for eight of the nine methods in overall score (Fig. 2b). The top-performing method was an ERA implementation of batch-balanced *k*-nearest neighbours (BBKNN (TS))^32^, yielding a 14% overall improvement over the best published method (ComBat^31^) and equalled or outperformed the corresponding published BBKNN in every dataset and across 11/13 metrics (Fig. 2b). This performance highlights its capacity to effectively remove batch effects without compromising biological signals (Supplementary Fig. 2). We observed that ERA is also able to produce performant implementations for an algorithm without publicly available code (TabVI (ref. ^33^); Extended Data Fig. 1). Notably, expert manual inspection of the code solutions proposed by ERA confirmed that nearly all implementations adhered to the requested algorithms (Extended Data Table 1), with performance largely consistent across replicate runs of methods (Extended Data Fig. 1). Furthermore, ERA demonstrated improvements even when compared with base methods with optimized hyperparameters, indicating that its contribution extends beyond hyperparameter tuning (Methods and Supplementary Fig. 3). Extended Data Fig. 2 shows the tree structure and evolution of the maximum score as a function of the number of nodes in the tree for the best-performing model, BBKNN (TS).

**Extended Data Fig. 3:**
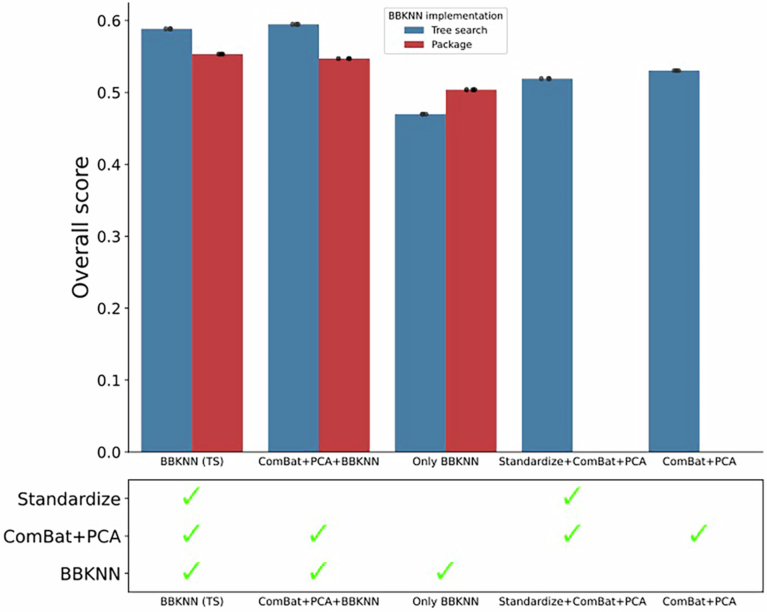
Ablation analysis of the top-performing BBKNN (TS) method. The BBKNN (TS) method performed standard linear expression scaling to 10^4^ total counts followed by log1p transformation. It then applied three more transforms: ‘Standardize’ called sc.pp.scale to further scale the data to mean 0 and unit variance, ‘ComBat+PCA’ called sc.pp.combat followed by sc.tl.pca to generate the expression embedding and ‘BBKNN’ applied an implementation of BBKNN written by ERA. Bars here show the overall performance in the OpenProblems datasets for ablations that include one or more of these components. For each ablation that includes the ‘BBKNN’ component, comparison of the written BBKNN implementation (‘Tree search’) and the bbknn package implementation (‘Package’) is shown. Black dots show the individual performance of three replicates of each method.

For BBKNN (TS), part of the performance boost came from combining two existing methods, ComBat^31^ and BBKNN, rather than simply implementing BBKNN (Fig. 2c). In particular, although the original BBKNN method computes neighbours using principal component analysis (PCA) embeddings, BBKNN (TS) computes neighbours using ComBat-corrected PCA embeddings, removing global linear batch-associated variance. Both implementations then compute *k*-nearest neighbours across batches and construct a graph (with differences in exact implementation), thus removing local batch effects (Supplementary Table 6). Manual modification of BBKNN (TS) and the published BBKNN implementation confirmed that using Combat-corrected PCA embeddings is critical for improving both implementations (Extended Data Fig. 3), confirming the value in idea recombination.

**Extended Data Fig. 4:**
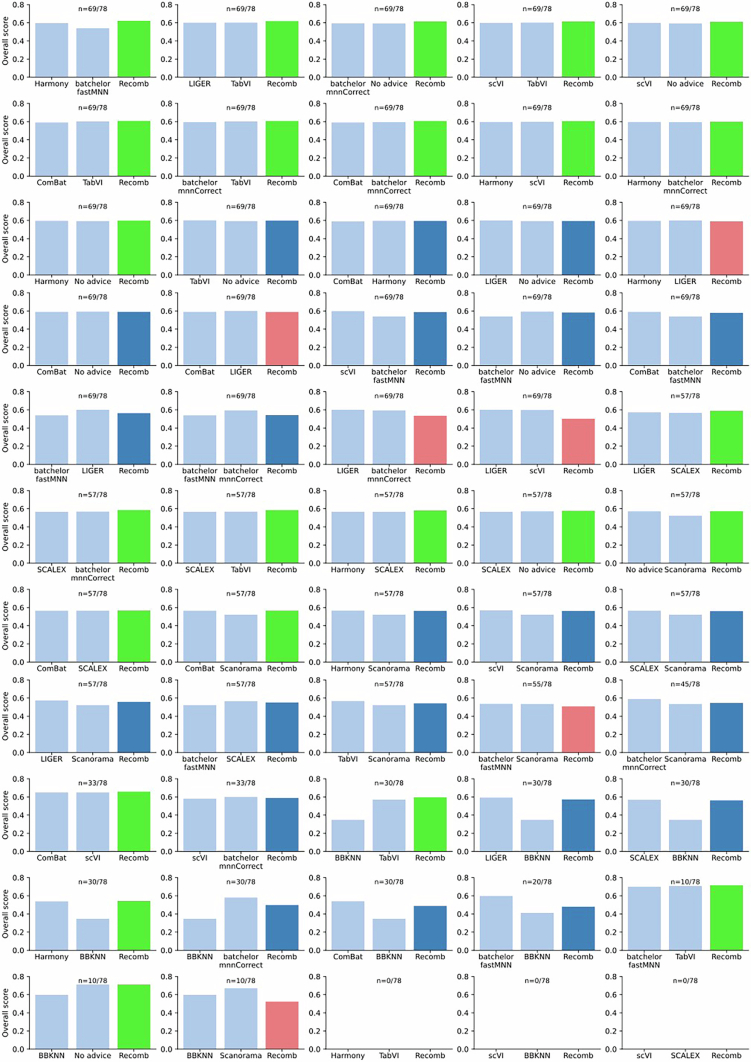
Comparison of tree search performance on base methods and their ‘recombination’ over an intersection of successfully calculated metrics. We ran ‘recombination’ experiments by seeding tree search with the top variants from two base method runs (Methods). We compare the performance of two base methods and ‘recombination’ on the OpenProblems test dataset for all 55 pairwise combinations of the 11 base methods. Because sometimes methods may fail getting a score for certain evaluation metrics owing to errors such as out of memory, we compare the performance on a subset of metrics that were successfully computed for all three methods. ‘*n* = *X*/78’ on each subplot shows the number of successfully computed metrics, *X*, that we averaged over. For each subplot, we show the base methods on the left in light blue and the recombination method on the right (labelled as ‘Recomb’), for which a green bar means that the recombination method outperforms both of its base methods, dark blue means that the recombination method outperforms one of the base methods and red means that the recombination method does not outperform either of the base methods.

This motivated an exploration of systematic ways to generate more complex research ideas. First, similar to how scientists often combine ideas to create a new approach, we programmatically generated 55 ‘recombinations’ of all pairs of the 11 methods described above (no advice, nine replications and TabVI; hereafter, ‘base methods’) based on summaries of the code for each method (Methods and Supplementary Table 7). We ran ERA, prompted with each of these recombinations. For each base method and recombination group, we compared the average scores for the top nodes over the intersection of metrics that were successfully computed for all three methods. Notably, recombination implementations of tree search frequently outperformed their base counterparts, with 24 of the 55 recombination solutions (44%) outperforming both of their base methods and 22 of the remaining 31 recombination solutions outperforming one of the two base methods (Extended Data Fig. 4).

Second we used Gemini Deep Research^34^ and AI co-scientist^35^ to generate and implement 21 more ideas (Methods). After ERA was applied to each, 6/11 base methods, 29/55 recombination, 4/9 Deep Research and 1/12 AI co-scientist methods (40 of 87) outperform all methods published at present on the OpenProblems leaderboard (Fig. 2d).

To further understand the conceptual space explored by ERA, we obtained embeddings for each generated code using Gemini’s text embedding model and computed cosine similarities (Supplementary Fig. 4). Visualization of the embeddings revealed distinct clusters, generally representing deep-learning-based methods and non-deep-learning methods, suggesting that ERA is able to generate a variety of solutions (Supplementary Fig. 5).

## Public health: predicting COVID-19 hospitalizations

The main US benchmark for COVID-19 forecasting is the COVID-19 Forecast Hub (CovidHub, https://github.com/CDCgov/covid19-forecast-hub/tree/main)^18^, a large, collaborative effort coordinated by the CDC. CovidHub receives weekly forecasts from dozens of expert-led teams across academic institutions, industry and government, each using different methodologies. These weekly forecasts must cover new COVID-19-related hospitalizations across 52 US states and territories for the present week and three subsequent weeks over 23 specified quantiles. Submissions are evaluated using the weighted interval score (WIS), which rewards both accuracy and well-calibrated uncertainty.

Top-performing individual models include classic autoregressive time-series approaches (for example, UMASS-ar6_pooled), gradient boosting machine learning models (for example, UMASS-gbqr) and epidemiological models based on renewal equations and Bayesian estimation of the reproductive number (for example, CEPH-Rtrend_covid). CovidHub makes use of this methodological diversity by integrating submissions into the CovidHub-ensemble, a robust aggregate forecast that has historically provided the gold standard for epidemiological prediction in the USA.

**Fig. 3:**
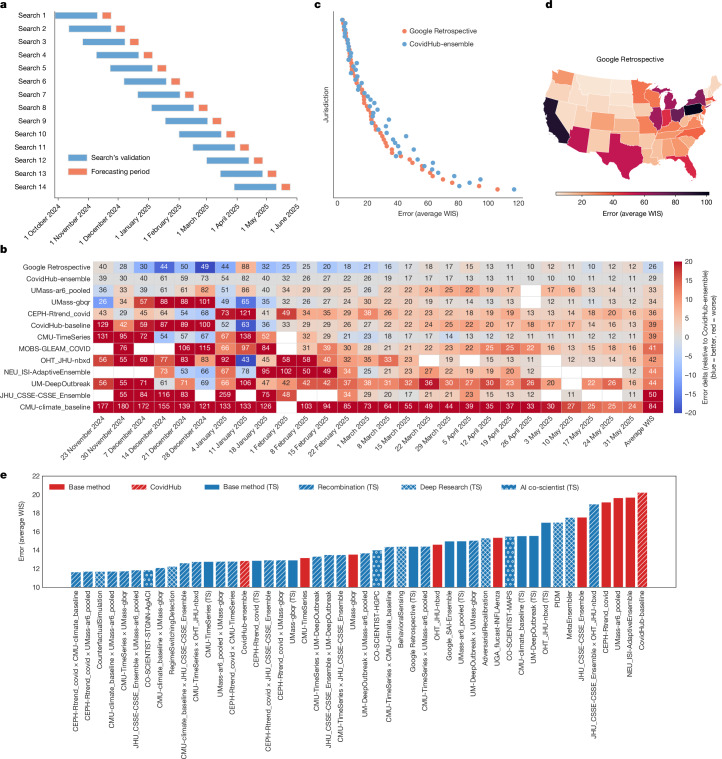
Performance of ERA on COVID-19 forecasting. **a**, Rolling validation window used for the forecasting experiments. The output of each search is validated internally on a preceding block of time (blue) and the resulting model is then used to make predictions for its corresponding forecasting period (orange). Training data include all dates on or after 8 August 2020 and before the validation set. **b**, Time-series leaderboard showing weekly forecasting performance (‘Average WIS’) for participating teams and our ‘Google Retrospective’ model, ordered by average WIS. Scores are aggregated across all 52 jurisdictions and four forecast horizons. The number in each cell is the model’s absolute average WIS for that week. The background colour of the cell visualizes the performance relative to the CovidHub-ensemble, with blue indicating a lower (better) WIS and red indicating a higher (worse) WIS. **c**, Direct jurisdiction-level comparison of forecasting error (average WIS) between our model and the ‘CovidHub-ensemble’, demonstrating the superior performance of our model in most locations. **d**, Geographic distribution of our model’s forecasting error (average WIS), aggregated over the entire 2024/2025 COVID-19 season. Lower error values (lighter colours) indicate better performance. **e**, Comparison of aggregate forecasting performance for various modelling strategies. This includes baseline models from the CovidHub competition, our retrospective model, our replications of submitted models, new hybrid models generated through recombination, Deep Research^34^ and AI co-scientist^35^. Fourteen strategies (ten recombination, two Deep Research, one AI co-scientist and one replicated baseline) outperform the official CovidHub-ensemble for the 3-week (3 reference dates × 4 time horizons × 52 jurisdictions) evaluation period. Models that perform worse than CovidHub-baseline are not shown.

**Extended Data Fig. 5:**
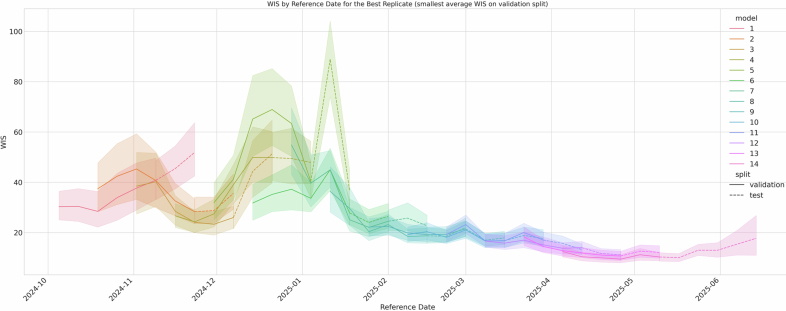
Performance of the best retrospective COVID-19 hospitalization forecast replicates. This figure presents WIS by reference date for the single best-performing replicate of each validation window in our retrospective COVID-19 forecasting study. The best models are selected on the basis of their performance on the validation dates. The plot shows how finding optimum models on a handful of validation dates (6 weeks) generalizes to the next 2 weeks of unseen reference dates.

We designed a rigorous retrospective study to assess ERA performance in this competitive environment, using data available on 1 May 2025. For every forecasting period, we ran ERA to optimize and select a model using data from the preceding 6 weeks, creating a rolling validation window throughout the 2024–2025 season (Fig. 3a), with data splits elaborated in Supplementary Table 8. The weekly performance of our resulting ‘Google Retrospective’ model is detailed in the time-series leaderboard (Fig. 3b), which visualizes the performance advantage of our model relative to the CovidHub-ensemble and other top-performing teams. The temporal variation of the WIS for each of the separate validation splits is shown for the best replicate (Extended Data Fig. 5) and across replicates (Supplementary Fig. 6). A direct jurisdiction-level comparison confirms that our model achieved a lower (better) WIS in most states (Fig. 3c,d). Supplementary Figs. 7 and 8 compare prediction curves for representative locations between different models.

**Extended Data Fig. 6:**
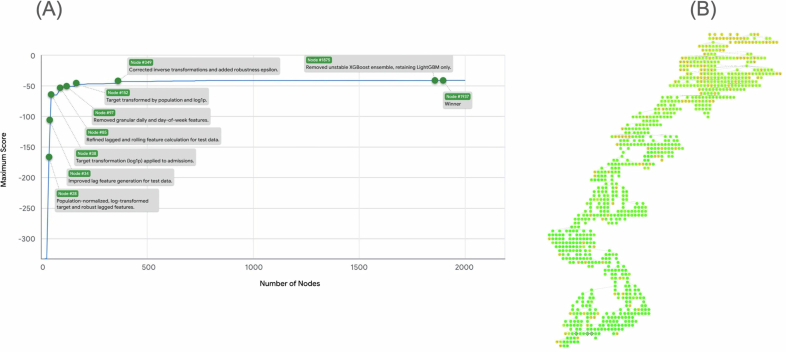
Breakthrough plot and solution tree for the COVID-19 prediction task. **a**, Breakthrough plot for the retrospective COVID-19 prediction, showing the evolution of the maximum score as a function of the number of nodes. The green dots label places at which the score abruptly increases as a result of an improvement in the code and the label describes the change in the code that resulted in the score increase. **b**, Structure of the tree for this same search. The colour range consists of orange (lower scores) to green (higher scores), with the highest scores denoted by diamond nodes.

Overall, our model achieved the highest performance with an average WIS of 26, outperforming the official CovidHub-ensemble’s average WIS of 29. A representative tree and breakthrough plot is shown in Extended Data Fig. 6.

**Extended Data Fig. 7:**
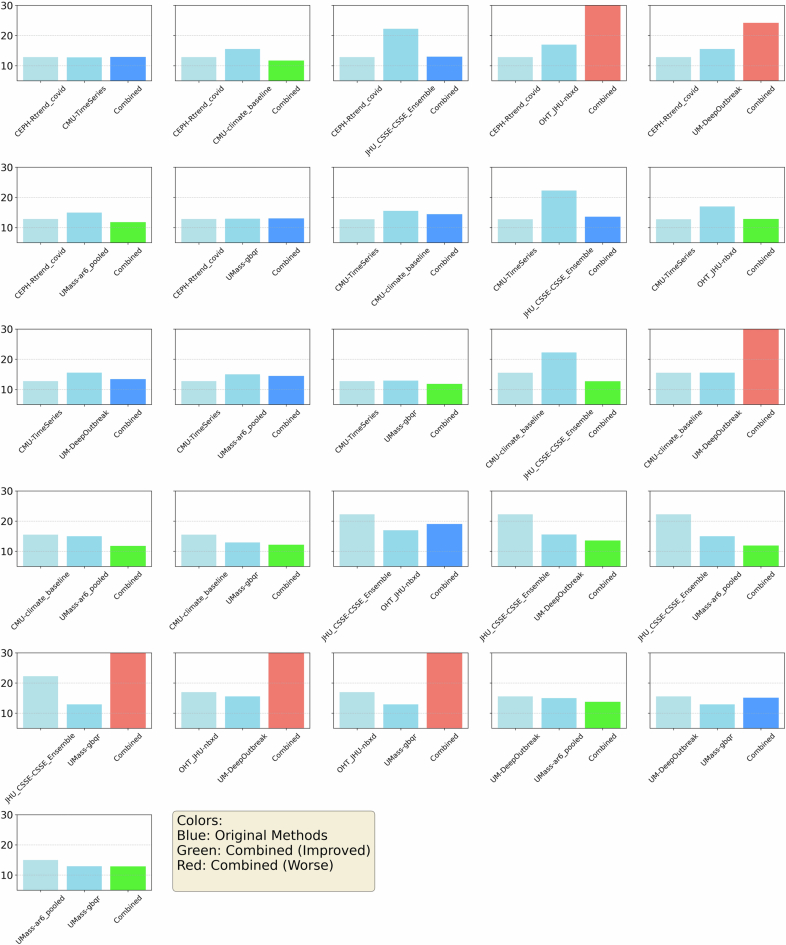
Performance of recombination experiments for COVID-19 forecasting. This series of bar plots illustrates the average WIS achieved by various hybrid models (right bar, labelled ‘Combined’) compared with their constituent baseline models (left bars, typically light blue) from the CovidHub competition. Each subplot represents a recombination experiment, demonstrating the success of our system in synthesizing new forecasting strategies. Green bars indicate that the recombination outperformed both parent models, dark blue indicates that it outperformed one and red indicates that it outperformed neither. These results emphasize the ability of the search system to combine the strengths of existing methodologies to achieve superior predictive performance.

Beyond this retrospective performance, we investigated the ability of ERA to explore the solution space more broadly by replicating, recombining and generating entirely new forecasting strategies (Fig. 3e). First we tested its ability to replicate existing methods from other teams using only their brief public descriptions from the CovidHub (Supplementary Tables 9 and 10). Our tree-search-based implementations (‘Base method (TS)’) not only adhered to the provided instructions (Supplementary Table 11) but also exceeded the performance of the original submissions in six of the eight cases tested; the two models that performed worse (replicating JHU_CSSE-CSSE_Ensemble and OHT_JHU-nbxd) did not use external data present for the original method implementations. Next we explored whether solutions could be improved through recombination. For this experiment, we prompted a LLM to analyse the core principles of two different parent models and then used its synthesis to instruct ERA to generate a new hybrid strategy combining their respective strengths. Of 26 generated hybrid models (‘Recombination (TS)’), 11 achieved a WIS score superior to both of their parent models (Extended Data Fig. 7). We manually verified that output code for all recombination experiments contained relevant aspects from both parent codes (Supplementary Table 11). Finally, we used Gemini Deep Research^34^ and AI co-scientist^35^ to generate new forecasting ideas, which were then implemented using ERA. In total, this systematic exploration yielded 14 distinct strategies that outperformed the official CovidHub-ensemble: ten from recombination, two from Deep Research, one from AI co-scientist and one of our replicated baselines. Cosine similarities between embeddings for each generated code show clustering between different methods (Supplementary Fig. 9). An extended performance plot including the nine tree search models and three other submissions that did not outperform the baseline, alongside their corresponding validation performance, is provided in Supplementary Figs. 10 and 11. Examples of prediction curves are shown in Supplementary Fig. 12.

A deeper analysis of these 14 top-performing strategies reveals key patterns in how ERA achieves superior performance. The recombination models, which constitute most of the winners, highlight a clear pattern of synergistic hybridization. Two base models appear most frequently in these successful hybrids: the simple, climatology-based CMU-climate_baseline and the statistical autoregressive model UMass-ar6_pooled. This suggests that ERA consistently discovers that the most effective strategies are built on a robust foundation of historical averages and recent trends, which are then enhanced by more complex methods. Indeed, the most successful recombinations consistently fused different modelling patterns: for instance, pairing the epidemiological CEPH-Rtrend_covid model with the statistical UMass-ar6_pooled model created a hybrid anchored in the theory of disease spread yet highly responsive to recent data trends, whereas pairing the powerful machine learning UMass-gbqr model with the stable CMU-climate_baseline provided a robust seasonal foundation that allowed the machine learning model to safely focus on learning short-term deviations. Finally, we verified the importance of using ERA, rather than just taking the best of 128 solution attempts, for both epidemiological modelling and scRNA-seq batch integration (Table 1).

**Table 1:** Performance comparison of ERA and best of *N* on the scRNA-seq batch integration and epidemiological modelling tasks

Model	Method	Batch integration (higher better)	Epidemiology (lower better)
Gemini 3 Flash	BoN	0.6306	106.55
	ERA	**0.6626**	**84.11**
Mistral 3	BoN	0.6129	95.73
	ERA	**0.6387**	**87.21**
Claude Sonnet 4.6	BoN	0.6502	82.64
	ERA	**0.6638**	**69.58**
GPT-5	BoN	**0.6740**	84.94
	ERA	0.6548	**69.11**
Gemini 3.1 Pro	BoN	0.6679	78.07
	ERA	**0.6773**	**60.56**

Performance is measured on the validation set for each task. Bold entries indicate better performance. BoN, best of *N* = 128.

## Time-series forecasting: GIFT-Eval

We next evaluated ERA using General Time Series Forecasting Model Evaluation (GIFT-Eval)^19^, a standard benchmark for time-series forecasting. GIFT-Eval includes 28 datasets across seven diverse domains, with data frequencies ranging from seconds to years. It receives about four new submissions per month that span diverse methodologies, including black-box deep learning methods and foundation models. Submissions are scored on official train/validation/test splits using a normalized mean absolute scaled error (MASE) metric, calculated relative to a seasonal naive baseline.

We applied ERA in two phases. We began with a per-dataset solution in which ERA discovers an independent solution for each dataset. The second unified solution created a single general-purpose forecasting model using only basic libraries by hill-climbing against the average score for the entire GIFT-Eval.

### Per-dataset solution

Here we allowed ERA to use a full suite of Python libraries, including scikit-learn, statsmodels and XGBoost. ERA outperformed the entire 18 May 2025 leaderboard, which included foundation models, deep learning models and standard time-series methods (Supplementary Table 12). The discovered solutions showed strong convergence towards gradient boosting and ensemble/decomposition models (Supplementary Fig. 13).

### Unified solution

**Extended Data Fig. 8:**
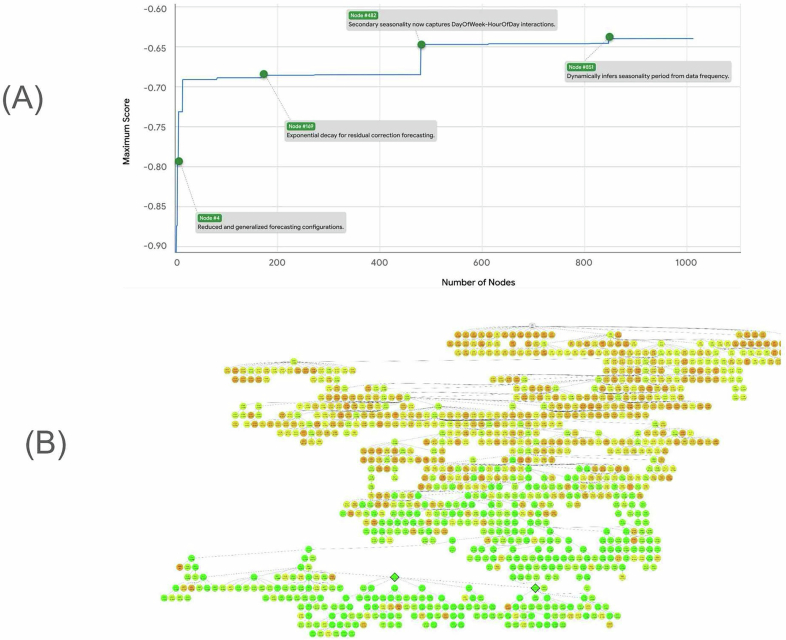
Breakthrough plot and solution tree for the time-series prediction task. **a**, Breakthrough plot for the GIFT-Eval tree search, showing the evolution of the maximum score as a function of the number of nodes. The green dots label places at which the score abruptly increases as a result of an improvement in the code and the label describes the change in the code that resulted in the score increase. **b**, Structure of the tree for this same search. The colour range consists of orange (lower scores) to green (higher scores), with the highest score denoted by a diamond node.

We considered whether the code mutation system could create a unified, general-purpose forecasting library from scratch, by hill-climbing with a single code on the average MASE on the entire GIFT-Eval dataset. To manage the diversity of the benchmark, we allowed the library to have an adaptive configuration system, by which it could generate up to eight preset hyperparameter configurations to adapt to the diversity of datasets, with a validation step selecting the best-performing configuration for each dataset. As the search progressed, date and trend-related features often led to performance breakthroughs, leading to a model that sequentially forecasts and subtracts individual time-series components, including a base level, trend, seasonality, datetime-based features and a final residual correction (Extended Data Fig. 8). The configurations (Supplementary Table 13) include date-specific features, including one that featurizes holidays in a specific set of countries ([‘US’, ‘DE’, ‘CN’, ‘GB’, ‘CA’, ‘AU’]). The resulting unified solution was highly competitive on the May 2025 leaderboard (Supplementary Table 12).

## Neuroscience and geospatial and numerical analysis

We also evaluated ERA on problems from three more distinct domains: geospatial semantic segmentation, vertebrate neural activity forecasting and numerical analysis (Supplementary Information). ERA achieved expert-level performance in each case (Supplementary Figs. 14–20 and Supplementary Table 14).

## Discussion

Our work introduces ERA, an AI-based system that drives a tree search with a LLM to systematically create and improve software for scientific tasks. By defining the problem of creating scientific software as a search for a program whose output maximizes a quality score, we convert software creation into a ‘scorable task’, producing empirical software. ERA is innovative in its LLM-driven rewriting approach, which allows for the flexible integration of domain knowledge and external research ideas. The ability of frontier LLMs to closely follow instructions enables efficient exploration of research ideas. ERA builds on ideas from several distinct but related areas of research: genetic programming, generative programming, the application of LLMs to code, automated machine learning (AutoML), combining LLMs and search and agents for scientific discovery.

Genetic programming: genetic programming provides a foundation to our work. In genetic programming, a population of programs is iteratively improved using evolutionary principles, such as selection, crossover and mutation. The fitness of each program is determined by a ‘fitness function’, which is directly analogous to our ‘quality score’^36^. Although genetic programming has been successful, it traditionally relies on random mutations and structured recombination of code fragments (for example, swapping subtrees in an abstract syntax tree). Before the widespread adoption of LLMs, genetic programming systems successfully incorporated human expertise by restricting the search space using formal grammars or making use of structural code metrics to bias the evolutionary search^37^. A key difference in ERA is the use of a LLM to perform intelligent, semantic-aware ‘mutations’ by rewriting the code, which can produce more complex and meaningful variations than the random changes typical in genetic programming.

Generative programming: ERA can be viewed as a modern AI-driven realization of traditional generative programming, in which a developer creates a program generator (using techniques such as templates, domain-specific languages^38^ or metaprogramming) that produces tailored source code for a family of related problems^39^. By contrast, we use a LLM guided by a tree search as the generative engine. This approach offers greater flexibility, allowing ERA to synthesize new programs by exploring a vast solution space and integrating diverse domain knowledge in ways not easily achievable with more template-based methods.

LLMs for code generation: the advent of LLMs pretrained on vast code corpora has revolutionized code generation. Systems such as AlphaCode^40^ and OpenAI Codex^41^ have demonstrated the ability to generate correct and complex code from natural language descriptions. These systems are typically used for ‘one-shot’ generation from a prompt. Our approach differs by using the LLM in an iterative refinement loop. Instead of generating code from scratch, our LLM rewrites existing software candidates, guided by a search algorithm (tree search) that uses the quality score as a signal.

AutoML: AutoML systems aim to automate the process of building machine learning pipelines by searching for optimal model architectures and hyperparameters. The goal is to maximize a performance metric (for example, accuracy, F1 score) on a validation dataset^42^, which fits our definition of a scorable task. Although AutoML focuses specifically on finding the best model within a fixed set of machine learning frameworks, ERA is more general. It can rewrite any software, including pre-processing steps, complex simulations, mathematical heuristics and other areas that fall outside the typical scope of AutoML.

Combining LLMs and search: the most closely related work involves combining LLMs with search algorithms to overcome the limitations of one-shot generation. A rapidly growing body of literature formulates automated algorithm design as an evolutionary algorithm^43,44^. FunSearch uses a LLM to search for new mathematical discoveries by pairing a LLM suggesting code improvements with an automated evaluator^15^. Very recently, agentic frameworks such as AlphaEvolve^14^ have expanded these evolutionary coding loops towards needle-in-the-haystack discovery problems, similar in spirit to the tree search algorithms explored here, albeit without idea exploration required for scientific problem-solving.

Agents for science problems: this subfield has seen notable performance from highly specialized systems, with agents focusing on problems ranging from data science^45^ to computational biology^46^. Instead of specializing in one domain, ERA demonstrates a general capability for empirical software optimization, achieving expert-level performance on public leaderboards and in academic literature across several fields.

To our knowledge, this is the first work demonstrating a system that beats human performance on a wide range of relevant and well-studied scientific problems. Across different fields, by prompting with the method description from cutting-edge papers, ERA not only produces code that follows the methods of the paper but often has superior results.

We wish to emphasize the critical distinction between optimizing empirical predictive models and performing genuine scientific discovery, the latter of which requires reasoning about underlying theories, causal mechanisms and mathematical frameworks. Although the core problems evaluated in this paper primarily emphasize advanced empirical software engineering to allow for rigorous, automated scoring, our underlying system goes well beyond this, towards scientific discovery (Supplementary Information).

The ability of LLM-based systems to autonomously produce expert-level empirical software carries broader safety risks, particularly when applied to domains that could directly affect human well-being. By automating complex engineering workflows, our system greatly lowers the technical expertise required to execute sophisticated computational tasks. Although this democratization accelerates beneficial scientific discovery, it concurrently lowers the barrier to entry for deploying advanced models in sensitive or potentially dangerous domains. This phenomenon represents a broader, systemic risk associated with the combination of large-scale inference-time compute and exponentially increasing foundation model quality.

To summarize, ERA combines a code mutation system based on tree search^2^ with the ability to integrate complex research ideas. Such research ideas could come from the published literature, from research agents (for example, refs. ^34,35^) or from combining previous ideas and solutions that the LLM has found itself. Because ERA creates code that can follow a specific idea, it can search over externally supplied research ideas. ERA created 40 methods that beat the best known method for scRNA-seq batch integration and 14 methods that outperformed the CDC ensemble for epidemiological prediction. Furthermore, ERA achieved expert-level performance on geospatial reasoning, neural activity prediction and algorithms for computational mathematics and a new rule-based strategy for time-series prediction.

Trial and error is essential to scientific progress, both for humans and for the automated approaches we outline here. ERA generates expert-level solutions very quickly, reducing exploration of a set of ideas from weeks or months to hours or days. Accelerating research in this way has profound consequences for scientific advancement. On the basis of this work, we believe that progress in scientific fields in which solutions can be scored by machines is on the precipice of a substantial acceleration.

## Methods

### Code mutation system

We prompt a LLM providing a description, the evaluation metric and the relevant data. The LLM produces Python code, which is then executed and scored on a sandbox. Searching over strategies strongly increases performance: the system uses the score together with output logs and other information to hill-climb towards a better score. We used a tree search strategy with an upper confidence bound (UCB) inspired by AlphaZero^47^. A critical difference from AlphaZero is that our problems do not allow exhaustive enumeration of all possible children of a node, so every node is a candidate for expansion. We therefore modify the UCB algorithm to count visits and compute mean values using the tree. However, when sampling a node to expand, we sample directly from the whole set instead of recursing from the root like AlphaZero. This makes our method closer to Flat-UCB^48^ than any Monte Carlo tree search variant, such as AlphaZero.

We also note that the algorithm differs from traditional tree search in that the scoring of the nodes does not involve random rollouts (for example, of a game) to estimate the value of a node. Yet there is still randomness for scoring each node, caused by the sampling of the LLM itself, which produces a distribution of different codes (scores) for each fixed prompt.

We use a predictor + UCB applied to trees (PUCT) tree search algorithm to explore the space of code^2^. The PUCT algorithm is described in Algorithm 1. For tree *T* and executed candidate *u*, we define the flat prior ${P}_{T}(u)=\frac{1}{|T|}$. To make it easier to tune the exploration constant *c*_puct_ across tasks, we convert task-specific scores TaskScore(*u*) to rank scores RankScore_*T*_(*u*) in the PUCT formula. We define ${{\rm{RankScore}}}_{T}(u)=\frac{{{\rm{Rank}}}_{T}(u)-1}{|T|-1}$ when |*T*| > 1 and 1 otherwise, in which Rank_*T*_(*u*) gives ascending-order ranks to the candidates.

To select the next node for expansion, the algorithm balances exploitation of high-scoring solutions with exploration of the search space by computing a PUCT-inspired (polynomial upper confidence trees) acquisition score for each node *i*:

1 $${{\rm{PUCT}}}_{i}={r}_{i}+{c}_{{\rm{puct}}}\times E(i)$$

in which *c*_puct_ is the exploration constant and *E*(*i*) is an exploration term based on the visit count of the node relative to the total number of visits across the entire tree. We tuned *c*_puct_ on the Kaggle benchmark to maximize performance, finding that *c*_puct_ = 1 works well. The node with the globally maximum PUCT_*i*_ score is selected. The language model then generates a single new child solution conditioned on the code and score of this parent. The new code is executed, assigned an empirical score and appended to the search tree, followed by a backpropagation of the visit count to its ancestors. This global, flat-tree structure allows the system to seamlessly backtrack and branch from any historical node if the present optimization path plateaus.

We emphasize that, in our algorithm, mutations occur at the code level—ideas are part of prompts that produce code, which is then scored and iterated on. With increasing nodes in the tree, the score saturates after 300–1,000 nodes (see breakthrough plots in the Supplementary Figs.). We search over ideas by combining different tree searches with different prompts together.

Finally, the implementation we outline here was chosen for optimal performance on the Kaggle benchmark. Many alternatives were explored, including different agentic configurations, and the simple implementation outlined here had the highest overall performance across the benchmark. All experiments in this paper were carried out with Gemini 2.5 Flash, with the improvement when using Gemini 2.5 Pro being only modest.

#### Algorithm 1

UCB tree search (PUCT)

**Require:** GenerateAndExecute(), TaskScore() to define rank scores RankScore_*T*_(*u*), exploration constant *c*_puct_ and a root node *r*.

1: *T* ← {*r*} ⊳ Initialize the tree with a root node.

2: *V*(*r*) ← 1

3: **for all** iterations **do**

4: *N*_total_ ← ∑_*u*∈*T*_*V*(*u*) ⊳ Get total visits across all nodes

5: Select ${u}^{\ast }\leftarrow {{\rm{argmax}}}_{u\in T}\left({{\rm{RankScore}}}_{T}(u)+{c}_{{\rm{puct}}}{P}_{T}(u)\frac{\sqrt{{N}_{{\rm{total}}}}}{1+V(u)}\right)$ ⊳ Select node with highest PUCT score

6: *u*_*c*_ ← GenerateAndExecute(u*) ⊳ Expand the Selected node and Execute

7: *T* ← *T* ∪ {*u*_*c*_}

8: *V*(*u*_*c*_) ← 1

9: **for all** ancestors *u*_*a*_ of *u*_*c*_ (excluding *u*_*c*_) **do** ⊳ Backpropagate results

10: *V*(*u*_*a*_) ← *V*(*u*_*a*_) + 1

11: **end for**

12: **end for**

13: **return** argmax_*u*∈*T*_TaskScore(*u*) ⊳ Best solution found

It is useful to explicitly contrast this algorithm with standard heuristic search algorithms. Our approach does not rely on beam search (which aggressively prunes unselected candidates at each depth layer) nor a (1 + *λ*) evolution strategy (which maintains only the most recent generation and discards historical states).

Similarly, our system differs from emergent efforts to incorporate LLMs into genetic programming^43,44,49–51^.

More recently, agentic AutoML frameworks encompass automated feature engineering, meta-learning, dynamic pipeline construction and multi-objective optimization^52^, including agentic systems^53,54^, which have shown more impressive performance^55^.

### Adding research ideas to the code mutation system

When an expert solves difficult scientific problems, they often search for previous work for ideas. Previous work could be sourced from highly cited papers, specialized textbooks or search engines. The search for previous work can also be powered by LLMs^34,35,56–60^.

We emulate the expert behaviour by injecting instructions for carrying out research ideas into the prompt of our code mutation system (Fig. 1). We applied the research instruction injection for scRNA-seq batch integration, COVID prediction, segmenting remote sensing images and whole-brain neural activity prediction. Although the most successful outcomes used top methods from the literature, we also used two LLM-driven search strategies: Deep Research from Gemini 2.5 Flash^34^ and AI co-scientist^35^.

For running these searches, we provided the tools with background information from the main problem description and instructed the models to create distinct ideas (Supplementary Table 15). After manually filtering proposals and removing one proposed scRNA-seq batch integration method, we prompted Gemini to format the ideas into a structure consistent with our baseline method descriptions (Supplementary Table 16). Finally, we ran ERA on these ideas to create empirical codes that could be scored.

### ERA versus best of *N*

We use *N* = 128 ERA search nodes and compare the performance of Gemini 3 Flash, Mistral 3, Claude Sonnet 4.6, GPT-5 and Gemini 3.1 Pro on the scRNA-seq batch integration task and an epidemiological flu forecasting task. This explores a range of models that were contemporaneous with the core model used in this paper while also using Gemini 3.1 Pro as a state-of-the-art model at the time of paper publication. The scores use the same scoring rules as in all other experiments: for the epidemiological forecasting task (flu forecasting), it is the WIS evaluated over a rolling evaluation window, whereas for scRNA-seq batch integration, it is the average of metrics measuring preservation of biological information while eliminating batch effects, both measured on the validation set. Note that marked improvements in batch integration happen with much smaller change in validation scores than in epidemiological forecasting. We present here the best performance over three different experiments (with both best of *N* and ERA). Typical variation in performance is on the order 0.01 for batch integration and *O*(1) for epidemiological forecasting, with some model dependence. For example, GPT-5 has much more experiment-to-experiment variability than other models.

We note that ERA explores the solution space more efficiently than best of *N* and outperforms best of *N* for all models and problems, with the exception of the performance of GPT-5 on batch integration. For the batch integration problem, the one-shot performance of GPT-5 is good enough that this distinction is not important. Indeed we expect that, as frontier models improve, tasks that were once difficult will saturate. Tree search is useful for pushing model performance on difficult tasks.

### Recombination experiments

For both the scRNA-seq batch integration problem and COVID-19 forecasting, we combined ideas from methods already generated using tree search. For the scRNA-seq batch integration problem, we used the first versions of our 11 baseline methods. For the COVID-19 prediction problem, we used the eight replications of models submitted to CovidHub. We first took the top-performing node from each tree search run seeded with one of these methods, based on its score on the validation set (for COVID-19 prediction, this included 6 weeks of reference dates from 22 February 2025 to 29 March 2025). Then, for every pair of these methods, we prompted Gemini 2.5 Flash to compare the two methods and explain the core technical similarities and differences between the two parent models using a consistent prompt (Supplementary Table 7). The explanatory response was then added to the prompt, along with a statement instructing tree search to recombine the ideas by combining the best parts of both approaches (Supplementary Table 17). Subsequently, we ran ERA to generate new hybrid strategies. This process yielded 55 recombined methods for the scRNA-seq batch integration problem and 28 for the COVID-19 prediction problem (evaluated on the 3-week holdout set 5 April 2025 to 19 April 2025; Fig. 3e).

Our method for recombination differs from previous efforts in which the LLM acts as a mutation and crossover operator^49–51,61–64^. Our approach to recombining expert ideas is conceptually related to evolutionary methods such as rolling horizon evolutionary algorithms (RHEA), with the comparative advantage that our tree search recombines expert ideas directly in conceptual space by means of LLM prompts rather than solely in distilled model space^65^.

### Gemini embeddings

For each tree search implementation, we input the code snippets to the Gemini text embedding model^66^ and the resulting 3,072-dimensional output vectors served as the semantic representations of their respective implementations.

### scRNA-seq batch integration

For all scRNA-seq experiments, we ran tree search with 500 nodes. Each experiment took roughly 7 h to execute on our infrastructure.

#### Dataset

We sourced a dataset from CZ CELLxGENE Discover^25^ to use for hill-climbing with tree search. To identify datasets distinct from the six OpenProblems.bio test datasets but have similar characteristics, we filtered to datasets that contain only healthy human cells, with primary cell count ≥ 2,000, at least ten unique cell types, at least seven unique donor IDs (that is, number of batches) and contain at least two unique assays that are also present in the OpenProblems.bio datasets. This filtering process identified 22 candidate datasets. After manually investigating the candidate datasets, we selected the dataset 364bd0c7-f7fd-48ed-99c1-ae26872b1042 version ffdaa1f0-b1d1-4135-8774-9fed7bf039ba (ref. ^17^).

Within the selected dataset, we applied quality-control metrics and data-processing steps identical to the processing performed on the OpenProblems.bio datasets^67,68^, yielding a processed dataset with normalized expression values, highly variable genes, principal components and *k*-nearest neighbours all computed. For computational efficiency, we randomly selected two disjoint subsets of *N* = 20,000 cells each, attempting to match (batch, cell type) distributions of the entire processed dataset. The ‘train’ dataset was used for model training and selection of the highest-performing node in a single tree search. The ‘validation’ dataset was used to select the best tree search for methods in which we ran several replicates of the same algorithm (Supplementary Fig. 1).

#### Evaluating scRNA-seq batch integration on the OpenProblems.bio benchmark

We downloaded the OpenProblems v2.0.0 input and solution data from s3://openproblems-data/resources/task_batch_integration/datasets/cellxgene_census/ and raw performance metrics from s3://openproblems-data/resources/task_batch_integration/results/run_2025-01-23_18-03-16/score_uns.yaml. We computed control-scaled metric results identically to the published OpenProblems results. Briefly, for each (dataset, metric), lower and upper bounds on raw scores are defined as the minimum and maximum values achieved by the seven ‘control’ methods. Raw values were linearly scaled between those extrema and clamped to be in [0, 1]. The overall score was computed as the arithmetic mean over all 78 measurements (13 metrics computed for each of six datasets) with not a number (NaN) values replaced by 0 (that is, failure to compute a metric causes it to be considered the worst possible score).

#### Replication of existing methods for batch integration

The OpenProblems.bio benchmark profiles the performance of several state-of-the-art existing methods. As of 11 July 2025, there were 19 different methods. Three methods have implementations in both R and Python: LIGER and pyliger, Harmony and Harmonypy and batchelor mnnCorrect and mnnpy. After grouping reimplementations of the same method, there are 16 separate research ideas. From this list, we excluded all six foundation model methods (UCE, SCimilarity, scGPT (zero shot), scGPT (fine-tuned), Geneformer and scPRINT) because they perform very poorly on the benchmark and use a much larger training set. For example, only a single foundation model (UCE) performs better than the negative control of ‘no integration’, which simply performs PCA on the dataset. We further excluded scANVI, which is a modification of scVI, which is trained using cell-type information. Because cell-type information is used to define the metrics, this represents data leakage and, consequently, we consider scANVI a control method. This resulted in nine existing different research methods to optimize with tree search.

For each of the nine existing methods, we obtained the manuscript PDF corresponding to the method. To obtain a short method description from the manuscript, we used Gemini 2.5 Pro Thinking to summarize the paper (prompt in Supplementary Table 18, example output in Supplementary Table 19). For batchelor fastMNN, which is a faster implementation of batchelor mnnCorrect, there is no separate publication and, thus, we provided the paper PDF of batchelor mnnCorrect as well as the docstring corresponding to batchelor fastMNN from https://rdrr.io/bioc/batchelor/man/fastMNN.html (‘Details’ section) with a slightly adjusted prompt. Finally, the method summary is added to the tree search prompt and is used to discover better code solutions given the method summary.

For each of the nine methods, we ran three replicates of tree search. For Fig. 2, we selected the replicate that had the best performance based on the validation set score. We show the performance of all replicates in Extended Data Fig. 1. Code for the best-performing method is shown in Supplementary Table 6.

#### Hyperparameters

To determine optimal hyperparameters for each base method, we used Optuna, an automated hyperparameter optimization framework^69^. Search spaces were defined across integer, float and categorical parameter types by experts. The optimization process ran for a total of five times the number of parameters. In each trial, a model was trained using a sampled parameter set and evaluated on the basis of a performance metric that Optuna’s tree-structured Parzen estimator sampler aimed to maximize. All hyperparameter optimization was conducted only on the training dataset. The best identified hyperparameter set was then used to train the final base methods and evaluate them on the held-out OpenProblems dataset.

### COVID-19 prediction

#### Dataset

Our primary data source was historical confirmed COVID-19 hospital admissions, which corresponds to the target variable specified by CovidHub. These data are published weekly by the CDC within the National Healthcare Safety Network (NHSN) Hospital Respiratory Data (HRD) dataset^70^. Preprocessing was kept minimal—missing values in the dataset were replaced by zeros to enable tree search to find executable code with the criterion score (WIS). The only extra data source used to augment the target for our model was static jurisdiction-specific population values from the CovidHub GitHub repository^18^. For comparing model performance in Fig. 3c, we use all of the models submitted to Forecast Hub that make predictions at a state-by-state level and have forecasts for at least 75% of the season and time horizons. We ran tree search with 2,000 nodes for each reported run. We note that we use data available as of 1 May 2025 for the entire retrospective season, thus ignoring potential differences in data available at the date of forecast.

#### Replication of existing COVID-19 prediction models

We selected eight models for replication from those that had submitted to CovidHub based on the following inclusion criteria: (1) the method must be reproducible solely using historical COVID-19 hospitalization data, without reliance on external predictor variables; (2) the model submission must include predictions across all specified time horizons; and (3) model submissions must be available for more than 3 months (12 weeks) to enable meaningful comparison. Three models were excluded for failing these criteria: two were ensembles of external forecasts and one relied entirely on further data. Five more models were excluded because they did not provide predictions for all forecast horizons. These five models originated from the same forecasting team. As all of our analysis involves aggregating model performance across horizons, we have excluded these five models from all comparisons. Overall, this gave a selection of eight models for replication.

To instruct the search algorithm, we provided the method descriptions from the original authors’ official submission metadata. For example, the metadata for the UMass-ar6_pooled model states: “AR(6) model after fourth root data transform. AR coefficients are shared across all locations. A separate variance parameter is estimated for each location.” We integrated these concise descriptions directly into the tree search prompt as part of the model directions, transforming them into instructions by prepending ‘Use a/an’ (see Methods and Supplementary Table 10).

### GIFT-Eval benchmark

We applied our tree search methodology to the GIFT-Eval benchmark^19^. The search begins from a root node defined by an initial code template and proceeds by means of hill-climbing, in which new candidate solutions are generated and evaluated against the GIFT-Eval validation folds. At the end of a tree search, we evaluated the solution on the held-out test set using MASE point forecast as the scoring metric. Our results are based on a 18 May 2025 snapshot of the dataset, official leaderboard and scoring, all of which have been updated since. See Supplementary Table 12 for a complete snapshot of the leaderboard.

We remark that our snapshotting to a fixed date ensures stability. The GIFT-Eval protocols underwent substantial structural changes after our experiments concluded, including notable scoring/dataset corrections on 24 July 2025 and the introduction of an ‘Agentic’ category on 5 August 2025. Later updates on 25 August 2025 redefined ‘Zero-shot’ to account for widespread test data leakage in foundation models using the large pretrain dataset (note that our method does not use the large pretraining data). To avoid unsound comparisons against baselines developed under these revised protocols, we deliberately chose not to submit to the live public leaderboard, restricting our comparison with the 18 May 2025 snapshot to ensure a valid, stable baseline. We also note that Gemini models driving our tree search have evolved substantially since our experiments.

We stood by the framework of the benchmark, using the official dataset source from Hugging Face (https://huggingface.co/datasets/Salesforce/GiftEval), its predefined training, validation and test splits, as well as the scoring and evaluation code commonly used in the existing submission notebooks (https://github.com/SalesforceAIResearch/gift-eval/tree/main/notebooks).

#### Per-dataset solution

We conducted separate tree searches for 92 of the 97 GIFT-Eval datasets, excluding the five largest owing to computational constraints; for these, the naive baseline score was used to produce the aggregated leaderboard score. For each dataset, we used a search of 300 nodes, with the system permitted to use a broad suite of machine learning libraries, including scikit-learn, XGBoost and statsmodels. Supplementary Fig. 13 shows an analysis of the types of model used across the 92 different solutions.

#### Unified solution

Here we created a single, unified forecasting library that could generalize across all 97 datasets. We used a tree search of more than 1,000 nodes, guided by the geometric mean of the normalized MASE scores across all datasets, providing a single objective function to optimize. To force the model to reason from first principles, its access was restricted to basic libraries (numpy, pandas and holidays).

The resulting solution consists of two components: a single forecasting library and a list of eight preset configurations. For each dataset, the best-performing configuration is identified on the validation set. This selected configuration is then used with the unified library to produce the final forecast on the test set, allowing the model to adapt its strategy without seeing test data.

The final solution was developed iteratively. An initial search yielded a base model with a MASE of 0.82. A key breakthrough occurred in a subsequent run when the search space was expanded to ten configurations and the system was advised to use the holidays library, which improved the MASE to 0.77 (Extended Data Fig. 8). A final 500-node refinement run pruned the configurations to an optimized set of eight, achieving the final MASE of 0.734.

The final solution sequentially models and removes fundamental components of the series, with the final forecast being the sum of the individual component forecasts. This approach allows the model to be highly configurable while systematically accounting for different sources of variation in the data. This process is outlined with the following steps:

1. **Preprocessing:** the input series first undergoes basic cleaning, including median imputation for any missing values. An optional log-transform (log1p) can be applied to stabilize variance in series with exponential growth patterns.
2. **Base/level component:** a base level is established using simple but robust methods such as a seasonal naive forecast or a rolling median of recent data points. This component captures the basic magnitude of the series.
3. **Trend component:** the residuals from the base component are then modelled to capture linear or polynomial trends. This step includes a damping_factor to prevent unrealistic long-term extrapolation by gradually flattening the trend.
4. **Seasonality component:** the residuals from the trend component are analysed to model cyclical patterns (for example, weekly, yearly). The model identifies the cycle length and forecasts seasonality by averaging values at the same point in the cycle (for example, the average value for all Mondays).
5. **Datetime and holiday features:** to capture special events and non-seasonal cycles, features are extracted from the timestamp (for example, dayofweek, is_holiday_flag). The model calculates the median effect of each feature category from the remaining residuals and adds it to the forecast.
6. **Residual correction:** as a final step, a correction is made by modelling the median of the most recent unexplained errors. This autoregressive-like step helps correct for short-term biases in the model. A decay_factor fades its impact over the forecast horizon.

To apply the unified solution to a new dataset, we would first split the historical data into training and validation sets. Using the library’s adaptive configuration system, we can then find a suitable forecasting strategy by evaluating the eight preset configurations on the validation data to select the best-performing one. This provides a strong, data-driven starting point that can be used directly. For more specialized applications, we can also create a custom configuration, allowing for manual refinement of the model’s components and making the library both powerful out of the box and flexible enough for expert tuning.

## Online content

Any methods, additional references, Nature Portfolio reporting summaries, source data, extended data, supplementary information, acknowledgements, peer review information; details of author contributions and competing interests; and statements of data and code availability are available at 10.1038/s41586-026-10658-6.

## Supplementary information

Supplementary Information

Peer Review File

## Figures and Tables

**Extended Data Table 1:**
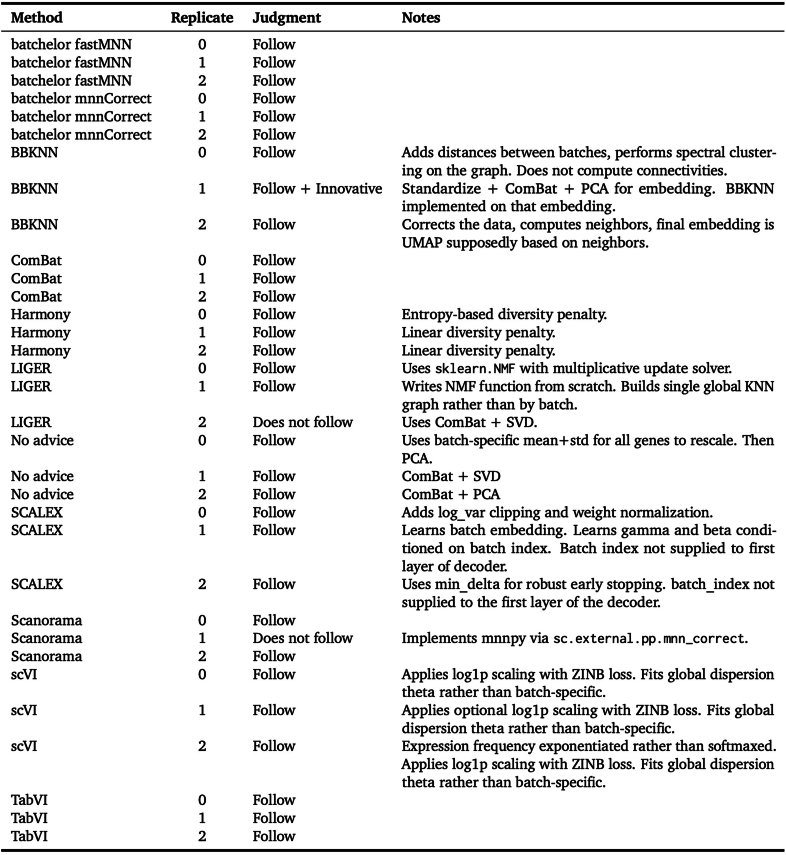
Expert manual inspection of adherence of ERA implementation to scRNA-seq batch integration method

## Data Availability

A reference implementation of ERA is available at https://github.com/google-research/era. The best candidate solutions generated for each of the six scientific problems in this paper are publicly available at https://google-research.github.io/era, along with a user interface enabling examination of the full tree search data for a representative run for each of the six scientific problems. The interface allows inspecting the solution progression and breakthrough plot as the tree search proceeds and highlights code diffs.

## References

[1] Hannay, J. E. et al. How do scientists develop and use scientific software? In *Proc. 2009 ICSE Workshop on Software Engineering for Computational Science and Engineering* 1–8 (IEEE, 2009).

[2] Silver, D. et al. Mastering the game of Go with deep neural networks and tree search. *Nature* **529**, 484–489 (2016). 26819042 10.1038/nature16961

[3] Hohenberg, P. & Kohn, W. Inhomogeneous electron gas. *Phys. Rev.* **136**, B864 (1964).

[4] Kohn, W. & Sham, L. J. Self-consistent equations including exchange and correlation effects. *Phys. Rev.* **140**, A1133 (1965).

[5] Warshel, A. & Levitt, M. Theoretical studies of enzymic reactions: dielectric, electrostatic and steric stabilization of the carbonium ion in the reaction of lysozyme. *J. Mol. Biol.* **103**, 227–249 (1976). 985660 10.1016/0022-2836(76)90311-9

[6] Jumper, J. et al. Highly accurate protein structure prediction with AlphaFold. *Nature* **596**, 583–589 (2021). 34265844 10.1038/s41586-021-03819-2 PMC8371605

[7] Baek, M. et al. Accurate prediction of protein structures and interactions using a three-track neural network. *Science* **373**, 871–876 (2021). 34282049 10.1126/science.abj8754 PMC7612213

[8] Hourdin, F. et al. The art and science of climate model tuning. *Bull. Am. Meteorol. Soc.* **98**, 589–602 (2017).

[9] Anderson, J. Jr. in *Computational Fluid Dynamics* (ed. Wendt, J. F.) 3–14 (Springer, 2009).

[10] Silver, N.* The Signal and the Noise: Why So Many Predictions Fail – But Some Don’t* (Penguin, 2012).

[11] Farmer, J. D. *Making Sense of Chaos: A Better Economics for a Better World* (Yale Univ. Press, 2024).

[12] Sculley, D. et al. Hidden technical debt in machine learning systems. In *Proc. 29th International Conference on Neural Information Processing Systems (NIPS’15)* (eds Cortes, C., Lee, D. D., Sugiyama, M. & Garnett, R.) 2503–2511 (MIT Press, 2015).

[13] Jiang, Z. et al. AIDE: AI-Driven Exploration in the space of code. Preprint at https://arxiv.org/abs/2502.13138 (2025).

[14] Novikov, A. et al. AlphaEvolve: a coding agent for scientific and algorithmic discovery. Preprint at https://arxiv.org/abs/2506.13131 (2025).

[15] Romera-Paredes, B. et al. Mathematical discoveries from program search with large language models. *Nature* **625**, 468–475 (2024). 38096900 10.1038/s41586-023-06924-6 PMC10794145

[16] Hu, S., Lu, C. & Clune, J. Automated design of agentic systems. In *13th International Conference on Learning Representations* 21344–21377 (2025).

[17] Xu, C. et al. Automatic cell-type harmonization and integration across Human Cell Atlas datasets. *Cell* **186**, 5876–5891 (2023). 38134877 10.1016/j.cell.2023.11.026

[18] Centers for Disease Control and Prevention (CDC). COVID-19 Forecast Hub. https://github.com/cdcgov/covid19-forecast-hub?tab=readme-ov-file (2025).

[19] Aksu, T. et al. GIFT-Eval: a benchmark for general time series forecasting model evaluation. In *NeurIPS Workshop on Time Series in the Age of Large Models* (2024).

[20] Shao, Z., Yang, K. & Zhou, W. Performance evaluation of single-label and multi-label remote sensing image retrieval using a dense labeling dataset. *Remote Sens.* **10**, 964 (2018).

[21] Lueckmann, J.-M. et al. ZAPBench: a benchmark for whole-brain activity prediction in zebrafish. In *13th International Conference on Learning Representations* 4784 (2025).

[22] Jovic, D. et al. Single-cell RNA sequencing technologies and applications: a brief overview. *Clin. Transl. Med.* **12**, e694 (2022). 35352511 10.1002/ctm2.694 PMC8964935

[23] Svensson, V., Vento-Tormo, R. & Teichmann, S. A. Exponential scaling of single-cell RNA-seq in the past decade. *Nat. Protoc.* **13**, 599–604 (2018). 29494575 10.1038/nprot.2017.149

[24] Regev, A. et al. The Human Cell Atlas. *eLife* **6**, e27041 (2017). 29206104 10.7554/eLife.27041 PMC5762154

[25] CZI Cell Science Program et al. CZ CELLxGENE Discover: a single-cell data platform for scalable exploration, analysis and modeling of aggregated data. *Nucleic Acids Res.* **53**, D886–D900 (2025). 39607691 10.1093/nar/gkae1142 PMC11701654

[26] Stuart, T. & Satija, R. Integrative single-cell analysis. *Nat. Rev. Genet.* **20**, 257–272 (2019). 30696980 10.1038/s41576-019-0093-7

[27] Zappia, L., Phipson, B. & Oshlack, A. Exploring the single-cell RNA-seq analysis landscape with the scRNA-tools database. *PLoS Comput. Biol.* **14**, e1006245 (2018). 29939984 10.1371/journal.pcbi.1006245 PMC6034903

[28] Tran, H. T. N. et al. A benchmark of batch-effect correction methods for single-cell RNA sequencing data. *Genome Biol.* **21**, 1–32 (2020). 10.1186/s13059-019-1850-9 PMC6964114 31948481

[29] Chazarra-Gil, R., van Dongen, S., Kiselev, V. Y. & Hemberg, M. Flexible comparison of batch correction methods for single-cell RNA-seq using BatchBench. *Nucleic Acids Res.* **49**, e42 (2021). 33524142 10.1093/nar/gkab004 PMC8053088

[30] Luecken, M. D. et al. Defining and benchmarking open problems in single-cell analysis. *Nat. Biotechnol.* **43**, 1035–1040 (2025). 40595413 10.1038/s41587-025-02694-w

[31] Johnson, W. E., Li, C. & Rabinovic, A. Adjusting batch effects in microarray expression data using empirical Bayes methods. *Biostatistics* **8**, 118–127 (2007). 16632515 10.1093/biostatistics/kxj037

[32] Polański, K. et al. BBKNN: fast batch alignment of single cell transcriptomes. *Bioinformatics* **36**, 964–965 (2019). 10.1093/bioinformatics/btz625 PMC9883685 31400197

[33] Chandrashekar, A. et al. TabVI: leveraging lightweight transformer architectures to learn biologically meaningful cellular representations. Preprint at *bioRxiv*10.1101/2025.02.13.637984 (2025).

[34] Google. Gemini Deep Research. https://gemini.google/overview/deep-research/?hl=en (2025).

[35] Gottweis, J. et al. Accelerating scientific discovery with Co-Scientist. *Nature*10.1038/s41586-026-10644-y (2026). 10.1038/s41586-026-10644-y PMC13345910 42156544

[36] Koza, J. R. Genetic programming as a means for programming computers by natural selection. *Stat. Comput.* **4**, 87–112 (1994).

[37] Schweim, D., Hemberg, E., Sobania, D., O’Reilly, U.-M. & Rothlauf, F. Using knowledge of human-generated code to bias the search in program synthesis with grammatical evolution. In *Proc. Genetic and Evolutionary Computation Conference Companion (GECCO**’21)* (ed. Chicano, F.) 331–332 (Association for Computing Machinery, 2021).

[38] Mernik, M., Heering, J. & Sloane, A. M. When and how to develop domain-specific languages. *ACM Comput. Surv.* **37**, 316–344 (2005).

[39] Czarnecki, K. in *Software Reuse: Methods, Techniques, and Tools (ICSR 2002)* (ed. Gacek, C.) 351–352 (Springer, 2002).

[40] Li, Y. et al. Competition-level code generation with AlphaCode. *Science* **378**, 1092–1097 (2022). 36480631 10.1126/science.abq1158

[41] Chen, M. et al. Evaluating large language models trained on code. Preprint at https://arxiv.org/abs/2107.03374 (2021).

[42] Hutter, F., Kotthoff, L. & Vanschoren, J. *Automated Machine Learning: Methods, Systems, Challenges* (Springer, 2019).

[43] Wu, X., Wu, S.-h., Wu, J., Feng, L. & Tan, K. C. Evolutionary computation in the era of large language model: survey and roadmap. *IEEE Trans. Evol. Comput.* **29**, 534–554 (2024).

[44] Ma, Z., Guo, H., Gong, Y., Zhang, J. & Tan, K. Toward automated algorithm design: a survey and practical guide to meta-black-box-optimization. *IEEE Trans. Evol. Comput.* **30**, 667–687 (2025).

[45] Ifargan, T., Hafner, L., Kern, M., Alcalay, O. & Kishony, R. Autonomous LLM-driven research—from data to human-verifiable research papers. *NEJM AI* **2**, AIoa2400555 (2024).

[46] Xiao, Y. et al. CellAgent: an LLM-driven multi-agent framework for automated single-cell data analysis. Preprint at https://arxiv.org/abs/2407.09811 (2024).

[47] Silver, D. et al. Mastering the game of Go without human knowledge. *Nature* **550**, 354–359 (2017). 29052630 10.1038/nature24270

[48] Coquelin, P.-A. & Munos, R. Bandit algorithms for tree search. In *Proceedings of the 23rd Conference on Uncertainty in Artificial Intelligence* 67–74 (2007).

[49] Hemberg, E., Moskal, S. & O’Reilly, U.-M. Evolving code with a large language model. *Genet. Program. Evolvable Mach.* **25**, 21 (2024).

[50] Meyerson, E. et al. Language model crossover: variation through few-shot prompting. *ACM Trans. Evol. Learn.* **4**, 1–40 (2024).

[51] Grishina, A., Liventsev, V., Härmä, A. & Moonen, L. Fully autonomous programming using iterative multi-agent debugging with large language models. *ACM Trans. Evol. Learn.* **5**, 1–37 (2025).

[52] He, X., Zhao, K. & Chu, X. AutoML: a survey of the state-of-the-art. *Knowl.-Based Syst.* **212**, 106622 (2021).

[53] Fang, Y. et al. MLZero: a multi-agent system for end-to-end machine learning automation. In *The 39th Annual Conference on Neural Information Processing Systems* (2025).

[54] Li, Z., Zang, Q., Ma, D. et al. AutoKaggle: a multi-agent framework for autonomous data science competitions. In *ICLR 2025 Workshop: Emergent Possibilities and Challenges in Deep Learning for Code* (2024).

[55] Grosnit, A. et al. Kolb-based experiential learning for generalist agents with human-level Kaggle data science performance. Preprint at https://arxiv.org/abs/2411.03562 (2024).

[56] Perplexity. Perplexity Deep Research. https://www.perplexity.ai/hub/blog/introducing-perplexity-deep-research (2025).

[57] Du, M., Xu, B., Zhu, C., Wang, X. & Mao, Z. DeepResearch Bench: a comprehensive benchmark for deep research agents. Preprint at https://arxiv.org/abs/2506.11763 (2025).

[58] Coelho, J. et al. DeepResearchGym: a free, transparent, and reproducible evaluation sandbox for deep research. Preprint at https://arxiv.org/abs/2505.19253 (2025).

[59] Xu, R. & Peng, J. A comprehensive survey of deep research: systems, methodologies, and applications. Preprint at https://arxiv.org/abs/2506.12594 (2025).

[60] Baek, J., Hwang, S. J., Jauhar, S. K. & Cucerzan, S. ResearchAgent: iterative research idea generation over scientific literature with large language models. In *2025 Annual Conference of the Nations of the Americas Chapter of the Association for Computational Linguistics* (Assoc. Comp. Linguistics, 2025).

[61] Lehman, J. et al. in *Handbook of Evolutionary Machine Learning* (eds Banzhaf, W., Machado, P. & Zhang, M.) 331–366 (Springer, 2023).

[62] van Stein, N. & Bäck, T. LLaMEA: a large language model evolutionary algorithm for automatically generating metaheuristics. *IEEE Trans. Evol. Comput.* **29**, 331–345 (2024).

[63] Liu, F. et al. Evolution of heuristics: towards efficient automatic algorithm design using large language model. In *Proc. 41st International Conference on Machine Learning (ICML**’24**)* (eds Salakhutdinov, R. et al.) 32201–32223 (JMLR, 2024).

[64] Ye, H. et al. ReEvo: large language models as hyper-heuristics with reflective evolution. In *Proc. 38th International Conference on Neural Information Processing Systems (NIPS**’24)* (eds Globerson, A. et al.) 43571–43608 (Curran Associates, 2024).

[65] Meyerson, E., Francon, O., Sargent, D., Hodjat, B. & Miikkulainen, R. Unlocking the potential of global human expertise. *Adv. Neural Inf. Process. Syst.* **37**, 119227–119259 (2024).

[66] Lee, J. et al. Gemini Embedding: generalizable embeddings from Gemini. Preprint at https://arxiv.org/abs/2503.07891 (2025).

[67] Gigante, S. et al. openproblems. https://github.com/openproblems-bio/openproblems (2025).

[68] Cannoodt, R. et al. task_batch_integration. https://github.com/openproblems-bio/task_batch_integration (2025).

[69] Akiba, T., Sano, S., Yanase, T., Ohta, T. & Koyama, M. Optuna: a next-generation hyperparameter optimization framework. In *Proc. 25th ACM SIGKDD Conference on Knowledge Discovery and Data Mining (KDD**’19)* 2623–2631 (Association for Computing Machinery, 2019).

[70] Centers for Disease Control and Prevention (CDC). Weekly Hospital Respiratory Data (HRD) Metrics by Jurisdiction. https://data.cdc.gov/Public-Health-Surveillance/Weekly-Hospital-Respiratory-Data-HRD-Metrics-by-Ju/mpgq-jmmr. Dataset ID: mpgq-jmmr. Last updated: 14 June 2024 (2024).

