# Supplementary material for: An AI system to help scientists write expert-level empirical software
Source: Nature. 2026 May 19;654(8120):909–16. doi: 10.1038/s41586-026-10658-6 (PMC13293872; doi:10.1038/s41586-026-10658-6)
Supplement: Supplementary file 1 — Supplementary Information [file 41586_2026_10658_MOESM1_ESM.pdf]

nature portfolio
https://doi.org/10.1038/s41586-026-10658-6

Supplementary information

An AI system to help scientists write expert-level empirical software

In the format provided by the authors and unedited

Supplementary Information for: An AI system to help scientists write expert-level empirical software

Eser Aygün[1,*], Anastasiya Belyaeva[2,*], Gheorghe Comanici[1,*], Marc Coram[2,*], Hao Cui[2,*], Jake Garrison[3,*], Renee Johnston[2,*], Anton Kast[2,*], Cory Y. McLean[2,*], Peter Norgaard[2,*], Zahra Shamsi[2,*], David Smalling[1,*], James Thompson[2,*], Subhashini Venugopalan[2,*], Brian P. Williams[2,*], Chujun He[2,4,], Sarah Martinson[2,5,**], Martyna Plomecka[2,6,**], Lai Wei[2], Yuchen Zhou[2], Qian-Ze Zhu[2,5,**], Matthew Abraham[2], Erica Brand[2], Anna Bulanova[1], Jeffrey A. Cardille[2,7], Chris Co[2], Scott Ellsworth[2], Grace Joseph[2], Malcolm Kane[2], Ryan Krueger[2,5,**], Johan Kartiwa[2], Dan Liebling[2], Jan-Matthis Lueckmann[2], Paul Raccuglia[2], Xuefei (Julie) Wang[2,8,**], Katherine Chou[2], James Manyika[2], Yossi Matias[2], John C. Platt[2], Lizzie Dorfman[2], Shibl Mourad[1,‡] and Michael P. Brenner[2,5,‡]**

[1]Google DeepMind, [2]Google Research, [3]Google Platforms and Devices, [4]Massachusetts Institute of Technology, [5]School of Engineering and Applied Sciences, Harvard University, [6]Google Cloud, [7]Faculty of Agricultural and Environmental Sciences, McGill University, [8]California Institute of Technology

Contents

1 Supplementary Notes 3
1.1 Geospatial Analysis: Segmentation of Remote Sensing Images 3
1.2 Neuroscience: Whole-Brain Neural Activity Prediction 3
1.3 Numerical Analysis: Library for Numerical Evaluation of Difficult Integrals . 5
1.4 Towards Genuine Discovery 6
1.5 Computational Cost and Resource Utilization 6

2 Supplementary Figures 7
Fig. S1: Experimental design for single-cell batch integration. 7
Fig. S2: Uniform Manifold Approximation and Projection of `BBKNN (TS)` on the Immune Cell Atlas dataset. 8
Fig. S3: Relative performance of base methods with optimized hyperparameters and ERA replicates. 8
Fig. S4: Heatmap of text embedding cosine similarities among ERA-generated methods. 9
Fig. S5: UMAP of text embeddings representing ERA-generated methods for single-cell batch integration. 10
Fig. S6: Performance of retrospective COVID-19 hospitalization forecasts across all replicates. 11
Fig. S7: Visual validation of COVID-19 hospitalization forecasts. 12
Fig. S8: Full-season COVID-19 forecasts across all validation splits. 13
Fig. S9: Heatmap of conceptual similarities among COVID-19 forecasting generated codes for methods. 14
Fig. S10: Performance of recombination experiments for COVID-19 forecasting. . . . 15
Fig. S11: Validation performance for COVID-19 forecasting. 16
Fig. S12: Examples of COVID-19 forecasts. 17
Fig. S13: Categories of solutions on the GIFT-Eval benchmark on the `per-dataset solution (v1)`. 18

Fig. S14: Example output segmenting DLRSD image pixels from ERA Solution 1 (U-Net++). 19
Fig. S15: Breakthrough plot for the geospatial segmentation task. 20
Fig. S16: Comparison of solutions to time-series and video forecasting methods across conditions on ZAPBench. 21
Fig. S17: Breakthrough plot and solution tree for the ZAPBench task. 21
Fig. S18: The dataset of 38 definite integrals with oscillatory integrands on semi-infinite domains. 22
Fig. S19: Breakthrough plot and solution tree for the numerical integration task. 23
Fig. S20: Scores of the best numerical integration routine applied to the held-out set of 19 integrals. 24

3 Supplementary Tables **25**
Table S1: Computational budget and execution costs per search node across evaluated benchmarks. 25
Table S2: Kaggle Playground Series (Season 3) competitions included in the experiments. 25
Table S3: Prompt for Kaggle Playground competitions. 26
Table S4: Expert advice for Kaggle Playground competitions. 27
Table S5: Boosted decision trees for Kaggle Playground competitions. 27
Table S6: Example code generated by ERA. 28
Table S7: Prompt for recombination of baseline method ideas. 30
Table S8: Configuration of COVID-19 forecasting data splits. 31
Table S9: Method descriptions used for replicating COVID-19 models submitted to the CDC's CovidHub. 32
Table S10: Prompt for replicating COVID-19 models submitted to CovidHub by injecting method descriptions as `{method}` into existing tree search prompt. 34
Table S11: Expert manual inspection of adherence of ERA implementation to COVID-19 modeling methods. 35
Table S12: Full GIFT-Eval leaderboard (05/18/2025 snapshot). 39
Table S13: Example configurations from the final `unified solution` for the GIFT-Eval task. 40
Table S14: Comparison of model performance on the DLRSD benchmark. 41
Table S15: Prompt for Gemini Deep Research to generate ideas to integrate single-cell batch effects. 42
Table S16: Prompt for formatting Deep Research ideas into a structure similar to baseline method descriptions. 43
Table S17: Prompt for guiding ERA to generate hybrid strategies. 44
Table S18: Prompt for summarizing existing batch integration methods. 45
Table S19: Example of existing method description (BBKNN) given to ERA. 46

4 Supplementary References **47**

1. Supplementary Notes

1.1. Geospatial Analysis: Segmentation of Remote Sensing Images

We ran ERA on a problem in geospatial analysis: semantic segmentation of high-resolution remote sensing images. Semantic segmentation is a computer vision task that involves assigning a specific class label to every single pixel in an image. It is essential for diverse applications, ranging from monitoring land use, assessing the environmental impacts of human activity and managing natural disasters. The primary difficulty is significant visual heterogeneity. Aerial and satellite images of the same location can differ dramatically due to variations in time of day, season, and weather conditions, while even objects within a single class (e.g. buildings) exhibit substantial diversity in size, shape, height, function and lighting conditions.

A recent paper[1] introduces the “dense labeling remote sensing dataset” (DLRSD) for advanced remote sensing tasks, including multi-label classification, image retrieval, and pixel-based applications like semantic segmentation. This dataset is a densely labeled version of the UC Merced Land Use Dataset[2], a widely-used benchmark for image-level land use classification, whereby individual pixels of each image are labeled with 17 class labels.

We prompted ERA to train a model to classify pixels into the land cover classes and provided a pre-specified, reproducible 80/20 train/test split of imagery in the DLRSD dataset. For each experiment, we validated model performance on the held out test set of 420 randomly selected images using a standard “mean intersection over union” (mIoU) metric.

The three top performing solutions generated by tree search significantly outperformed reported results in recent academic papers on the DLRSD benchmark, achieving mIoU greater than 0.80 (Supplementary Table S14, Supplementary Fig. S14). All three solutions build upon existing models, libraries and strategies. Solutions 1 and 3 leverage standard UNet++ and U-Net models but paired with powerful encoders (efficientnet-b7 and se-resnext101-32x4d) pre-trained on ImageNet[3]. Solution 2 uses SegFormer, a state of the art Transformer-based architecture. Key differentiators among the models included their data augmentation and prediction strategies. The U-Net++ and U-Net models leveraged extensive augmentation from the Albumentations library, whereas the Segformer model used a more basic set of transforms. All three solutions employ extensive Test-Time Augmentation (TTA)[4] by predicting masks for multiple augmented versions of a single test image (e.g., horizontal flips, vertical flips, rotations) which are then reverse-transformed and averaged to produce a final, more robust mask which smooths out prediction errors and boosts performance. A representative tree and breakthrough plot for Solution 3 is shown in Supplementary Fig. S15.

1.2. Neuroscience: Whole-Brain Neural Activity Prediction

We consider the Zebrafish Activity Prediction Benchmark (ZAPBench), a recent dataset designed to test predictions of cellular-resolution neural activity in an entire vertebrate brain[5]. The benchmark uses a novel dataset capturing brain activity of a larval zebrafish over a two-hour session using light-sheet fluorescent microscopy, resulting in 3D brain volumes recorded over time. Throughout the recording, the animal was exposed to distinct visual stimulus conditions designed to elicit a range of different behaviors. The raw volumetric video data was extensively processed to align, motion-stabilize, and segment into activity traces, resulting in a final data matrix of activity traces for 71,721 neurons across 7,879 time steps.

Several state-of-the-art forecasting methods were evaluated on the benchmark[5], including time-series forecasting methods that operate on the extracted activity traces per neuron, as well as a volumetric video prediction model (a Unet variant) that directly processes the 3D brain volumes

over time[6]. The video-based approach exploits spatial information that is lost when converting the data to time series, but is computationally expensive. Among the different methods evaluated on the benchmark, the video-based Unet model achieved the best overall performance, especially in the setting where only a short window of past context is available.

We prompted ERA to solve the multivariate time-series forecasting problem, predicting the output activity of all neurons for up to 32 time steps ahead in the time-series domain, given their past 4 time steps of activity as context, using the dataset splits provided by ZAPBench[5] which split each stimulus condition into 70% for training, 10% for validation, and 20% for testing per stimulus condition. We used the validation set for model selection, including hyperparameter tuning and early stopping, and to obtain a score to guide the tree search. We score solutions using mean absolute error (MAE) averaged across the prediction horizon, and compare solutions found by tree search against the methods included in ZAPBench: These include a linear model[7], TiDE[8], TSMixer[9], Time-Mix (a variant of TSMixer where feature mixing is ablated), and a custom Unet architecture[6].

Our initial experiment using tree search led to a best-performing model that uses a rich feature set from the input window, combining temporal convolutions, a learned "global brain state", and neuron-specific embeddings. The model then processes these features through a series of weight-shared residual blocks and a final dense layer to generate the multi-step prediction in one shot. Supplementary Fig. S16 shows the result of this model, compared to other baselines. In that figure, the mean baseline predicts the average over the context window, while the stimulus baseline predicts the average for each stimulus phase. Remarkably, the model produced by tree search outperformed all other baselines, including the best-performing video model, except for 1-step-ahead predictions. A representative example of the breakthrough plot and tree is shown in Supplementary Fig. S17.

We then developed a separate model tuned specifically for 1-step-ahead predictions with another tree search. The resulting solution is conceptually similar to the first in that both architectures generate a learned global context vector to inform their per-feature predictions. However, this model computes its global context using a dynamic attention mechanism for weighted aggregation and modulates feature representations through a FiLM-like layer[10] for interactive conditioning. This model achieved leading performance on 1-step-ahead predictions (Supplementary Fig. S16).

Both of these solutions are orders of magnitude faster to train than the best-performing video model–less than two hours on a single T4 GPU, as compared to 36 hours on 16 A100 GPUs for the Unet model. In addition, our solutions effectively use cross-neuron information to generate predictions, a major challenge highlighted in previous work[5].

A key future direction is the development of models that incorporate biophysical information and are more interpretable. The forthcoming synaptic-level structural reconstruction of the larval zebrafish brain used for ZAPBench provides a unique opportunity to develop such models by integrating anatomical wiring diagrams. As an initial exploratory step, we prompted ERA to use Jaxley[11], a JAX-based library for differentiable simulation of biophysically detailed neuron models, for the tree search. The resulting best-performing solution simulates each neuron independently using single-compartment Hodgkin-Huxley models[12]. Crucially, it dynamically modulates each neuron's biophysical parameters based on its recent activity history. To account for inter-neuronal interactions without the computational cost of direct synaptic simulation, the model then processes the outputs of these independent simulations through a latent autoencoder. This learns a system-wide corrective signal, effectively modeling a *functional* connectome–a reasonable hybrid approach in the absence of the structural connectome. While this model did not outperform the top-performing video model, it was competitive with time-series baselines (Supplementary Fig. S16).

1.3. Numerical Analysis: Library for Numerical Evaluation of Difficult Integrals

We applied ERA to the problem of numerical evaluation of difficult integrals using Gaussian quadratures. The gold standard, QUADPACK[13], was developed in the 1980s and underlies the popular Python function `scipy.integrate.quad()`. However, this function can fail in multiple ways, among them: the underlying algorithm can fail to converge; the algorithm samples its integrand, and the sampling may miss important features; the algorithm loses precision when the problem exhibits precise cancellations.

While standard techniques exist to address these problems, we asked whether ERA could build a general-purpose method superior to `quad()`, by hill-climbing on a benchmark set of integrals for which the standard algorithm fails but the analytic solution is known. Our dataset consists of 38 oscillatory integrals with infinite upper limits and without other pathologies, for which `quad()` returned an incorrect answer (Supplementary Fig. S18).

To build this list of integrals, we started with a long list of integrals in LaTeX form from Gradshteyn and Ryzhik[14]. We converted both the question and solution into a python expression using SymPy[15]. Most expressions included free parameters, often with value constraints. To enable numeric evaluations, we generated random values for all parameters consistent with the constraints.

Once an integral and its answer were in the form of SymPy expression objects, we evaluated answers numerically by substituting our chosen parameter values using `sympy.Expr.subs()` and evaluating via `sympy.evalf()`. We build integrand functions suitable for `scipy.integrate.quad()` via `sympy.lambdify` for efficient evaluation. We compared each numerical answer to the number returned by `scipy.integrate.quad()` and discarded cases where the numbers agreed within the latter's error estimate. We also discarded cases where that error estimate was greater than 2% of the numbers' magnitude.

All conversions from LaTeX to SymPy and all constrained parameter generations were performed by Gemini using specialized prompts. The resulting SymPy expressions and parameter values were examined manually for correctness. These manual steps were the limiting factor on the scale of our dataset.

We randomly split these into training and evaluation datasets (n=19 integrals each). We initialized ERA with a simple invocation of `quad()` and prompted the system to improve it on a tree search over 1000 nodes. We scored solutions with the logarithm of the absolute fractional error (discrepancy between the generated solution's number and the answer's number), where the logarithm prevented the search from over-weighting outliers.

$$\text{score} = -\log\left(1 \left|\frac{\text{response} - \text{answer}}{\text{answer}}\right|\right) \tag{1}$$

The resulting breakthrough plot and tree structure for the search are shown in Supplementary Fig. S19.

The best solution builds on `quad()` by partitioning the infinite domain into a sequence of contiguous, finite subintervals whose lengths may increase geometrically to cover the domain's tail more efficiently. The definite integral is thus transformed into an infinite series, where each term is the numerical integral of the integrand over one of these finite segments, calculated using `quad()`. For integrals that converge slowly, such as those with oscillatory integrands, direct summation of this series is impractical. The algorithm therefore applies Euler's transformation, a powerful series acceleration technique, to this sequence of segment integrals. By repeatedly averaging adjacent terms, the transformation extrapolates the limit of the slowly converging series from a finite number

of its initial terms, providing an accurate estimate of the integral's true value. The evolved code correctly evaluated 17 out of 19 held-out integrals to within a fractional error of less than 3 percent (Supplementary Fig. S20).

The evolved code always applies `quad()` first. It only falls back to its more specialized methods if `quad()` returns a large error estimate, returns `NaN` or `Inf`, or raises an exception. This means the evolved code is as accurate as `quad()` in less pathological cases and so could reasonably be used as a drop-in replacement.

1.4. Towards Genuine Discovery

Several recent deployments of our core tree-search algorithm demonstrate the potential for genuine discovery:

- **Mathematical Discovery** Our system was recently used to derive the exact analytical power spectrum of gravitational radiation emitted by cosmic strings[16]. Operating without empirical data or predictive modeling, the system's search navigated the space of symbolic mathematics to discover six novel analytical derivations to an integral that had not been previously solved, yielding a closed-form exact solution.
- **Neural Mechanisms:** In computational neuroscience, the system was tasked with discovering transition models of neural activity in an *in silico* larval zebrafish[17]. While unconstrained search yielded predictive models relying on statistical shortcuts, providing the system with structural priors (wiring diagrams) allowed the algorithm to autonomously discover the true, underlying mechanisms governing the neural circuit, successfully recovering causal effective connectivity.

Within predictive modeling tasks, the scoring schemes are by construction standardized and straightforward to implement. Yet, when applying this automated framework to novel, open-ended scientific domains, inventing an appropriate scoring metric requires significant domain expertise and scientific creativity. We illustrate how this can work for mathematical discovery[16], with the objective of discovering a closed-form analytical equation for the radiation spectra of cosmic strings. Our scoring metric measured how closely a proposed analytical solution matches a high-precision numerical solution: the system evaluated the AI-proposed analytical formulas at random physical parameter values and computed the residual difference against the numerical integration. Minimizing this difference lets the search discover six different analytical solutions and also a novel asymptotic expansion. In general, the design of metrics for complex scientific problems is an iterative endeavor. The scientific process implicitly includes creating scoring functions to try to reach a hypothesized goal, iterating when the goal is not reached. Within ERA, this requires that the user iterates the scoring function.

1.5. Computational Cost and Resource Utilization

Since search-based LLM optimization relies on iterative generation and empirical evaluation, it is inherently more resource-intensive than single-shot or zero-shot prompting. Here we detail representative computational costs required per search node for representative search runs across our evaluated benchmarks in Supplementary Table S1.

2. Supplementary Figures

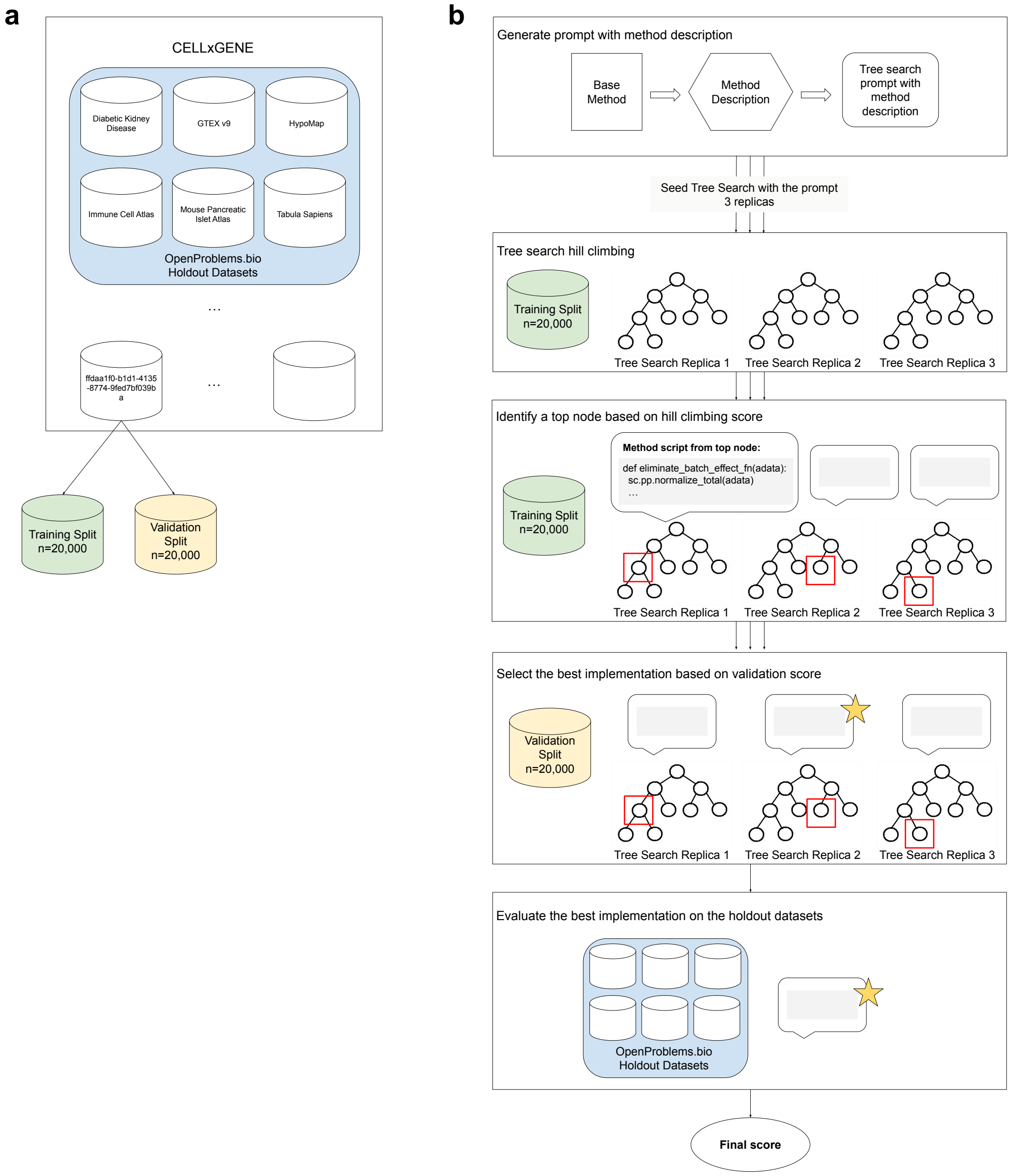

Supplementary Fig. S1 | Experimental design for single-cell batch integration. a, We sourced our tree search development dataset from CELLxGENE. After filtering and manually selecting the dataset `364bd0c7-f7fd-48ed-99c1-ae26872b1042` version `ffdaa1f0-b1d1-4135-8774-9fed7bf039ba` (see Methods), which has a similar profile to the six datasets used in the OpenProblems.bio Batch Integration benchmark (distinct datasets also in CELLxGENE), we sampled 20,000 cells for the training split and 20,000 for the validation split. **b,** For each of the 11 base methods, we generated a detailed method description and inserted it into a prompt to initialize the tree search. We ran three independent tree search replicas per method, using the training split for hill climbing. From each tree, we selected the top-performing node based on its training score. We then evaluated each top node's script on the validation split and selected the best one based on validation performance. The best implementation per method was finally evaluated on the OpenProblems.bio holdout datasets, and the corresponding scores are reported as final results.

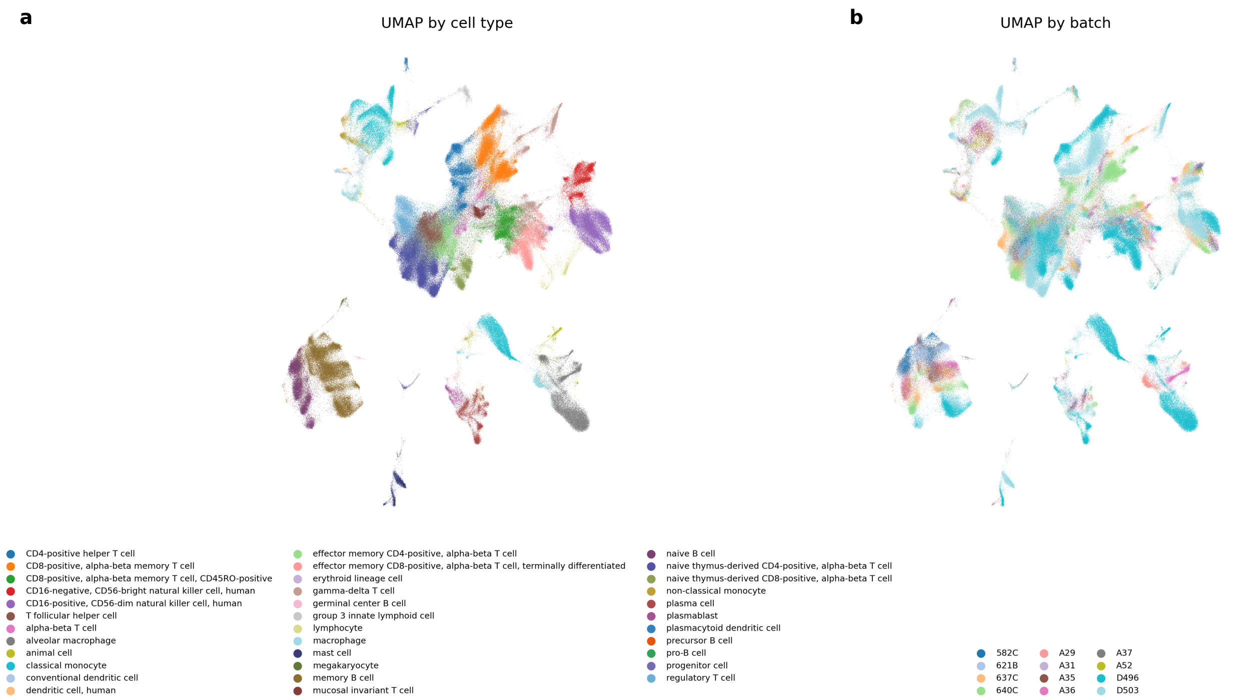

Supplementary Fig. S2 | Uniform Manifold Approximation and Projection[18] of `BBKNN (TS)` on the Immune Cell Atlas dataset. a, The UMAP projection colored by cell type shows cell-type-specific clusters. **b,** The UMAP projection colored by data batch shows good batch mixing across the dataset.

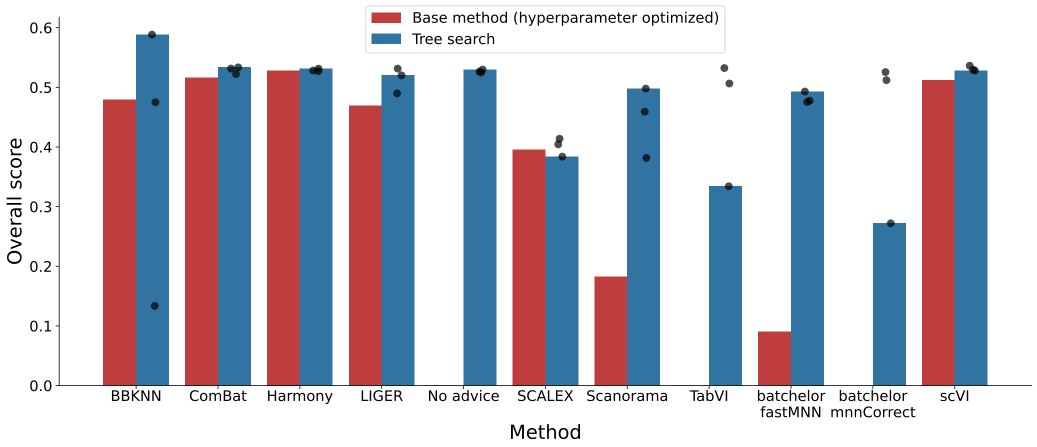

Supplementary Fig. S3 | Relative performance of base methods with optimized hyperparameters and ERA replicates. Overall scores on the holdout OpenProblems datasets for all replicates of methods evaluated in Fig. 2. Hyperparameters for the base methods were optimized using the training dataset. For tree search implementations, three replicates of the full process were performed. Dots indicate the overall score of the replicate on the holdout OpenProblems datasets. The bar shows the performance of the replicate with highest performance in the validation dataset (identical values to those shown in Fig. 2). The `No advice` and `TabVI` methods have no base method code available. The `batchelor mnnCorrect` hyperparameter-optimized base method code failed to compute embeddings on every OpenProblems dataset owing to out-of-memory errors.

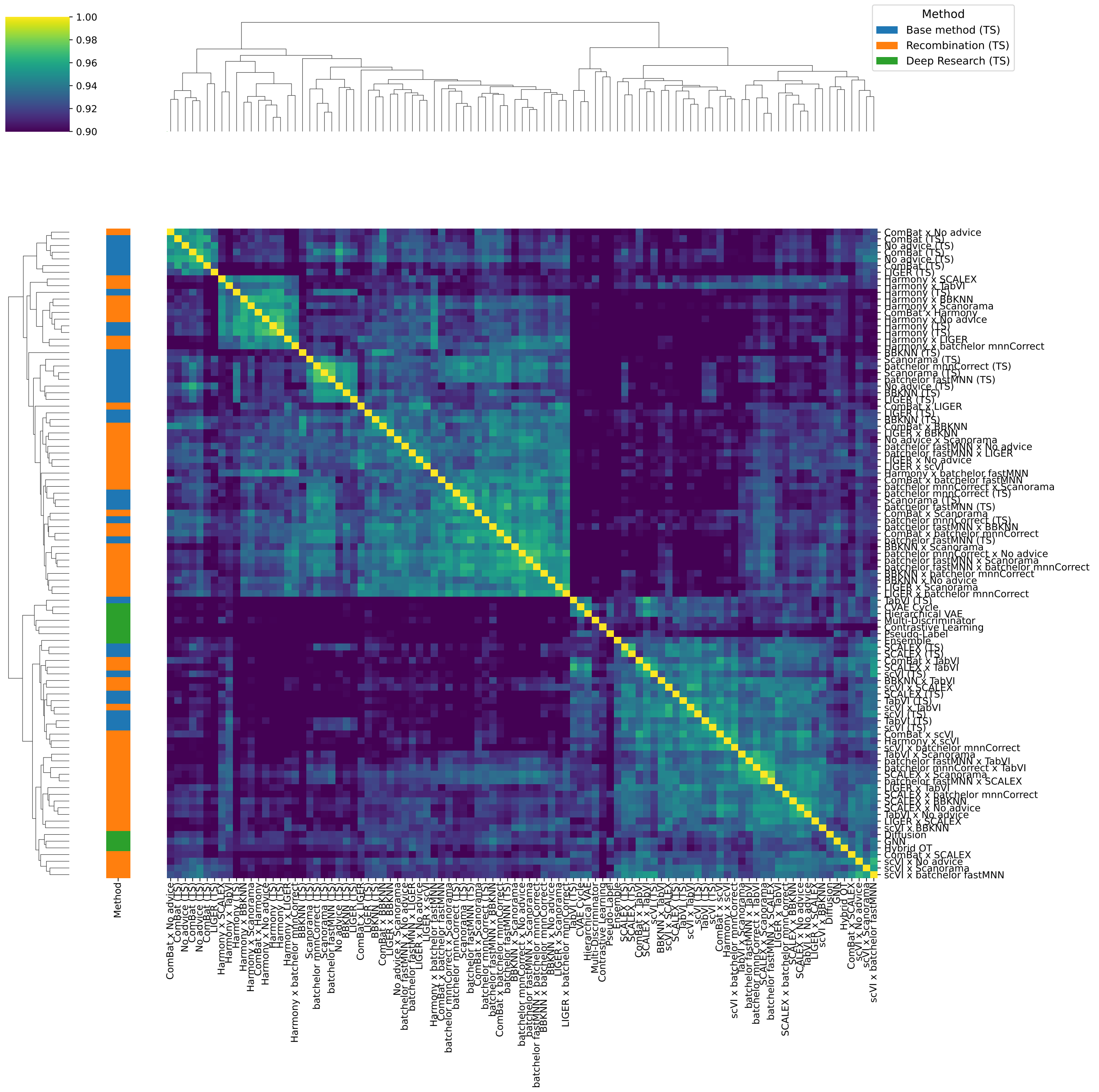

Supplementary Fig. S4 | Heatmap of text embedding cosine similarities among ERA-generated methods. The similarity matrix was hierarchically clustered along rows and columns and reordered to group similar methods together. Three distinct color bars denote major method categories. The pairwise cosine similarities between tree search-generated solutions were greater than 0.85. For context, the lower bound of cosine similarity, established by averaging the similarities between GIFT-Eval's methods (a completely different benchmark) and batch integration methods, was 0.74.

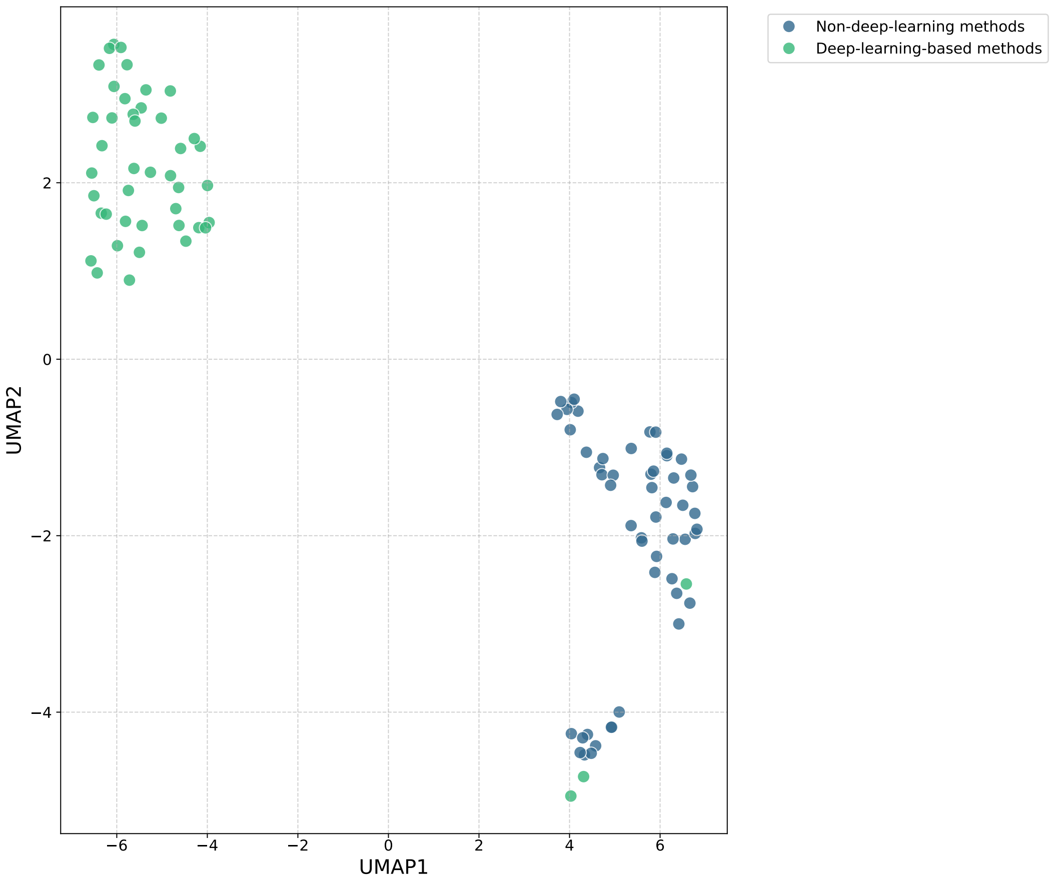

Supplementary Fig. S5 | UMAP of text embeddings representing ERA-generated methods for single-cell batch integration. The UMAP shows two major clusters: non-deep-learning methods (blue) and deep-learning-based methods (green). We confirmed this clustering by using Gemini to classify the code associated with each method.

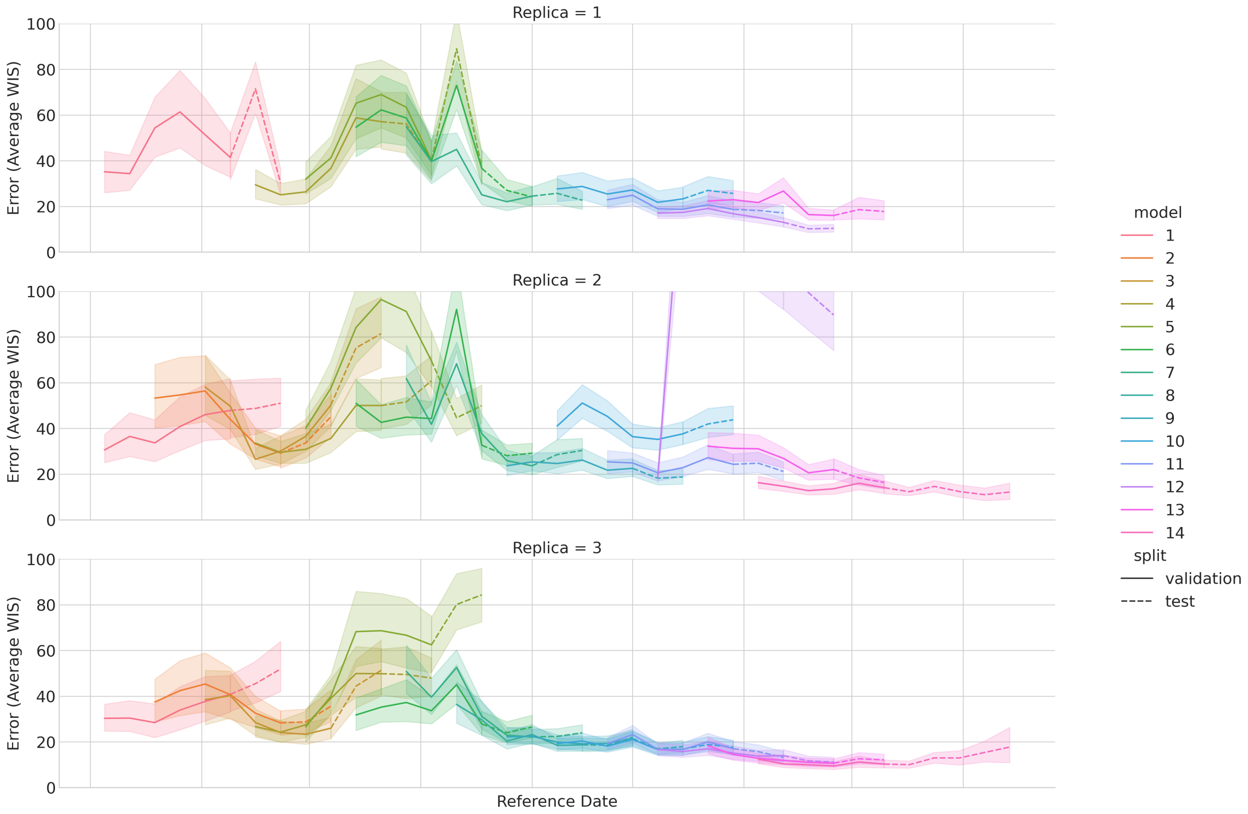

Supplementary Fig. S6 | Performance of retrospective COVID-19 hospitalization forecasts across all replicates. Each panel displays the average WIS by reference date for individual replicates of our proposed models, for all rolling validation dates. Lower WIS values indicate superior forecasting accuracy and calibration. The consistent trends across replicates demonstrate the robustness and reproducibility of tree search's ability to generate high-performing probabilistic forecasts.

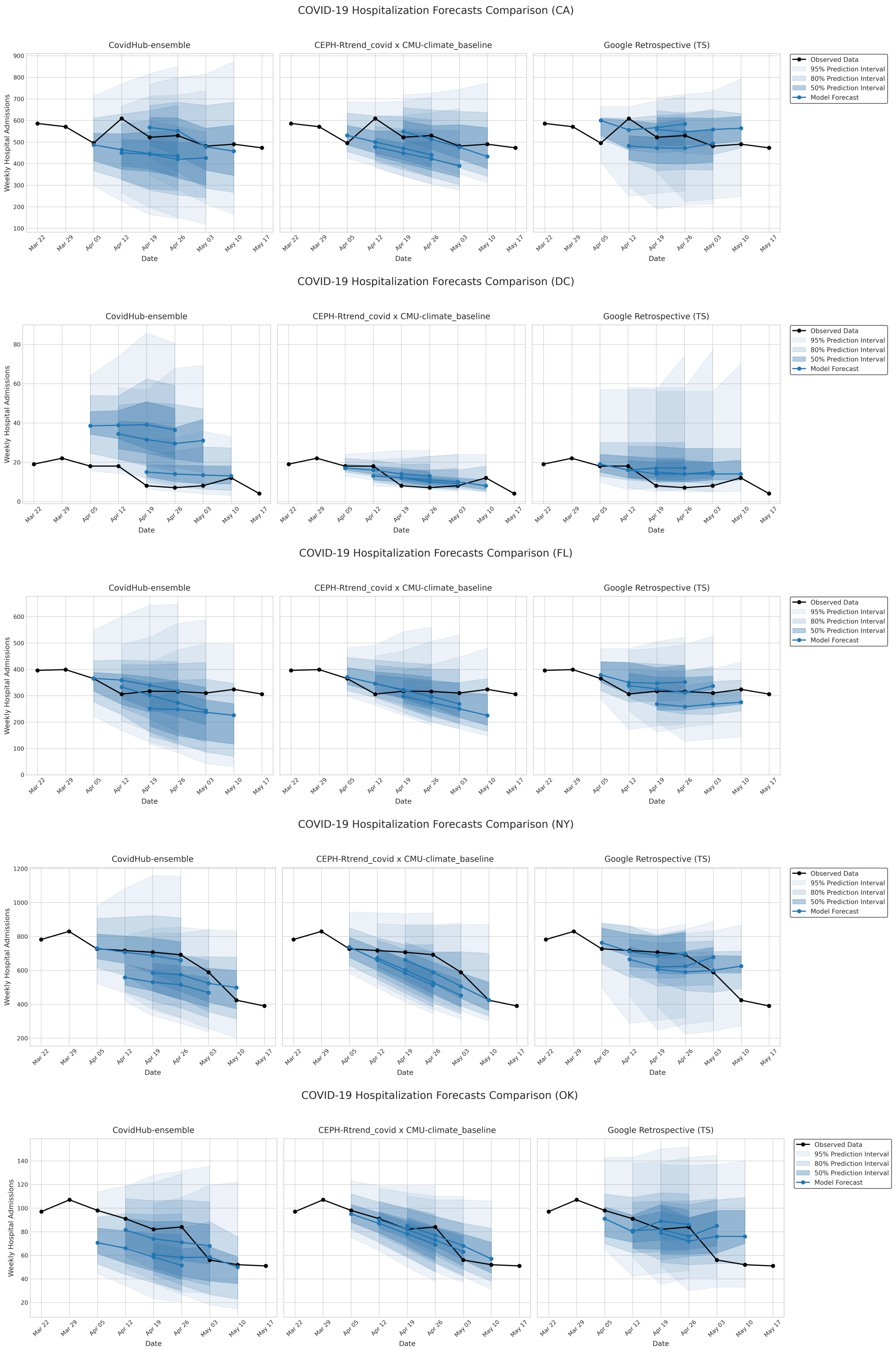

Supplementary Fig. S7 | Visual validation of COVID-19 hospitalization forecasts. Comparison of ground truth (Observed Data) against the CovidHub-ensemble, the lowest WIS recombination solution (CEPH-Rtrend_covid × CMU-climate_baseline), and the Google Retrospective (TS) across five representative jurisdictions (CA, DC, FL, NY, OK). The ERA-developed models demonstrate a visible qualitative improvement in tracking non-linear dynamics and providing better-calibrated prediction intervals. This comparison confirms that the numerical improvements in WIS are driven by superior predictive accuracy across diverse temporal regimes, rather than statistical artifacts or a bias toward low-constant values.

Supplementary Fig. S8 | Full-season COVID-19 forecasts across all validation splits. Comparison between the CovidHub-ensemble (left) and the Google Retrospective (TS) model (right). The overlapping colored ribbons represent successive 6-week validation splits throughout the 2024-2025 season.

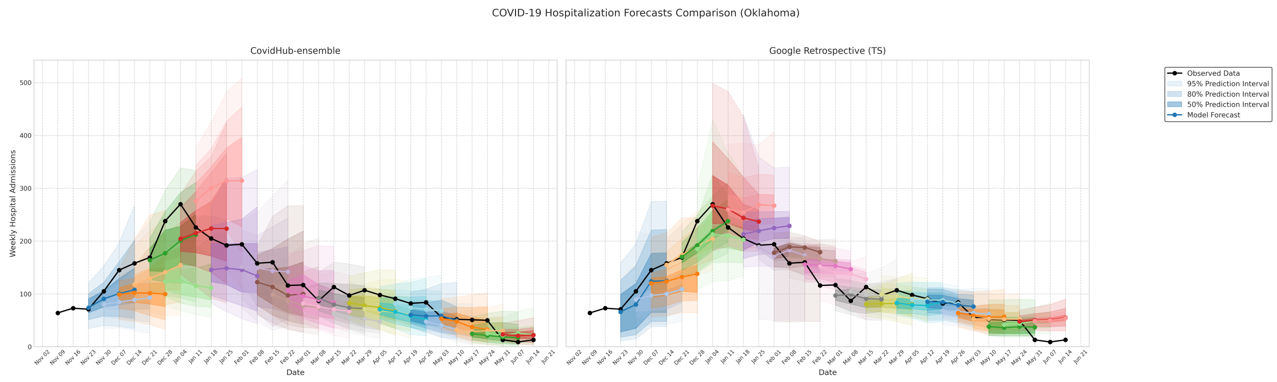

(a) Oklahoma

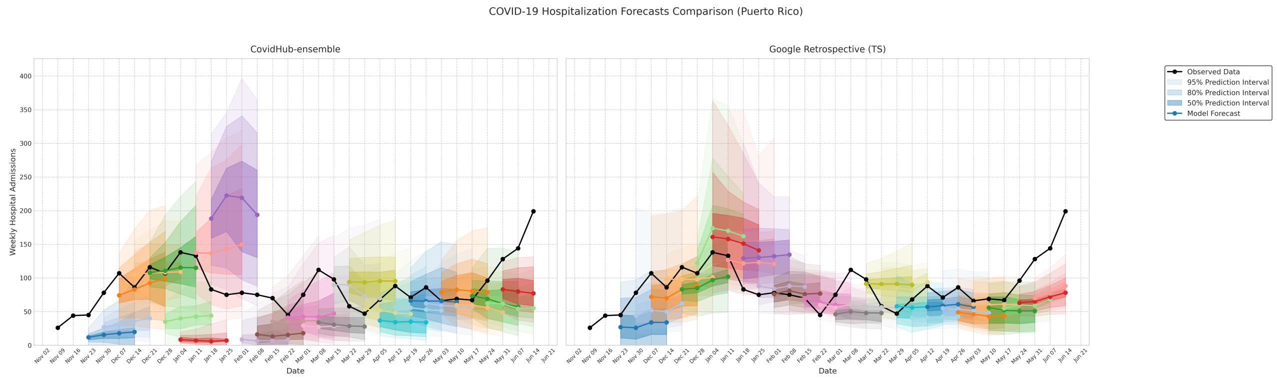

(b) Puerto Rico

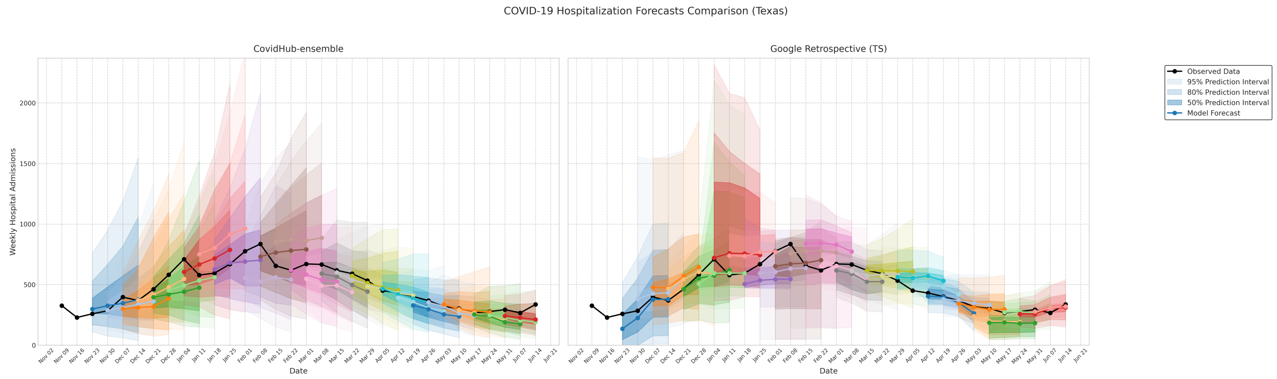

(c) Texas

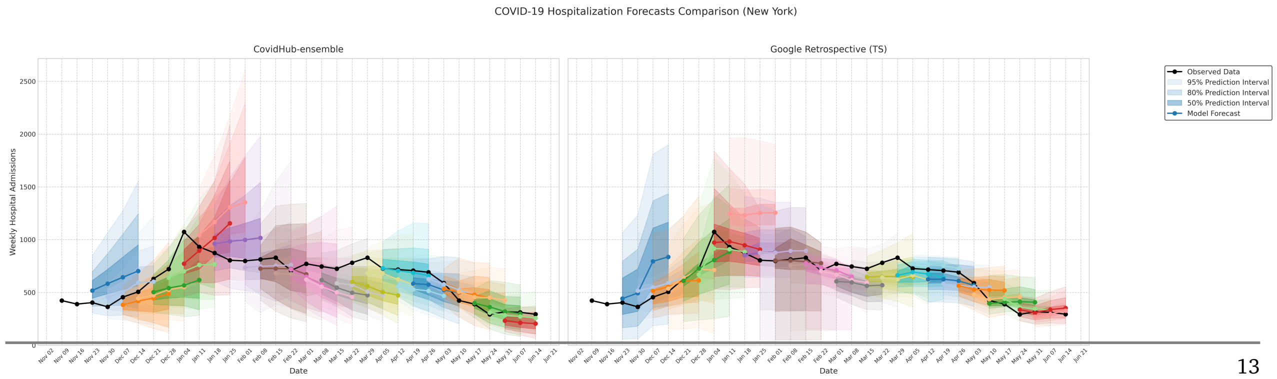

(d) New York

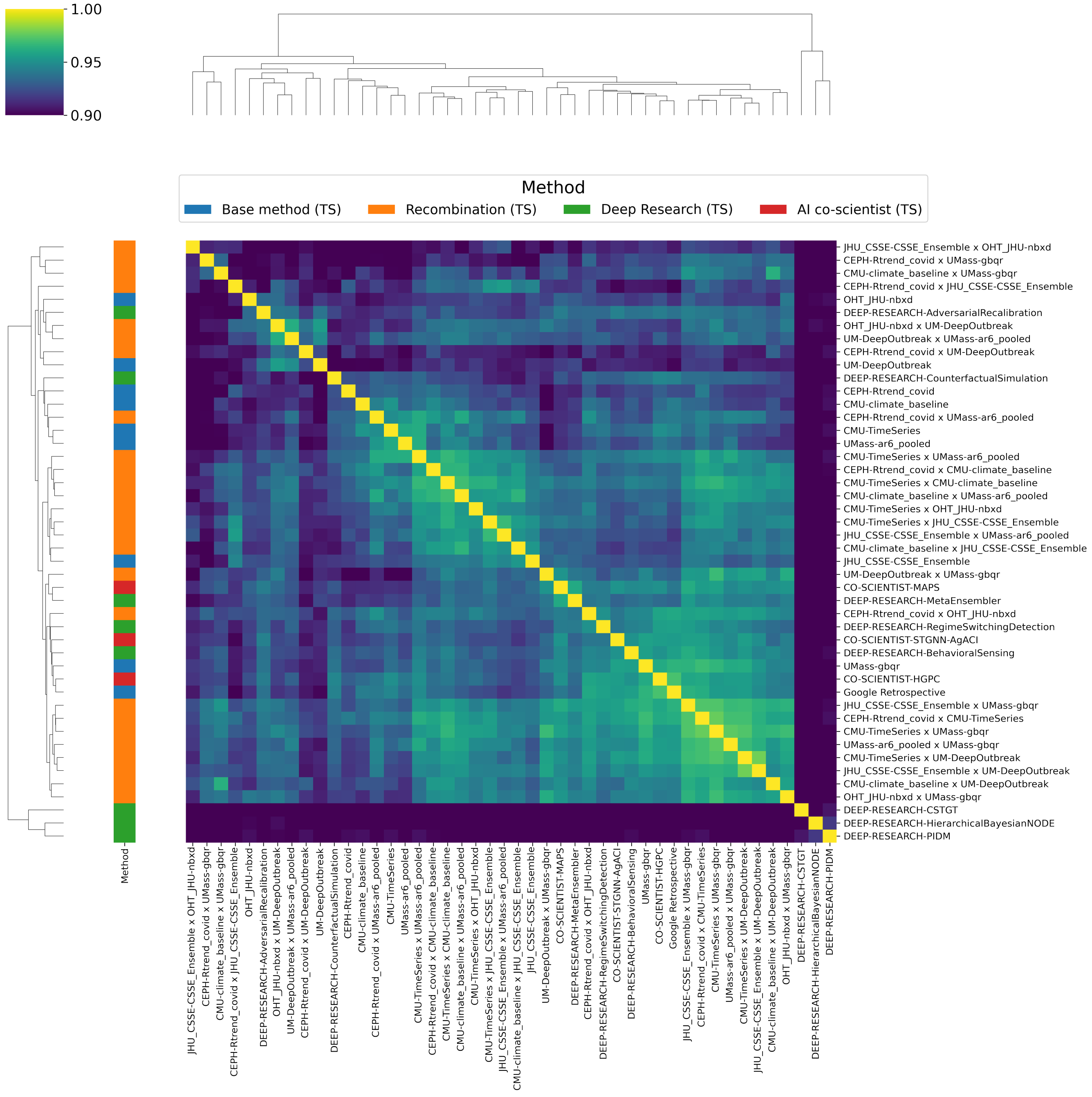

Supplementary Fig. S9 | Heatmap of conceptual similarities among COVID-19 forecasting generated codes for methods. This figure displays the pairwise cosine similarities between text embeddings of all forecasting models generated by tree search for the COVID-19 prediction task. Text embeddings were produced using a Gemini model[19]. The similarity matrix was then hierarchically clustered and reordered to group conceptually related strategies. The color-coded sidebar categorizes each method by its origin illustrating the composition of the emergent conceptual clusters. The No Advice methods are from the Google Retrospective study.

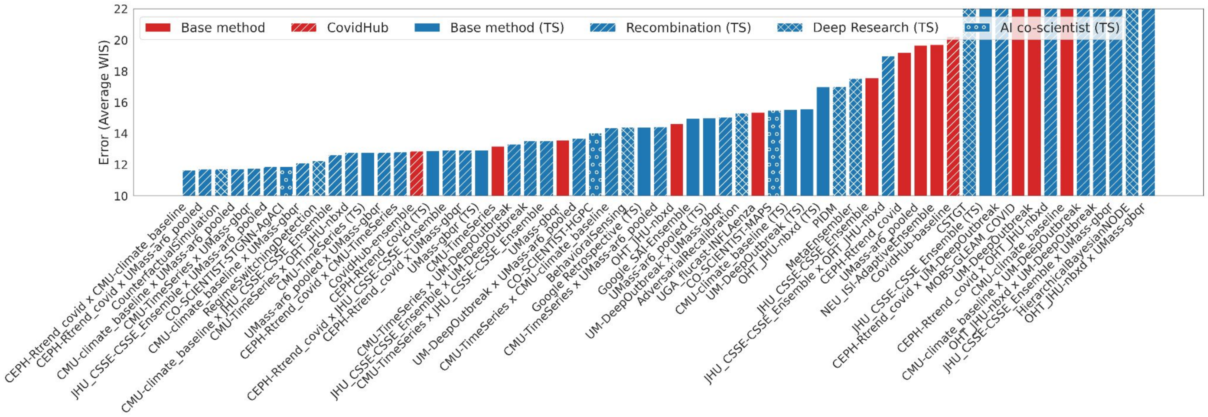

Supplementary Fig. S10 | Performance of recombination experiments for COVID-19 forecasting. Average WIS across all evaluated models on the common held-out test set. This extended plot highlights the full distribution of search outcomes, including the 9 Tree Search generated models and 3 external models that performed worse than the CovidHub-baseline (indicated by red bars). Models are color-coded by strategy: base methods, CovidHub submissions, and Tree Search optimized variants (Base method, Recombination, Deep Research, and AI co-scientist).

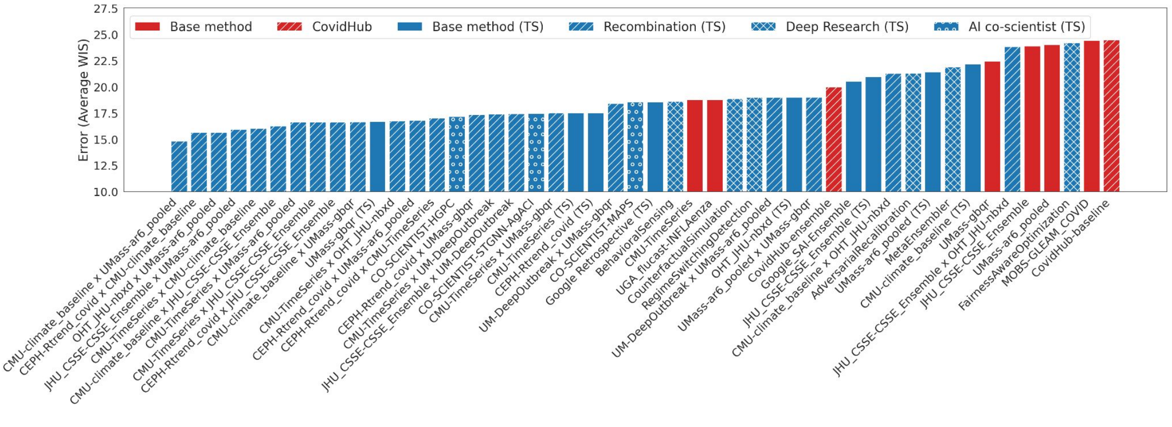

Supplementary Fig. S11 | Validation performance for COVID-19 forecasting. Average WIS for models evaluated on the six-week rolling validation window covering the reference dates 2025-02-22 to 2025-03-29. Performance on the validation dates correlates with the held-out test set results; for instance, the best-performing test model (CEPH-Rtrend_covid x CMU-climate_baseline) was identified as the second-best performer during this validation phase.

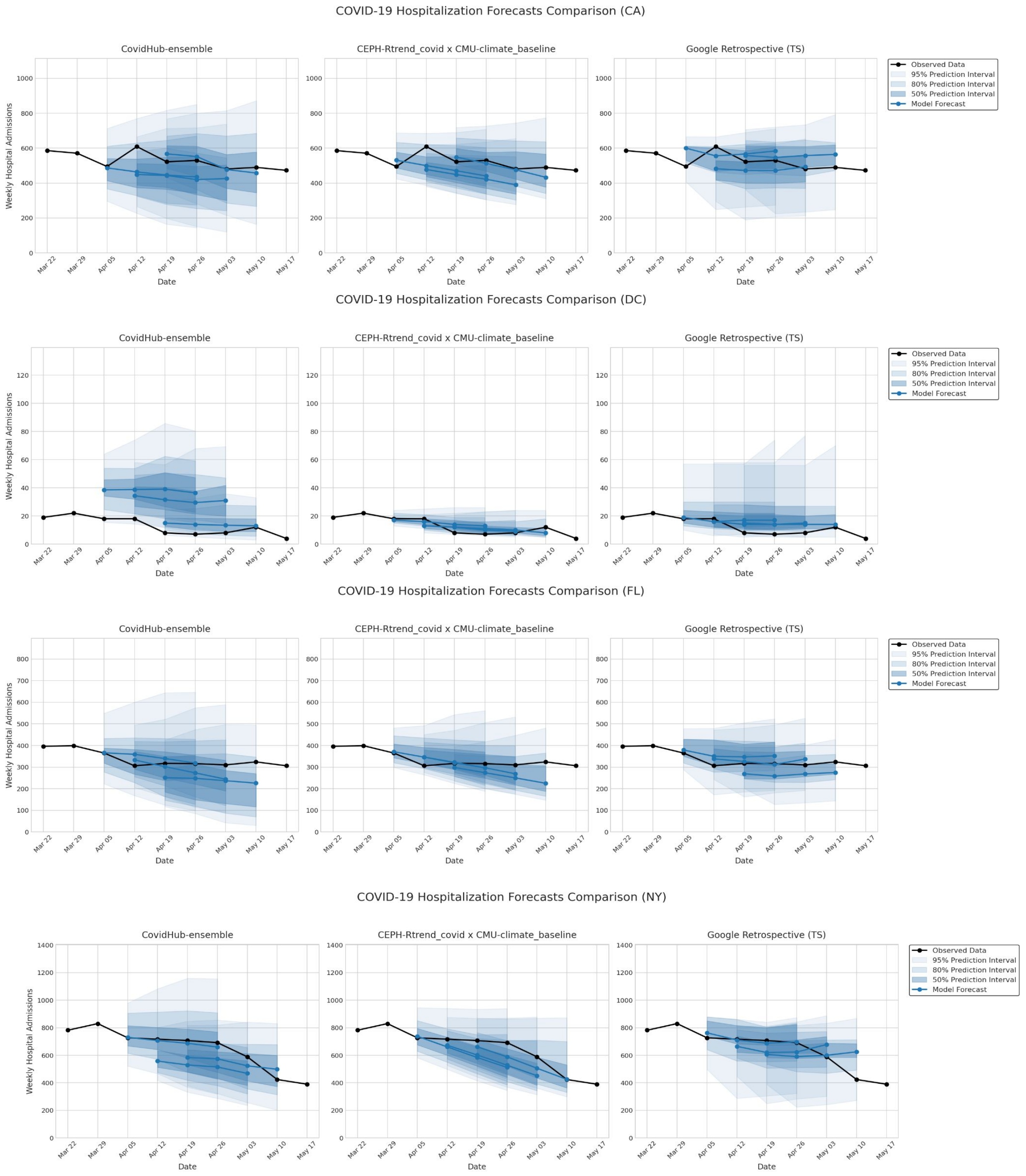

Supplementary Fig. S12 | Examples of COVID-19 forecasts. Observed ground truth data (black line) plotted against predictive models across four major jurisdictions (CA, DC, FL, and NY) for the reference dates 2025-04-05, 2025-04-12, and 2025-04-19. These dates are used as the held out test set in the analysis in the paper. Columns represent the CovidHub-ensemble (left), the best-performing tree search (TS) discovered recombination model (CEPH-Rtrend_covid x CMU-climate_baseline, middle), and the Google Retrospective TS model (right). The average WIS for this period are 11.63 for the top recombination model, 12.85 for the CovidHub-ensemble, and 14.39 for the Google Retrospective model. The TS methods generate significantly narrower confidence bands—indicating higher predictive precision—while consistently containing the ground truth data.

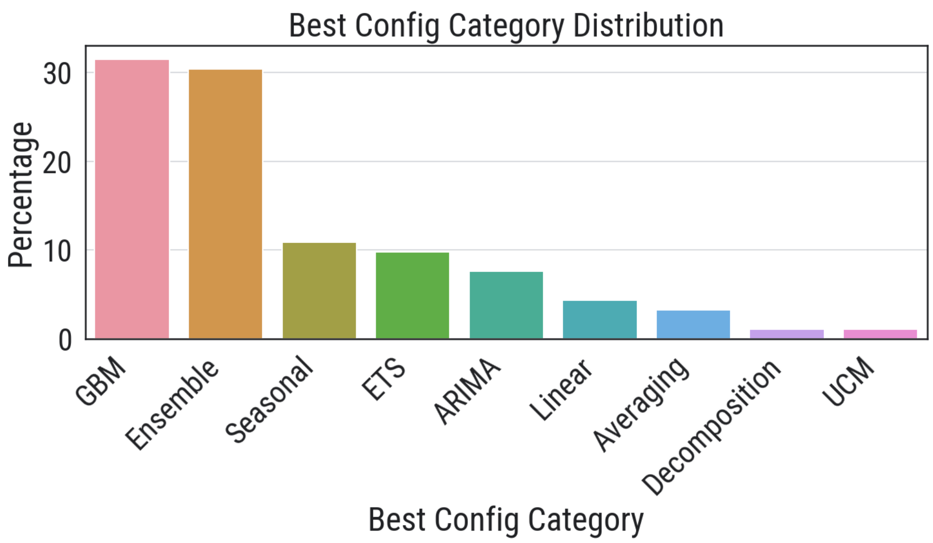

Supplementary Fig. S13 | Categories of solutions on the GIFT-Eval benchmark on the `per-dataset solution (v1)`. We prompted an LLM (Gemini 2.5 Pro) to categorize the code from each of the solutions into a class of methods. The figure shows the percentage of the best codes for each of the 92 competitions in the specified categories: Gradient Boosted Method (GBM); Ensemble; Seasonal; Error, Trend and Seasonality (ETS); Arima[20]; Linear; Averaging; Decomposition and Unobserved components model (UCM).

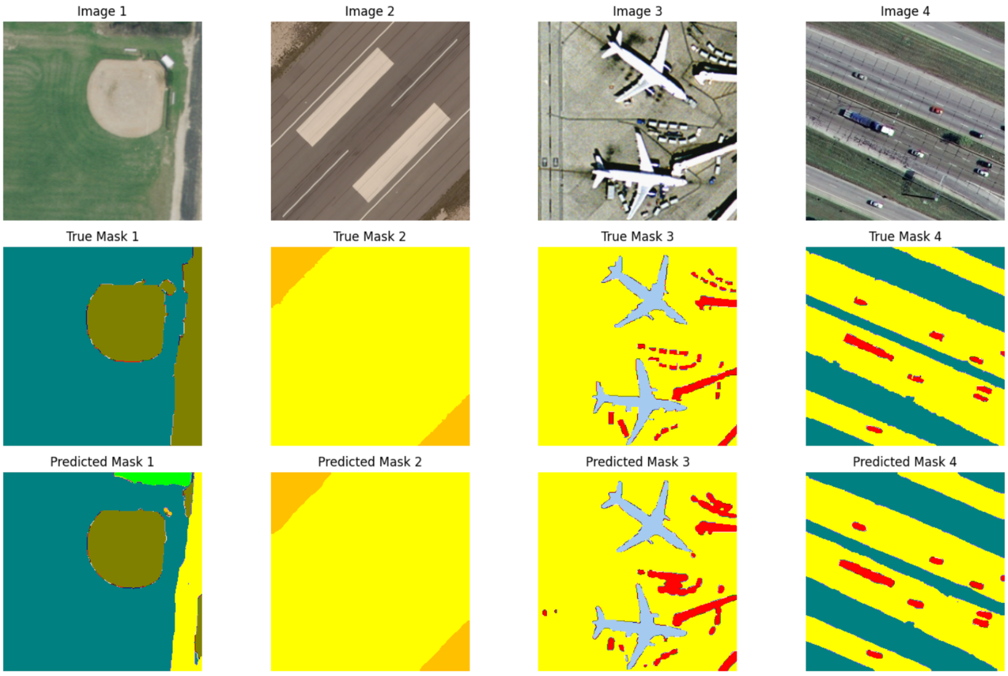

Supplementary Fig. S14 | Example output segmenting DLRSD image pixels from ERA Solution 1 (U-Net++).

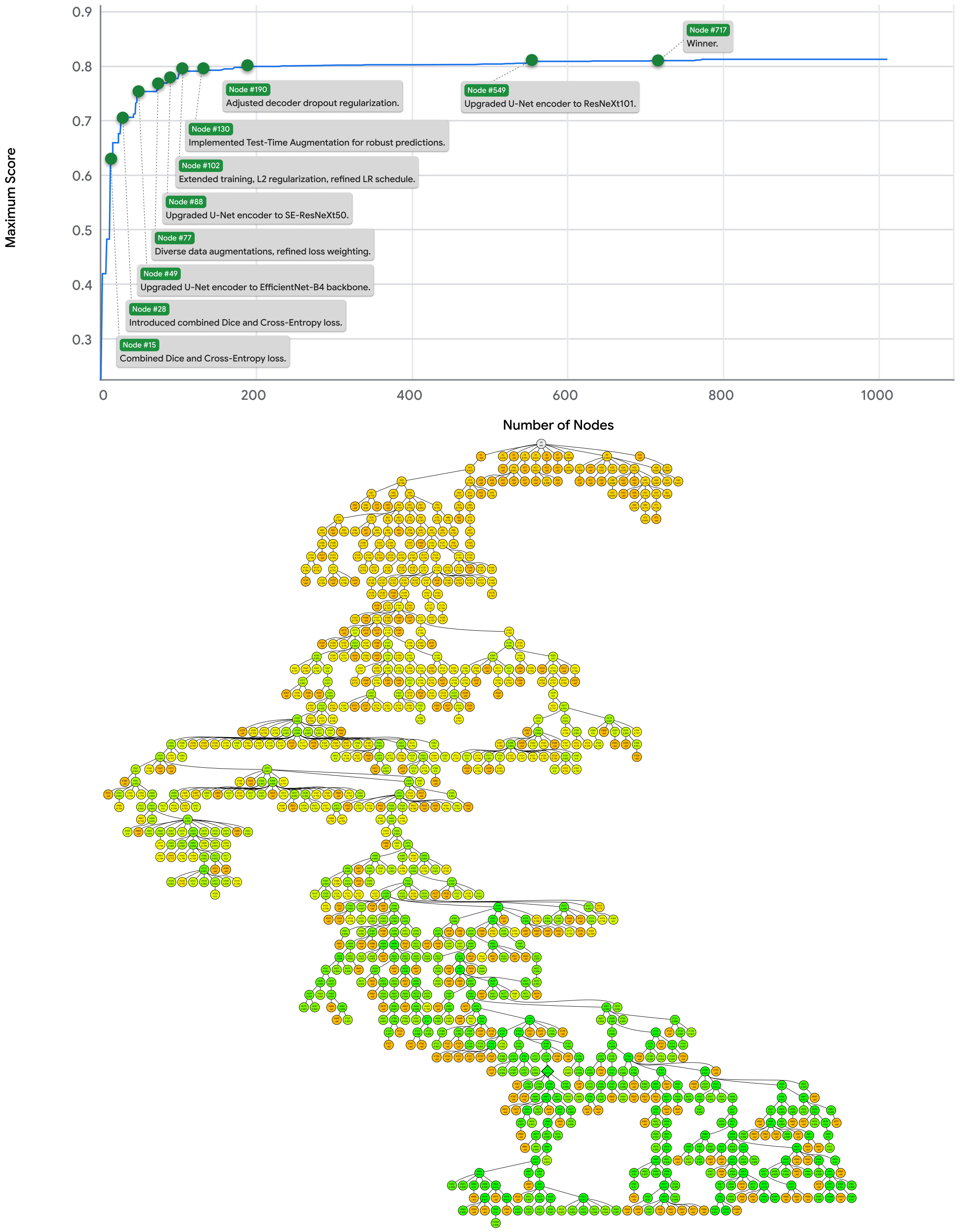

Supplementary Fig. S15 | Breakthrough plot and solution tree for the geospatial segmentation task. *Top Figure* Breakthrough plot for the U-Net Geospatial DLRSD solution (solution 3), showing the evolution of the maximum score as a function of the number of nodes. The green dots label places where the score abruptly increases due to an improvement in the code, and the label describes the change in the code that resulted in the score increase. *Bottom Figure* Structure of the tree for this same search. The color range consists of orange (lower scores) to green (higher scores) with the highest score denoted by a diamond node.

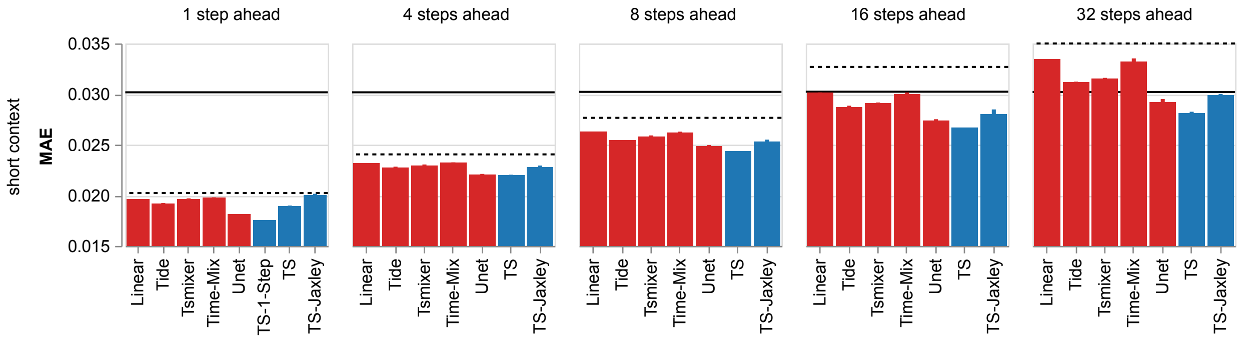

Supplementary Fig. S16 | Comparison of solutions to time-series and video forecasting methods across conditions on ZAPBench. Solutions are evaluated using average mean absolute error (MAE) across conditions (lower is better). For ERA, we report the performance of three different solutions (blue), and compare them against baselines (red). Alongside our best general solution (TS), we include results from two specialized runs: a tree search that was optimized for 1-step ahead forecasting as well as a solution prompted to use Jaxley, a differentiable biophysical neuron simulator. The dotted and solid lines represent the mean and stimulus baselines, respectively. To account for variability due to random number generator seeding, each method was run three times. We report the mean, with error bars indicating 95% confidence intervals.

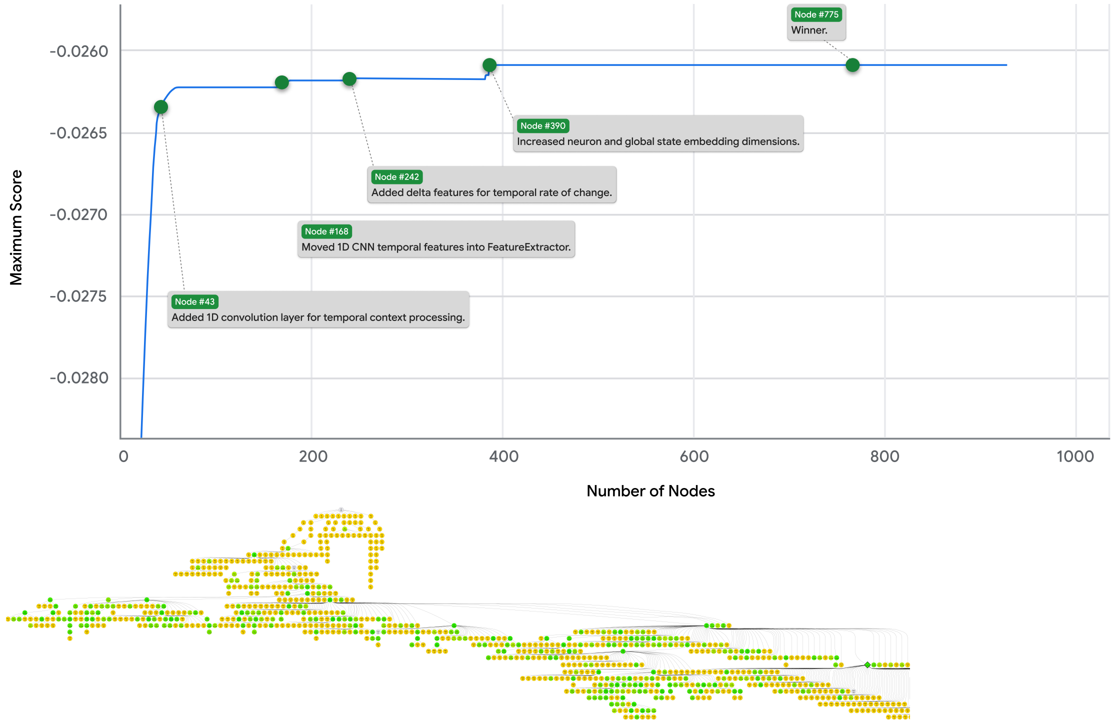

Supplementary Fig. S17 | Breakthrough plot and solution tree for the ZAPBench task. *Top Figure* Breakthrough plot for the ZAPBench task, showing the evolution of the maximum score as a function of the number of nodes. The green dots label places where the score abruptly increases due to an improvement in the code, and the label describes the change in the code that resulted in the score increase. *Bottom Figure* Structure of the tree for this same search. The color range consists of orange (lower scores) to green (higher scores) with the highest score denoted by a diamond node.

train set		test set	
445.001	${}_{0}^{\infty} \sin\left(x^2\right) dx$	446.021	${}_{0}^{\infty} \left(\sin^4\left(ax^2\right) - \sin^4\left(bx^2\right)\right) dx$
445.017	${}_{0}^{\infty} \sin\left(ax^2\right)\cos\left(2bx\right) dx$	446.045	${}_{0}^{\infty} x\cos\left(ax^2\right)\cos\left(2bx\right) dx$
447.012	${}_{0}^{\infty} \sin\left(ax^2 \; \frac{b^2}{a}\right)\cos\left(2bx\right) dx$	449.013	${}_{0}^{\infty} x^{\mu-1}\sin\left(ax\right)\cos\left(bx\right) dx$
458.031	${}_{0}^{\infty} \left(\frac{\gamma x}{\beta^2\left(\gamma x\right)^2} - \frac{\gamma - x}{\beta^2\left(\gamma - x\right)^2}\right)\sin\left(ax\right) dx$	465.002	${}_{0}^{\infty} \frac{\left(3-4\sin^2\left(ax\right)\right)\sin^2\left(ax\right)}{x} dx$
462.034	${}_{0}^{\infty} \frac{x\sin\left(ax\right)\cos\left(bx\right)}{c^2x^2} dx$	465.013	${}_{0}^{\infty} \frac{\sin^{2m1}\left(x\right)\sin\left(x\left(6m3\right)\right)}{a^2x^2} dx$
477.049	${}_{0}^{\infty} \frac{x\sin\left(ax\right)\cos\left(ax\right)}{x^2 1} dx$	467.025	${}_{0}^{\infty} \frac{\sin\left(x\right)\cos\left(x\right)}{x\sqrt{\sin^2\left(x\right)1}} dx$
478.036	${}_{0}^{\infty} \frac{\left(\cos\left(a\right)-\cos\left(anx\right)\right)\sin\left(mx\right)}{x} dx$	478.031	${}_{0}^{\infty} \sin\left(ax^p\right) dx$
487.011	${}_{0}^{\infty} \frac{1}{x}\frac{\sin\left(x\right)}{\left(a^2\cos^2\left(x\right)b^2\sin^2\left(x\right)\right)^2} dx$	478.050	${}_{u}^{\infty} \frac{\cos\left(ax\right)}{\sqrt{-ux}} dx$
487.026	${}_{0}^{\infty} \frac{1}{x}\frac{\sin\left(x\right)\cos^2\left(x\right)}{\left(a^2\cos^2\left(x\right)b^2\sin^2\left(x\right)\right)^2} dx$	484.059	${}_{0}^{\infty} \left(\sin\left(a - x^2\right) \; \cos\left(a - x^2\right)\right) dx$
488.014	${}_{0}^{\infty} \frac{1}{x}\frac{\sin^3\left(x\right)\cos\left(x\right)}{\left(a^2\cos^2\left(2x\right)b^2\sin^2\left(2x\right)\right)^4} dx$	487.068	${}_{0}^{\infty} \frac{\cos\left(x\right)\cos\left(a\cos\left(x\right)\right)\cos\left(2nx\right)\sinh\left(a\sin\left(x\right)\right)}{x} dx$
491.004	${}_{0}^{\infty} \frac{\cos^{2m}\left(x\right)}{a^2x^2} dx$	494.006	${}_{0}^{\infty} x\sin\left(2bx\right)\cos\left(ax^2\right) dx$
491.006	${}_{0}^{\infty} \frac{\cos^{2m1}\left(x\right)}{a^2x^2} dx$	496.037	${}_{0}^{\infty} \frac{\sin^3\left(x\right)}{\left(a^2\cos^2\left(x\right)b^2\sin^2\left(x\right)\right)^3}\frac{1}{x} dx$
491.014	${}_{0}^{\infty} \frac{x\sin\left(2ax\right)\cos^2\left(bx\right)}{\beta^2x^2} dx$	504.025	${}_{0}^{\infty} \frac{\sin\left(ax^p\right)}{x} dx$
493.056	${}_{0}^{\infty} \frac{\sin\left(2ax\right)\cos^2\left(bx\right)}{x} dx$	504.061	${}_{0}^{\infty} \frac{\sin^3\left(x\right)\cos\left(x\right)}{x\sqrt{\sin^2\left(2x\right)1}} dx$
495.029	${}_{0}^{\infty} \frac{\sin^3\left(ax\right)\sin^2\left(bx\right)}{x} dx$	505.006	${}_{0}^{\infty} \frac{\sqrt{-b\sqrt{b^2x^2}}\sin\left(ax\right)}{\sqrt{b^2x^2}} dx$
504.057	${}_{0}^{\infty} \frac{\sin^3\left(x\right)\cos\left(x\right)}{x\sqrt{\cos^2\left(2x\right)1}} dx$	505.008	${}_{0}^{\infty} \frac{\sin\left(x\right)}{x\left(a^2\sin^2\left(x\right)b^2\cos^2\left(x\right)\right)} dx$
512.029	${}_{0}^{\infty} \frac{\cos\left(bx\right)\cos\left(p\sqrt{a^2x^2}\right)}{c^2x^2} dx$	505.023	${}_{0}^{\infty} \frac{\left(\cos\left(a\right)-\cos\left(anx\right)\right)\sin\left(mx\right)}{x} dx$
512.037	${}_{0}^{\infty} \frac{\cos\left(bx\right)\cos\left(p\sqrt{a^2x^2}\right)}{a^2x^2} dx$	513.033	${}_{0}^{\infty} \frac{\sin^3\left(ax\right)\cos\left(3bx\right)}{x^2} dx$
550.003	${}_{0}^{\infty} \frac{\sin\left(ax\right)\coth\left(\frac{\pi x}{2}\right)}{x^2 1} dx$	551.027	${}_{0}^{\infty} \frac{\sin^3\left(a^2x^2\right)}{x^2} dx$

Supplementary Fig. S18 | The dataset of 38 definite integrals with oscillatory integrands on semi-infinite domains[14], none of which were solved correctly by `scipy.integrate.quad()`. Parameters like a, b, c were chosen randomly between 0 and 5 with exponents constrained to be integers.

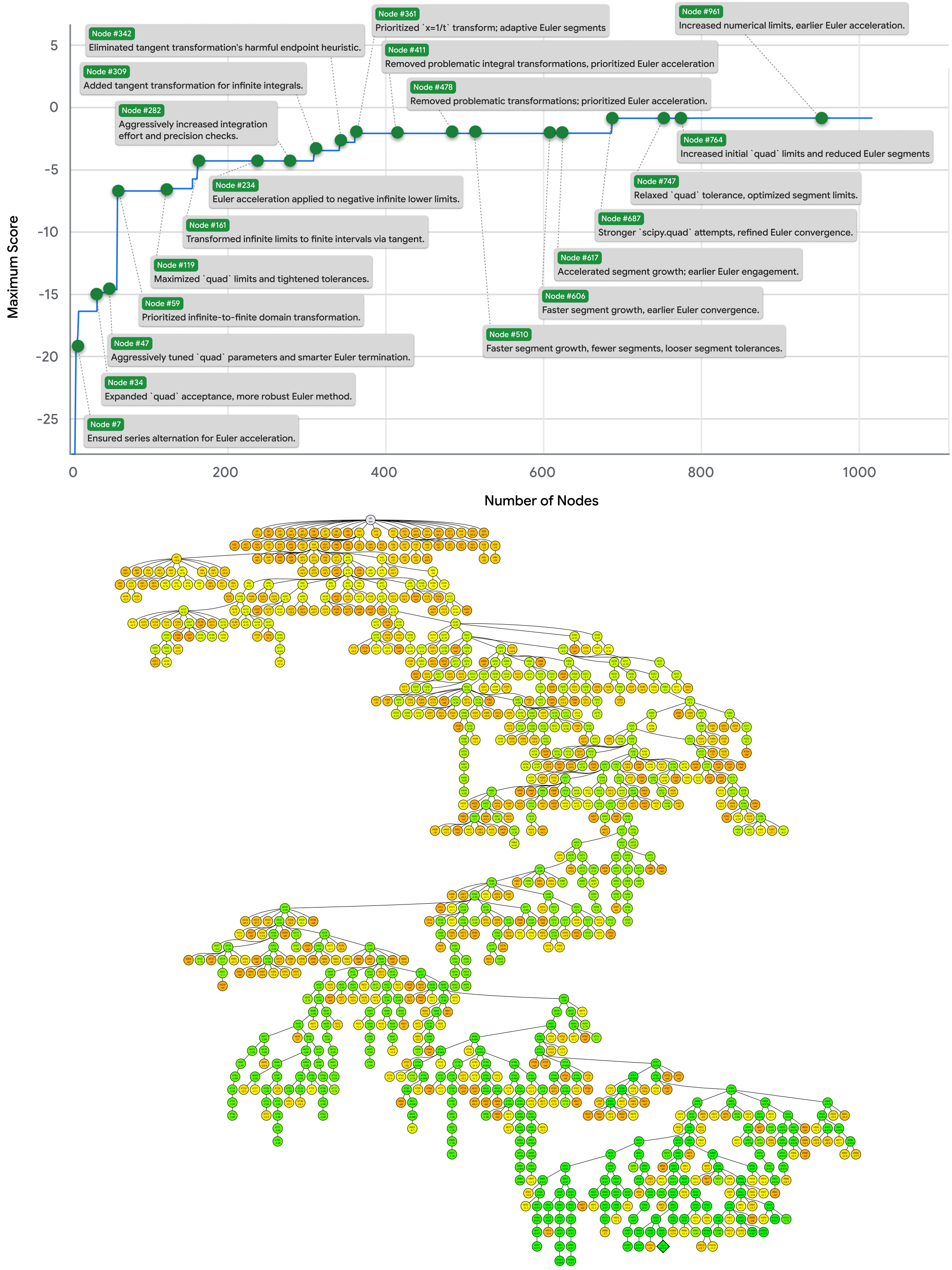

Supplementary Fig. S19 | Breakthrough plot and solution tree for the numerical integration task. *Top Figure* Breakthrough plot for the Integral tree search, showing the evolution of the maximum score as a function of the number of nodes. The green dots label places where the score abruptly increases due to an improvement in the code, and the label describes the change in the code that resulted in the score increase. *Bottom Figure* Structure of the tree for this same search. The color range consists of orange (lower scores) to green (higher scores) with the highest score denoted by a diamond node.

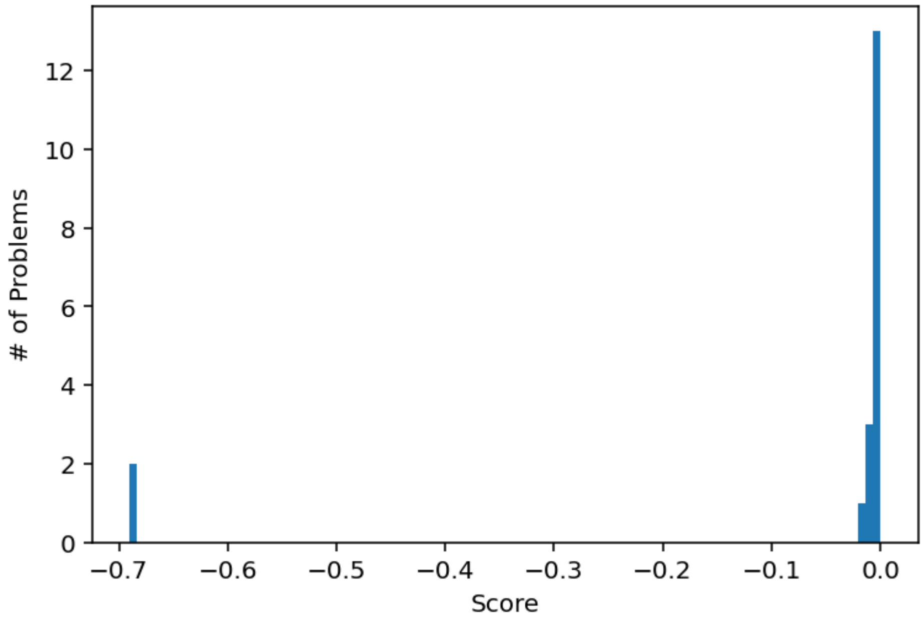

Supplementary Fig. S20 | Scores of the best numerical integration routine applied to the held-out set of 19 integrals. Zero is a perfect score. The generated function solved 17 of 19 integrals to within 3 percent. The standard function, `scipy.integrate.quad()` failed in all these cases.

3. Supplementary Tables

Supplementary Table S1 | Computational budget and execution costs per search node across evaluated benchmarks. We report the average number of request and response tokens processed by the language model per node, alongside the average sandbox execution duration and hardware requirements. The overall computational cost scales linearly with the total number of nodes explored during the search.

Task	Request Tokens	Response Tokens	Duration (min)	Sandbox Type
Batch Integration	16,171	4,183	8.0	GPU[b]
Covid Forecasting	9,607	3,392	1.2	CPU
GIFT-Eval	15,828	9,308	53.3	CPU
Integrals	224,415[a]	6,074	1.5	CPU
Geospatial	7,186	3,172	16.4	GPU
ZAPBench	16,809	8,036	192.2	GPU

[a] The request token count for the integrals is artificially inflated due to tokenization of images in the prompt.
[b] The GPUs used in this study are NVIDIA Tesla T4.

Supplementary Table S2 | Kaggle Playground Series (Season 3) Competitions included in the experiments.

Competition Name	Episode
Regression with a Tabular California Housing Dataset	s3e1
Binary Classification with a Tabular Stroke Prediction Dataset	s3e2
Binary Classification with a Tabular Employee Attrition Dataset	s3e3
Binary Classification with a Tabular Credit Card Fraud Dataset	s3e4
Tabular Classification with a Wine Quality Dataset	s3e5
Binary Classification with a Tabular Reservation Cancellation Dataset	s3e7
Tabular Regression with a Gemstone Price Dataset	s3e8
Regression with a Tabular Concrete Strength Dataset	s3e9
Binary Classification with a Tabular Pulsar Dataset	s3e10
Regression with a Tabular Media Campaign Cost Dataset	s3e11
Binary Classification with a Tabular Kidney Stone Prediction Dataset	s3e12
Regression with a Wild Blueberry Yield Dataset	s3e14
Regression with a Crab Age Dataset	s3e16
Binary Classification of Machine Failures	s3e17
Forecasting Mini-Course Sales	s3e19
Predict CO2 Emissions in Rwanda	s3e20

Supplementary Table S3 | Prompt for Kaggle Playground competitions. The prompt is used for ERA on the Kaggle Playground Benchmark. This example is for Season 3 Episode 17.

Prompt for Kaggle Playground Competitions

Please write the python code to work on a Kaggle competition. Use any model you like.
Kaggle competition name: Binary Classification of Machine Failures
The competition is evaluated as follows: Submissions are evaluated on area under the ROC curve between the predicted probability and the observed target.

```
Submission File
For each `id` in the test set, you must predict the probability of a
`Machine failure`.  The file should contain a header and have the following
format:

    id,Machine failure
    136429,0.5
    136430,0.1
    136431,0.9
    etc.

Here are a few lines of each of the files:

file_name : sample_submission.csv

file_contents:

id,Machine failure
79996,0
100009,0
etc.

====
file_name : test.csv

file_contents:
etc.
====
file_name : train.csv

file_contents:
etc.
====
Please provide complete code that will generate the submission file in the
format below:

```python
YOUR CODE
```
```

Supplementary Table S4 | Expert advice for Kaggle Playground competitions. The prompt is used for the TS with Expert Advice on the Kaggle Playground Benchmark.

Expert advice prompt for Kaggle Playground competitions

Here is high level advice: Instead of putting all your effort into a single model, experiment with combining two or more models. Start with simple averaging of predictions and then explore more advanced techniques like stacking.
Try out several different types of models (e.g., gradient boosting machines, linear models, and even simpler models like logistic regression) to see how they perform.
Look for opportunities to go beyond standard preprocessing. Investigate the data for potential leaks, and consider using optimization libraries to find the best way to combine your models' predictions.
While feature engineering is a crucial skill, it's also important to recognize when it might not be the most important factor. Sometimes, the choice of model and ensembling strategy can have a bigger impact. Don't be afraid to try a more "brute-force" approach with powerful models that can handle raw data effectively.

Supplementary Table S5 | Boosted decision trees for Kaggle Playground competitions. The prompt is used for the TS with Boosted Decision Tree on the Kaggle Playground Benchmark.

Expert Advice Prompt for Kaggle Playground Competitions

Given the code you are given please rewrite any library code (such as XGBoost, LightGBM, and CatBoost) by making internal algorithmic choices that produce performant training code and models that generalize well in many situations. Things you can try are alternative representations of data, using different step size algorithms, using the output of a strong learner as input to the next weak learner. If the code contains such libraries, please extract the raw code that is being used in the library and rewrite it to improve performance.

Supplementary Table S6 | Example code generated by ERA. We prompted ERA to generate a solution for single-cell batch integration. We refer to this solution as `BBKNN (TS)`.

Example code generated by ERA.

```
from typing import Any
from sklearn.decomposition import TruncatedSVD
from sklearn.neighbors import NearestNeighbors
from scipy.sparse import lil_matrix, csr_matrix
import numpy as np
import scanpy as sc
import anndata as ad
import heapq # For efficiently getting top K elements from merged lists

# Define parameters for the config.
# These values are chosen to balance computational cost and integration performance
# for datasets with up to ~300k cells and 2k genes.
config = {
    'n_pca_components': 100,      # Number of PCA components. Recommended: 50-200.
                                  # Captures sufficient variance while reducing dimensionality.
    'n_neighbors_per_batch': 10, # Number of neighbors to find within each batch. Recommended: 5-15.
                                  # This defines the local batch context for each cell.
    'total_k_neighbors': 50,      # Total number of nearest neighbors to retain for the final graph. Recommended:
     15-100.
                                  # This forms the global batch-integrated graph.
}

def eliminate_batch_effect_fn(
    adata: ad.AnnData, config: dict[str, Any]
) -> ad.AnnData:
  # Create a copy to ensure the original input adata remains unchanged.
  adata_integrated = adata.copy()

  # --- Preprocessing: Normalize, log-transform, scale ---
  # These are standard initial steps for scRNA-seq data.
  # Use adata.X which contains raw counts.
  sc.pp.normalize_total(adata_integrated, target_sum=1e4)
  sc.pp.log1p(adata_integrated)
  sc.pp.scale(adata_integrated, max_value=10) # Clip values to avoid extreme outliers

  # --- Batch Correction: ComBat on the gene expression matrix ---
  # This step applies a more robust linear model-based batch correction
  # directly on the gene expression data before dimensionality reduction.
  # ComBat modifies adata_integrated.X in place.
  sc.pp.combat(adata_integrated, key='batch')

  # --- Dimensionality Reduction: PCA on the ComBat-corrected data ---
  # n_comps cannot exceed min(n_obs - 1, n_vars). Robustly handle small datasets.
  n_pca_components = config.get('n_pca_components', 100)
  actual_n_pca_components = min(n_pca_components, adata_integrated.n_vars, adata_integrated.n_obs - 1)

  # Handle edge cases for PCA and graph construction where data is too small.
  # If PCA cannot be run meaningfully, return a minimal AnnData object to avoid errors.
  if actual_n_pca_components <= 0 or adata_integrated.n_obs <= 1:
      print(f"Warning: Too few observations ({adata_integrated.n_obs}) or dimensions ({adata_integrated.n_vars})
     for PCA/graph construction. Returning trivial embedding.")
      # Provide a placeholder embedding and empty graph structure.
      adata_integrated.obsm['X_emb'] = np.zeros((adata_integrated.n_obs, 1))
      adata_integrated.obsp['connectivities'] = csr_matrix((adata_integrated.n_obs, adata_integrated.n_obs))
      adata_integrated.obsp['distances'] = csr_matrix((adata_integrated.n_obs, adata_integrated.n_obs))
      adata_integrated.uns['neighbors'] = {
          'params': {
              'n_neighbors': 0,
              'method': 'degenerate',
              'n_pcs': 0,
              'n_neighbors_per_batch': 0,
              'pca_batch_correction': 'none',
          },
          'connectivities_key': 'connectivities',
          'distances_key': 'distances',
      }
      return adata_integrated

  sc.tl.pca(adata_integrated, n_comps=actual_n_pca_components, svd_solver='arpack')

  # Set the ComBat-corrected PCA embedding as the integrated output embedding.
  # This 'X_emb' will be directly evaluated by metrics like ASW, LISI, PCR.
  adata_integrated.obsm['X_emb'] = adata_integrated.obsm['X_pca']


  # --- Custom Batch-Aware Nearest Neighbors Graph Construction ---
  # This implements the expert advice: find neighbors independently within batches, then merge.
  # This part of the code remains largely the same, but now operates on the
  # ComBat-corrected PCA embedding (adata_integrated.obsm['X_emb']).
  k_batch_neighbors = config.get('n_neighbors_per_batch', 10)
  total_k_neighbors = config.get('total_k_neighbors', 50)

  # A list of dictionaries to store unique neighbors and their minimum distances for each cell.
  # Using dictionaries allows efficient updating if a cell is found as a neighbor from multiple batches.
  merged_neighbors_per_cell = [{} for _ in range(adata_integrated.n_obs)]

  # Group cell indices by batch for efficient querying.
  batches = adata_integrated.obs['batch'].values
  unique_batches = np.unique(batches)
  batch_to_indices = {b: np.where(batches == b)[0] for b in unique_batches}

  # Pre-fit NearestNeighbors models for each batch's data using the corrected PCA embedding.
  # This avoids refitting the model for every query.
  batch_nn_models = {}
  for b_id in unique_batches:
    batch_cell_indices = batch_to_indices[b_id]
    # Ensure there are enough cells to fit a NearestNeighbors model (at least k_batch_neighbors + 1 for self-
     exclusion, or just > 0 for min k=1)
    if len(batch_cell_indices) > 0:
        # Fit with a k that is at most the batch size to avoid errors if k_batch_neighbors is too high for a small
     batch.
        k_fit_effective = min(k_batch_neighbors + 1, len(batch_cell_indices)) # +1 to ensure self-loop can be found
      and excluded
```

Example code generated by ERA (continued).

```
        if k_fit_effective > 0: # Only fit if there are points available
            nn_model = NearestNeighbors(n_neighbors=k_fit_effective, metric='euclidean', algorithm='auto')
            nn_model.fit(adata_integrated.obsm['X_emb'][batch_cell_indices])
            batch_nn_models[b_id] = nn_model

    # Iterate through all possible query batches and target batches to find neighbors.
    for query_batch_id in unique_batches:
        query_global_indices = batch_to_indices[query_batch_id]
        if len(query_global_indices) == 0:
            continue # Skip empty query batches

        query_data = adata_integrated.obsm['X_emb'][query_global_indices]

        for target_batch_id in unique_batches:
            if target_batch_id not in batch_nn_models:
                continue # Skip target batches that were too small to fit an NN model

            nn_model = batch_nn_models[target_batch_id]
            target_global_indices = batch_to_indices[target_batch_id]

            # Ensure n_neighbors does not exceed the number of points in the target batch.
            k_for_query = min(k_batch_neighbors, len(target_global_indices) -1) # -1 to avoid finding self as
   neighbor if batch is query batch
            if k_for_query <= 0: # No valid neighbors can be found in this target batch
              continue

            # Query neighbors for all cells in the current query batch against the target batch's data.
            distances, indices_in_target_batch = nn_model.kneighbors(query_data, n_neighbors=k_for_query,
   return_distance=True)

            for i_query_local in range(len(query_global_indices)):
                current_cell_global_idx = query_global_indices[i_query_local]

                dists_for_cell = distances[i_query_local]
                global_neighbors_for_cell = target_global_indices[indices_in_target_batch[i_query_local]]

                for k_idx in range(len(global_neighbors_for_cell)):
                    neighbor_global_idx = global_neighbors_for_cell[k_idx]
                    dist = dists_for_cell[k_idx]

                    # Exclude self-loops: a cell should not be its own neighbor in graph construction.
                    if neighbor_global_idx == current_cell_global_idx:
                        continue

                    # Store neighbor and its distance. If already present, keep the minimum distance (closest
   connection).
                    if (neighbor_global_idx not in merged_neighbors_per_cell[current_cell_global_idx] or
                        dist < merged_neighbors_per_cell[current_cell_global_idx][neighbor_global_idx]):
                        merged_neighbors_per_cell[current_cell_global_idx][neighbor_global_idx] = dist

    # Convert collected neighbors and distances into sparse matrices.
    rows = []
    cols = []
    data_distances = []

    for i in range(adata_integrated.n_obs):
      # Retrieve all candidate neighbors for cell 'i', sort by distance, and take the top 'total_k_neighbors'.
      current_cell_candidates = list(merged_neighbors_per_cell[i].items())

      if not current_cell_candidates: # If a cell has no valid neighbors after all filtering
          continue

      # Use heapq for efficient selection of the smallest distances.
      selected_neighbors = heapq.nsmallest(total_k_neighbors, current_cell_candidates, key=lambda item: item[1])

      for neighbor_idx, dist in selected_neighbors:
          rows.append(i)
          cols.append(neighbor_idx)
          data_distances.append(dist)

    # Create distance matrix. Handle case with no neighbors found at all for the entire dataset.
    if not rows:
        distances_matrix = csr_matrix((adata_integrated.n_obs, adata_integrated.n_obs))
    else:
        distances_matrix = csr_matrix((data_distances, (rows, cols)), shape=(adata_integrated.n_obs, adata_integrated
   .n_obs))

    # Symmetrize the distance matrix: if A is a neighbor of B, then B is also a neighbor of A,
    # with the distance being the maximum of the two observed distances (ensures undirected graph).
    distances_matrix = distances_matrix.maximum(distances_matrix.T)
    distances_matrix.eliminate_zeros() # Remove any explicit zeros created by max operation

    # Create connectivities matrix (binary representation of connections).
    connectivities_matrix = distances_matrix.copy()
    connectivities_matrix.data[:] = 1.0  # All non-zero entries become 1.0 (connected).
    connectivities_matrix.eliminate_zeros()
    connectivities_matrix = connectivities_matrix.astype(float)

    # Store the custom graph in adata.obsp. These keys are used by scib metrics.
    adata_integrated.obsp['connectivities'] = connectivities_matrix
    adata_integrated.obsp['distances'] = distances_matrix

    # Store parameters in adata.uns['neighbors'] for completeness and scanpy/scib compatibility.
    adata_integrated.uns['neighbors'] = {
        'params': {
            'n_neighbors': total_k_neighbors,
            'method': 'custom_batch_aware_combat_pca', # Reflects the integration strategy
            'metric': 'euclidean',
            'n_pcs': actual_n_pca_components,
            'n_neighbors_per_batch': k_batch_neighbors,
            'pca_batch_correction': 'combat', # Indicates ComBat was applied before PCA
        },
        'connectivities_key': 'connectivities',
        'distances_key': 'distances',
    }

    return adata_integrated
```

Supplementary Table S7 | Prompt for recombination of baseline method ideas. The prompt instructs Gemini to identify the main differences in the principles of top-performing solutions, obtained from tree search runs seeded with baseline methods. This generated summary then serves as part of an explicit instruction for tree search to create hybrid strategies.

Prompt for summarizing differences between two baseline methods.
Compare these two code solutions to the same problem of integrating single-cell batch effects. Explain the main principles that differ between the codes:<br><br>CODE 1: [CODE FROM BASELINE 1]<br><br>CODE 2: [CODE FROM BASELINE 2]

Supplementary Table S8 | Configuration of COVID-19 forecasting data splits.

Strategy	Validation Reference Dates	Test Reference Dates	Horizons	Locations	Metric
**Google Retro. 1**	2024-10-05, 2024-10-12, 2024-10-19, 2024-10-26, 2024-11-02, 2024-11-09	2024-11-16, 2024-11-23, 2024-11-30	1–4 weeks	52 US	WIS
**Google Retro. 2**	2024-10-19, 2024-10-26, 2024-11-02, 2024-11-09, 2024-11-16, 2024-11-23	2024-11-30, 2024-12-07, 2024-12-14	1–4 weeks	52 US	WIS
**Google Retro. 3**	2024-11-02, 2024-11-09, 2024-11-16, 2024-11-23, 2024-11-30, 2024-12-07	2024-12-14, 2024-12-21, 2024-12-28	1–4 weeks	52 US	WIS
**Google Retro. 4**	2024-11-16, 2024-11-23, 2024-11-30, 2024-12-07, 2024-12-14, 2024-12-21	2024-12-28, 2025-01-04, 2025-01-11	1–4 weeks	52 US	WIS
**Google Retro. 5**	2024-11-30, 2024-12-07, 2024-12-14, 2024-12-21, 2024-12-28, 2025-01-04	2025-01-11, 2025-01-18, 2025-01-25	1–4 weeks	52 US	WIS
**Google Retro. 6**	2024-12-14, 2024-12-21, 2024-12-28, 2025-01-04, 2025-01-11, 2025-01-18	2025-01-25, 2025-02-01, 2025-02-08	1–4 weeks	52 US	WIS
**Google Retro. 7**	2024-12-28, 2025-01-04, 2025-01-11, 2025-01-18, 2025-01-25, 2025-02-01	2025-02-08, 2025-02-15, 2025-02-22	1–4 weeks	52 US	WIS
**Google Retro. 8**	2025-01-11, 2025-01-18, 2025-01-25, 2025-02-01, 2025-02-08, 2025-02-15	2025-02-22, 2025-03-01, 2025-03-08	1–4 weeks	52 US	WIS
**Google Retro. 9**	2025-01-25, 2025-02-01, 2025-02-08, 2025-02-15, 2025-02-22, 2025-03-01	2025-03-08, 2025-03-15, 2025-03-22	1–4 weeks	52 US	WIS
**Google Retro. 10**	2025-02-08, 2025-02-15, 2025-02-22, 2025-03-01, 2025-03-08, 2025-03-15	2025-03-22, 2025-03-29, 2025-04-05	1–4 weeks	52 US	WIS
**Google Retro. 11**	2025-02-22, 2025-03-01, 2025-03-08, 2025-03-15, 2025-03-22, 2025-03-29	2025-04-05, 2025-04-12, 2025-04-19	1–4 weeks	52 US	WIS
**Google Retro. 12**	2025-03-08, 2025-03-15, 2025-03-22, 2025-03-29, 2025-04-05, 2025-04-12	2025-04-19, 2025-04-26, 2025-05-03	1–4 weeks	52 US	WIS
**Google Retro. 13**	2025-03-22, 2025-03-29, 2025-04-05, 2025-04-12, 2025-04-19, 2025-04-26	2025-05-03, 2025-05-10, 2025-05-17	1–4 weeks	52 US	WIS
**Google Retro. 14**	2025-04-05, 2025-04-12, 2025-04-19, 2025-04-26, 2025-05-03, 2025-05-10	2025-05-17, 2025-05-24, 2025-05-31	1–4 weeks	52 US	WIS
**All other TS**	2025-02-22, 2025-03-01, 2025-03-08, 2025-03-15, 2025-03-22, 2025-03-29	2025-04-05, 2025-04-12, 2025-04-19	1–4 weeks	52 US	WIS

Supplementary Table S9 | Method descriptions used for replicating COVID-19 models submitted to the CDC's CovidHub.

`CEPH-Rtrend_covid`

"Use a renewal equation method based on Bayesian estimation of Rt from hospitalization data. Model forecasts should be obtained by using a renewal equation based on the estimated net reproduction number Rt. Apply a lowpass filter to the time series of weekly hospitalizations, then interpolate it to daily resolution. Then use MCMC Metropolis-Hastings sampling to estimate the posterior distribution of Rt based on the filtered data, considering an informed prior on Rt based on COVID-19 literature. The estimated Rt in the last weeks of available data is used to forecast Rt in the upcoming weeks, with a drift term proportional to the current incidence. Finally, use the renewal equation with the posterior distribution and trend of the estimated Rt in the most recent weeks of hospitalization data."

`CMU-TimeSeries`

"Use an ensemble of AR-based time-series models, involving a basic quantile autoregression fit using lagged values of covid-related hospitalization counts (normalized by population). The data should be smoothed in time. Fit the model jointly across all jurisdictions using the most recently available 21 days of training data. Learn each of the 23 quantiles using a separate quantile regression with nonnegativity and quantile sorting constraints applied post hoc."

`CMU-climate_baseline`

"Use an ensemble of historically formed quantiles. Using data from 2022 onwards, this climatological model should use samples from the 7 weeks centered around the target week and reference week to form the quantiles for the target week, as one might use climate information to form a meteorological forecast. To get more variation at some potential issue of generalization, one can form quantiles after aggregating across geographic values as well as years (after converting to a rate based case count). This model should use a simple average of the geo-specific quantiles and the geo-aggregated quantiles."

`JHU_CSSE-CSSE_Ensemble`

"Use a Multi-Pathogen Optimized Geo-Hierarchical Ensemble Framework (MPOG-Ensemble). Forecast state-level COVID-19 hospitalizations using a combination of time series forecasting methods, organized across three hierarchical levels. At the individual state level, forecasts are generated using Holt-Winters Exponential Smoothing. For regional predictions, which group states based on past 2 years covid-19 activity trends identified through the Louvain method, Long Short-Term Memory (LSTM) models are employed. Additionally, a LSTM model that covers all states is implemented. These three-tiered model outputs are integrated, selecting weights based on their recent performance in terms of Mean Absolute Error (MAE) to produce the final prediction."

`OHT_JHU-nbxd`

“Use a neural network that encodes the data inputs using a TCN (Bai et al. 2018) and decodes the result into a forecast using N-BEATS (Oreshkin et al. 2000). This is a residual block type architecture that generates point forecasts from univariate time series data. The network accepts a fixed lookback window of time points as input, and has a set number of output nodes corresponding to the length of the forecast horizon. Extend the network with additional residual blocks that output error variance forecasts (evaluated using a likelihood loss function) which allows generating quantile forecasts, assuming a parametric (gamma) error distribution. Additional predictor variables are incorporated using a temporal convolutional network (TCN; Bai et al. 2018). The TCN accepts one input channel for each predictor time series (or static variable), including past values of the target variable, and outputs a single channel with the same length as the lookback window. The TCN output channel is used as the input to the extended N-BEATS network. Each value in the TCN output sequence is a non-linear combination of the predictor variables at that point and all previous points in the lookback window, which preserves the temporal structure of the input. Forecast is the median of an ensemble of such models with varying lookback window sizes and random initializations."

`UM-DeepOutbreak`

“Use a deep neural network model with conformal predictions. The neural network architecture is a sequence-to-sequence model based on recurrent units and self-attention modules. It is trained in a multi-task setting where each region is considered a task. The uncertainty quantification is conducted post hoc with conformal predictions that follows adaptive conformal inference to adapt to distribution shifts. Spatial correlation is not considered."

`UMass-ar6_pooled`

“Use an autoregressive model with shared coefficients across locations: AR(6) model after fourth root data transform. AR coefficients are shared across all locations. A separate variance parameter is estimated for each location."

`UMass-gbqr`

“Use gradient boosting quantile regression. Do gradient boosting using features summarizing signal activity, properties of the location, information about the timing of forecast creation, and the forecast horizon."

Supplementary Table S10 | Prompt for replicating COVID-19 models submitted to CovidHub by injecting method descriptions as `{method}` into existing tree search prompt.

Prompt for replicating models submitted to CovidHub.

Please write the python code to work on a competition.
`{method}`
I've already loaded the train / test files and split out the x and y parts.
Please provide a new definition for the function below, complete with imports, that will generalize well. However, do not do any cross-validation in here. Your function should expect options to be passed in via the config argument. I'll use cross-validation myself to select which of the options in the `config_list` generalizes best.
`{method}`

```
from typing import Any  # Don't forget this!
import pandas as pd

def fit_and_predict_fn(
   train_x: pd.DataFrame,
   train_y: pd.Series,
   test_x: pd.DataFrame,
   config: dict[str, Any]) -> pd.Series:
    """Make predictions for test_x by modeling train_x to train_y.
    Do not do any cross-validation in here.
    """
    mean_y = np.mean(train_y)
    return pd.Series([mean_y] * len(test_x), index=test_x.index)

    # These will get scored by code that I supply. You'll get back a summary
    # of the performance of each of them.

config_list = [{}]
```

And format it like this:

```
# YOUR CODE
# YOUR config_list
```

Supplementary Table S11 | Expert manual inspection of adherence of ERA implementation to COVID-19 modeling methods.

Method	Judgment	Notes
CEPH-Rtrend_covid x CMU-TimeSeries	Follow	
CEPH-Rtrend_covid x CMU-climate_ baseline	Follow	
CEPH-Rtrend_covid x JHU_CSSE-CSSE_Ensemble	Follow	
CEPH-Rtrend_covid x OHT_JHU-nbxd	Follow	Translates R_t into engineered features (lagged differences, ratios).
CEPH-Rtrend_covid and UM-DeepOutbreak	Follow	Feeds mechanistic-inspired features into GRU-based encoder, predicts quantiles via pinball loss.
CEPH-Rtrend_covid x UMass-ar6_pooled	Follow + Innovate	Simulates from normal distribution in transformed space then inverse transforms to derive quantiles.
CEPH-Rtrend_covid x UMass-gbqr	Follow	Implements mechanistic model components as input features to ML model.
CMU-TimeSeries x CMU-climate_baseline	Follow	AR model with climatological features as predictors.
CMU-TimeSeries x JHU_CSSE-CSSE_Ensemble	Follow	Hierarchical ensemble of QuantReg AR models with performance-based weighting.
CMU-TimeSeries x OHT_JHU-nbxd	Follow	Ensemble of bagged QuantReg AR models.
CMU-TimeSeries x UM-DeepOutbreak	Follow	LightGBM quantile regression models with iterative forecasting + conformal-like calibration.
CMU-TimeSeries x UMass-ar6_pooled	Follow	Ensemble of AR QuantReg models on fourth-root transformed data.
CMU-TimeSeries x UMass-gbqr	Follow	LightGBM quantile models on population-normalized data with (un)smoothed lags + direct multi-horizon prediction.
CMU-climate_baseline x JHU_CSSE-CSSE_Ensemble	Follow	Hierarchical ensemble of climatological models.
CMU-climate_baseline x OHT_JHU-nbxd	Follow	Feeds climatological quantiles into LightGBM to learn directly from seasonal baseline.
CMU-climate_baseline x UM-DeepOutbreak	Follow	LightGBM to predict central trend + climatological model for empirical quantile spreads.
CMU-climate_baseline x UMass-ar6_pooled	Follow	Seasonally-aware method for estimating uncertainty based on empirical quantiles of AR residuals.

Continued on next page

Supplementary Table S11 – continued from previous page

Method	Judgment	Notes
CMU-climate_baseline x UMass-gbqr	Follow	Feeds climatological statistics as features into LightGBM.
JHU_CSSE-CSSE_Ensemble x OHT_JHU-nbxd	Partially Follow	Hierarchical structure (state, regional, national models) + adaptive MAE-weighting.
JHU_CSSE-CSSE_Ensemble x UM-DeepOutbreak	Follow + Innovate	Secondary model to predict error magnitudes & find quantiles of normalized residuals.
JHU_CSSE-CSSE_Ensemble x UMass-ar6_pooled	Follow	
JHU_CSSE-CSSE_Ensemble x UMass-gbqr	Follow	Combines predictions from 'adaptive' model trained on recent data & 'stable' model trained on longer history.
OHT_JHU-nbxd x UM-DeepOutbreak	Follow	
OHT_JHU-nbxd x UMass-ar6_pooled	Follow	Feature engineering + ensembling + variance-stabilizing transformation _ recursive forecasting.
OHT_JHU-nbxd x UMass-gbqr	Follow	Uses LightGBM predicts parameters of Gamma distribution.
UM-DeepOutbreak x UMass-ar6_pooled	Follow	
UM-DeepOutbreak x UMass-gbqr	Follow	
UMass-ar6_pooled x UMass-gbqr	Follow	LightGBM quantile regression on fourth-root transformed target.
DEEP-RESEARCH-CSTGT	Follow	Simplified static graph + synthetically generated policy feature.
DEEP-RESEARCH-MetaEnsembler	Follow	Meta-model to predict WIS.
DEEP-RESEARCH-FairnessAwareOptimization	Follow	Iterative re-weighting approximates composite fairness loss.
DEEP-RESEARCH-RegimeSwitchingDetection	Follow	
CO-SCIENTIST-STGNN-AgACI	Does not Follow	AR quantile regression model using LightGBM. Omits AgACI stage, replaces with simpler post-processing.
CO-SCIENTIST-MAPS	Partially Follow	3-stage ensemble: substitutes core models (GNN, TCN, GPR, MLP) with feature-engineered LightGBM proxies.
DEEP-RESEARCH-GenomiWastewater Fusion	Follow	Uses mock API calls.
DEEP-RESEARCH-AdversarialRecalibration	Follow + Innovate	Implements a post-hoc GAN structure. Composite loss function combining adversarial + pinball loss.
DEEP-RESEARCH-BehavioralSensing	Follow	Simulates external data.

Continued on next page

Supplementary Table S11 – continued from previous page

Method	Judgment	Notes
DEEP-RESEARCH-HierarchicalBayesianNODE	Follow	Three-level model: Negative Binomial observation layer, Neural ODE for jurisdiction-level dynamics, global hyper-priors for partial pooling.
CO-SCIENTIST-HGPC	Partially Follow	LightGBM quantile regression, uses feature engineering as proxy for complex stages.
DEEP-RESEARCH-PIDM	Follow	Implements conditional Denoising Diffusion Probabilistic Model (DDPM) with U-Net backbone, with loss function a weighted composite of standard diffusion loss and a physics-based regularization term derived from an SEIR-H model's outputs. Probabilistic forecasts generated by sampling from the learned reverse process.
CO-SCIENTIST-HQE	Partially Follow	Trains multiple base models, feeds their predictions into a meta-learner, then applies a conformal prediction step to adjust final quantiles. Uses multiple LightGBM models instead of suggested Prophet/TBATS for diversity, manually implements conformal prediction instead of using MAPIE.
DEEP-RESEARCH-CounterfactualSimulation	Follow + Innovate	Follows Monte Carlo structure: defines uncertain drivers with distributions, simulates N trajectories by applying sampled shocks to base median forecast, calculates empirical quantiles. Introduces Poisson noise on top of scenario-driven forecasts.
rep-OHT_JHU-nbxd	Follow	Implements TCN encoder and N-BEATS decoder architecture, including extension of parallel residual blocks to forecast mean and variance for Gamma distribution. The final forecast is generated as a median of an ensemble with varying lookback windows and initializations.
rep-CMU-TimeSeries	Follow	Implements a quantile autoregression model fit jointly across jurisdictions on smoothed, population-normalized data.
rep-UMass-ar6_pooled	Follow	Uses OLS on lagged, fourth-root transformed data to create a shared-coefficient AR model, then calculates separate variance parameters for each location based on residuals.
rep-UM-DeepOutbreak	Follow	Implements sequence-to-sequence model using a GRU and self-attention, with location embeddings. Uncertainty quantified post hoc using split conformal prediction on a recent time window.
rep-UMass-gbqr	Follow	Uses LightGBM with engineered features (lags for signal activity, location and population for location properties, date components for timing, and the horizon itself).
rep-JHU_CSSE-CSSE_Ensemble	Follow + Innovate	Implements three-tiered hierarchical ensemble, using Holt-Winters, regional LSTMs with Louvain grouping, and a national LSTM, combined with MAE-based weighting. Uses scaled residuals to create prediction intervals that adapt to the magnitude of the forecast to generate quantile predictions.

Continued on next page

Supplementary Table S11 – continued from previous page

**Method**	**Judgment**	**Notes**
rep-CMU-climate_baseline	Follow + Innovate	Averages geo-specific and geo-aggregated quantiles within a centered weekly window. Introduces a configurable 'smoothing_factor', which regularizes final predictions by pulling them towards zero.
rep-CEPH-Rtrend_covid	Follow	Lowpass filtering, daily interpolation, MCMC for Bayesian Rt estimation, and a renewal equation forecast. The Rt forecast correctly incorporates a sophisticated drift term that is modulated by the current incidence level.
retro_1	Follow	

Supplementary Table S12 | Full GIFT-Eval leaderboard (05/18/2025 snapshot).

Model	MASE	Type
**Per-dataset**	**0.671**	**ERA**
TTM-R2-Finetuned	0.679	fine-tuned
timesfm_2_0_500m	0.680	pretrained
TabPFN-TS	0.692	pretrained
chronos_bolt_base	0.725	pretrained
**Unified**	**0.734**	**ERA**
chronos_bolt_small	0.738	pretrained
PatchTST	0.762	deep-learning
TEMPO_ensemble	0.773	fine-tuned
VisionTS	0.775	pretrained
Chronos_large	0.781	pretrained
Moirai_large	0.785	pretrained
Chronos_base	0.786	pretrained
Chronos_small	0.800	pretrained
Moirai_base	0.809	pretrained
TFT	0.822	deep-learning
N-BEATS	0.842	deep-learning
Moirai_small	0.849	pretrained
TTM-R2-Zeroshot	0.915	pretrained
DLinear	0.952	deep-learning
Auto_Arima	0.964	statistical
TimesFM	0.967	pretrained
TTM-R1-Zeroshot	0.969	pretrained
Auto_Theta	0.978	statistical
TIDE	0.980	deep-learning
Seasonal_Naive	1.000	statistical
Timer	1.019	pretrained
Auto_ETS	1.088	statistical
Lag-Llama	1.102	pretrained
DeepAR	1.206	deep-learning
Naive	1.260	statistical
Crossformer	2.310	deep-learning

Supplementary Table S13 | Example configurations from the final `unified solution` for the GIFT-Eval task. Each dictionary defines a complete forecasting strategy discovered by the tree search, combining different components of the Iterative Decomposition Model. The validation process selects the best configuration for each dataset.

Unified Solution Example Configurations

```
config_list = [
  {
      'name': 'seasonal_naive_baseline',
      'description': 'Robust baseline...',
      'components': [{'type': 'base', 'method': 'seasonal_naive_adaptive'}],
      'transform_log': False, 'non_negative': False, 'version': 4,
  },
  {
      'name': 'additive_damped_linear_LogTransform',
      'description': 'General-purpose additive model...',
      'components': [
          {'type': 'base', 'method': 'median_all'},
          {'type': 'trend', 'method': 'polynomial', 'degree': 1, 'damping_factor': 0.90},
          {'type': 'seasonal', 'method': 'average', 'window_multiplier': 5.0},
          {'type': 'residual', 'method': 'median', 'window_size': 18, 'decay_factor': 0.90},
      ],
      'transform_log': True, 'non_negative': True, 'version': 4,
  },
  {
      'name': 'date_features_seasonal',
      'description': 'Robust additive model with key cyclical and datetime features...',
      'components': [
          {'type': 'base', 'method': 'median_all'},
          {'type': 'datetime', 'features': [
              ['dayofweek', 'hour'], 'month', 'is_month_start', 'weekofyear',
              'is_weekend', 'is_quarter_start',
              {'name': '_is_holiday_flag',
               'country_codes': ['US', 'DE', 'CN', 'GB', 'CA', 'AU']}
          ]},
          {'type': 'seasonal', 'method': 'average', 'window_multiplier': 4.0},
          {'type': 'residual', 'method': 'median', 'window_size': 14, 'decay_factor': 0.92},
      ],
      'transform_log': False, 'non_negative': False, 'version': 4,
  },
  % ... other configurations can be added here ...
]
```

Supplementary Table S14 | Comparison of model performance on the DLRSD benchmark. The table shows the publication year, architecture, key features, and reported mean Intersection over Union (mIoU) for tree search solutions and the methods from the referenced papers.

Method	Year	Architecture Type	Key Features / Techniques	mIoU
**Solution 1 (TS)**	2025	CNN (UNet++)	'efficientnet-b7' encoder, 8-fold TTA	0.81
**Solution 2 (TS)**	2025	Transformer(SegFormer)	'mit-b1' encoder, 4-fold TTA	0.82
**Solution 3 (TS)**	2025	CNN (U-Net)	'se_resnext101_32x4d' encoder, 7-fold TTA	0.80
RE-Net[21]	2021	CNN (Region-based)	Region Context Learning	0.762
FURSformer[22]	2023	CNN+Transformer	Custom fusion module	0.753
SCGLU-Net[23]	2024	CNN+Attention	Spatial-Channel-Global-Local block	0.666
MA-UNet[24]	2022	Attention+U-Net	Residual encoder with simAM	0.619
W13 Net[25]	2025	CNN (Lightweight)	Multi-stage encoding-decoding	0.580

Supplementary Table S15 | Prompt for Gemini Deep Research to generate ideas to integrate single-cell batch effects.

Prompt for Gemini Deep Research.

I am developing new methods for winning single-cell batch integration competitions, as proposed by the Kaggle and extensively researched in the single-cell genomics community.

Briefly: Modelers are asked to develop a function, `eliminate_batch_effect_fn`, that transforms raw gene expression count data from multiple batches into a low-dimensional embedding or feature matrix. This transformed output should effectively remove technical variation (batch effects) while rigorously preserving biological information (e.g., cell type identity). The performance of these methods is evaluated against a suite of metrics that quantify both batch mixing and biological conservation.

The key problem is to develop a method that takes an `AnnData` object of raw gene expression counts with batch labels and returns an AnnData object with a batch-integrated low-dimensional embedding in the `.obsm['X_emb']` field. The method must excel across a diverse set of evaluation metrics, including ASW Batch, ASW Label, ARI, NMI, Graph Connectivity, Isolated Labels ASW, Isolated Labels F1, kBET, iLISI, cLISI, PCR, and Cell Cycle Conservation Score, aiming to maximize their average.

The following principles should be obeyed when choosing models:
* **Batch Effect Removal**: Prioritize techniques that explicitly model and mitigate batch-specific variations without collapsing biological signal.
* **Biological Conservation**: Ensure the integrated representation retains and accurately reflects genuine biological differences, particularly cell type distinctions, as measured by clustering and silhouette metrics.
* **Scalability and Efficiency**: Given the large dataset sizes (e.g., $329,762$ cells $\times$ $2,000$ genes), models must be computationally efficient and avoid out-of-memory errors.
* **Constraint Adherence**: The implementation must strictly avoid using cell_type information during integration and should primarily leverage `scanpy`, `sklearn`, `numpy`, `scipy`, `tensorflow`, `torch`, `jax`, or equivalent native implementations rather than specialized single-cell packages.

This task aims to develop a SUPERHUMAN METHOD for solving this problem.

Please give me 10 highly novel and creative ideas with detailed implementation notes for the set of methods I should explore for solving this task. I aim to create the best method for solving this problem, preferably creating the best ever method.

Supplementary Table S16 | Prompt for formatting Deep Research ideas into a structure similar to baseline method descriptions.

Prompt for formatting Deep Research ideas.

Structure the given idea into the following format:

<description>
Your description about the method goes here.
</description>

<steps>
Your list of steps to implement the method goes here.
</steps>

<notes>
Strengths and weaknesses of the idea goes here.
</notes>

Supplementary Table S17 | Prompt for guiding ERA to generate hybrid strategies.

Prompt for guiding ERA to generate hybrid strategies.

We have up until now done experiments with two major types of codes, that are described in detail below. PLEASE CREATE AN ALGORITHM THAT USES THE BEST PARTS OF BOTH STRATEGIES TO CREATE A HYBRID STRATEGY THAT IS TRULY WONDERFUL AND SCORES HIGHER THAN EITHER OF THE INDIVIDUAL STRATEGIES.

[Output from Supplementary Table S7.]

Supplementary Table S18 | Prompt for summarizing existing batch integration methods. The prompt instructs the LLM to write a brief description and steps of the method. This generated summary then serves as part of an explicit instruction for tree search to create an optimized version of the existing batch integration method. Example output is shown in Supplementary Table S19.

Prompt for summarizing differences between two baseline methods.
Given the following paper, please identify the main method being proposed. Then write a very short method description. This method description will be used to reproduce the method. DO NOT mention the algorithm by name.<br><br>Your output must follow this format:<br><description><br>Your very short description goes here.<br></description><br><br><steps><br>Your short list of steps goes here.<br></steps>

Supplementary Table S19 | Example of existing method description (BBKNN) given to ERA. ERA used this short method description to further optimize the method.

Example of existing method description (BBKNN) given to ERA.

<description>
This method performs batch correction by modifying the neighborhood graph construction step. For each cell, its nearest neighbors are identified independently within each batch, rather than across the entire combined dataset. The resulting batch-specific neighbor lists for each cell are then merged to create a single, integrated graph. This approach assumes that shared cell types exist across batches and that biological differences are greater than technical batch effects.
</description>

<steps>

1. For each cell, iterate through every batch in the dataset.
2. Find the k-nearest neighbors for the cell from within the current batch, based on a given distance metric (e.g., Euclidean distance in PCA space).
3. After iterating through all batches, merge the identified neighbor sets for the cell into a single neighborhood.
4. Repeat for all cells to construct a batch-corrected neighborhood graph.

</steps>

4. Supplementary References

[1] Shao, Z., Yang, K. & Zhou, W. Performance evaluation of single-label and multi-label remote sensing image retrieval using a dense labeling dataset. *Remote Sens.* **10**, 964 (2018).

[2] Yang, Y. & Newsam, S. Bag-of-visual-words and spatial extensions for land-use classification. In *Proc. 18th SIGSPATIAL Int. Conf. on Adv. in Geogr. Inf. Syst.*, 270–279 (Association for Computing Machinery, 2010).

[3] Russakovsky, O. *et al.* ImageNet large scale visual recognition challenge. *Int. J. Comput. Vis.* **115**, 211–252 (2015).

[4] Krizhevsky, A., Sutskever, I. & Hinton, G. E. ImageNet classification with deep convolutional neural networks. *Adv. Neural Inf. Process. Syst.* **25** (2012).

[5] Lueckmann, J.-M. *et al.* ZAPBench: a benchmark for whole-brain activity prediction in zebrafish. *arXiv preprint arXiv:2503.02618* (2025).

[6] Immer, A. *et al.* Forecasting whole-brain neuronal activity from volumetric video. *arXiv preprint arXiv:2503.00073* (2025).

[7] Zeng, A., Chen, M., Zhang, L. & Xu, Q. Are transformers effective for time series forecasting? In *Proc AAAI Conf. Artif. Intell.*, vol. 37, 11121–11128 (2023).

[8] Das, A. *et al.* Long-term forecasting with TiDE: Time-series Dense Encoder. *Trans. Mach. Learn. Res.* (2023).

[9] Chen, S.-A., Li, C.-L., Yoder, N., Arik, S. O. & Pfister, T. TSMixer: An All-MLP architecture for time series forecasting. *Trans. Mach. Learn. Res.* (2023).

[10] Perez, E., Strub, F., De Vries, H., Dumoulin, V. & Courville, A. FiLM: Visual reasoning with a general conditioning layer. In *Proc AAAI Conf. Artif. Intell.*, vol. 32 (2018).

[11] Deistler, M. *et al.* Differentiable simulation enables large-scale training of detailed biophysical models of neural dynamics. *bioRxiv* 2024–08 (2024).

[12] Hodgkin, A. L. & Huxley, A. F. A quantitative description of membrane current and its application to conduction and excitation in nerve. *J. Physiol.* **117**, 500 (1952).

[13] Piessens, R., de Doncker-Kapenga, E., Überhuber, C. W. & Kahaner, D. *QUADPACK: a subroutine package for automatic integration* (Springer-Verlag, 1983).

[14] Gradshteyn, I. & Ryzhik, I. *Table of integrals, series, and products, 8th edn* (Academic Press, 1994).

[15] Meurer, A. *et al.* SymPy: symbolic computing in Python. *PeerJ Comput. Sci.* **3**, e103 (2017).

[16] Brenner, M. P., Cohen-Addad, V. & Woodruff, D. Solving an open problem in theoretical physics using ai-assisted discovery. *arXiv preprint arXiv:2603.04735* (2026).

[17] Lueckmann, J.-M., Jain, V. & Januszewski, M. Discovering mechanistic models of neural activity: System identification in an in silico zebrafish (2026). URL https://arxiv.org/abs/2602.04492. 2602.04492.

[18] McInnes, L., Healy, J. & Melville, J. UMAP: uniform manifold approximation and projection for dimension reduction. *arXiv preprint arXiv:1802.03426* (2018).

[19] Lee, J. *et al.* Gemini Embedding: Generalizable embeddings from Gemini. *arXiv preprint arXiv:2503.07891* (2025).

[20] Ho, S. L. & Xie, M. The use of ARIMA models for reliability forecasting and analysis. *Comput. Ind. Eng.* **35**, 213–216 (1998).

[21] Zhong, B., Du, J., Liu, M., Yang, A. & Wu, J. Region-enhancing network for semantic segmentation of remote-sensing imagery. *Sensors* **21** (2021).

[22] Zhang, Z., Liu, B. & Li, Y. FURSformer: semantic segmentation network for remote sensing images with fused heterogeneous features. *Electronics* **12** (2023).

[23] Atiampo, A. K. & Diédié, G. H. F. New fusion approach of spatial and channel attention for semantic segmentation of very high spatial resolution remote sensing images. *Open J. Appl. Sci.* **14**, 288–319 (2024).

[24] Sun, Y., Bi, F., Gao, Y., Chen, L. & Feng, S. A multi-attention UNet for semantic segmentation in remote sensing images. *Symmetry* **14**, 906 (2022).

[25] Elgamily, K. M., Mohamed, M. A., Abou-Taleb, A. M. & Ata, M. M. A novel W13 deep CNN structure for improved semantic segmentation of multiple objects in remote sensing imagery. *Neural Comput. Appl.* **37**, 5397–5427 (2025).
