# Supplementary material for: An AI system to help scientists write expert-level empirical software
Source: Nature. 2026 May 19;654(8120):909–16. doi: 10.1038/s41586-026-10658-6 (PMC13293872; doi:10.1038/s41586-026-10658-6)
Supplement: Supplementary file 2 — Peer Review File [file 41586_2026_10658_MOESM2_ESM.pdf]

nature portfolio

Peer Review File

An AI system to help scientists write expert-level empirical software

Corresponding Author: Dr Michael Brenner

Any redactions in this file are there to maintain patient confidentiality, the confidentiality of unpublished data, or to remove third-party material.

This file contains all reviewer reports in order by version, followed by all author rebuttals in order by version.

Version 0:

Reviewer comments:

Referee #1

(Remarks to the Author)
Summary

The authors propose an AI-based system that autonomously generates and optimizes scientific software. The approach combines a large language model with Monte Carlo Tree Search (MCTS) to iteratively improve empirical software according to a measurable performance score. The authors define empirical software as code designed to solve “scorable tasks,” i.e., problems where quantitative evaluation metrics are available.

By framing software creation as a search over programs that maximize such metrics, the system is able to explore, refine, and evaluate candidate solutions in an empirically grounded fashion. The method aims to accelerate the development of high-quality computational tools in scientific domains where empirical experimentation is typically time-consuming and limited by human effort.

The proposed methods generate multiple candidate code solutions and apply MCTS to determine which candidates merit further exploration. Each node in the search tree corresponds to a code variant, and expansion decisions are guided by a PUCT selection rule that balances exploration and exploitation, which is an adaptation of the Upper Confidence Bound (UCB) used in traditional MCTS (as used in AlphaZero). Code generation itself follows an iterative mutation process, where an LLM rewrites existing code to improve the measured score. This process is augmented by the injection of “research ideas,” drawn from external sources such as academic papers, textbooks, or search results, which can either be automatically retrieved or manually provided by the user. In this way, the system integrates domain knowledge into the search loop.

The system was initially refined on a curated set of Kaggle playground competitions, which served as a benchmark environment. Finally the authors evaluated their approach across six scorable tasks drawn from diverse scientific domains, including genomics, neuroscience, public health, geospatial analysis, time-series forecasting, and numerical analysis. Task selection followed three criteria: (i) areas where recent methodological progress has been slow, (ii) relevance to active scientific questions, and (iii) direct usefulness to the research agenda of at least one co-author. This selection aims to demonstrate both the breadth of applicability and the practical scientific value of the proposed method.

Strengths

The paper is well motivated, drawing on the success of MCTS in accelerating complex search and optimization problems across domains such as games, planning, and automated reasoning. Adapting this paradigm to the creation of scientific software is a natural and innovative extension, as it provides a principled way to explore vast program spaces where direct optimization is infeasible.

The authors identify a timely and relevant problem: writing and refining scientific software is often slow, manual, and domain-specific, creating a significant bottleneck for research progress. Addressing this challenge through an automated,

performance-guided framework is conceptually sound and could be practically valuable to practitioners of numerous scientific fields.

The methods generated for the scientific tasks appear to be immediately relevant to a wide range of scientific disciplines. In general, fields that rely on computational experimentation could benefit from methods that accelerate software development and optimization while maintaining domain-specific rigor.

A notable strength of the method is its capacity to incorporate human knowledge. Researchers can inject domain insights, hypotheses, or literature-based ideas into the search process, enabling a hybrid workflow that combines human expertise with large-scale automated exploration. This positions the work as a step towards a human–machine collaborative research paradigm, accelerating scientific progress in various fields.

The outcome of the optimization process is executable code, which is generally interpretable and verifiable by human experts. This transparency distinguishes the method from many black-box AI systems and contributes to its credibility and potential for adoption in scientific contexts.

The method proves to be competitive across the diverse set of benchmarks selected by the authors, spanning areas such as genomics, public health forecasting, and geospatial analysis. This breadth of applicability underscores the generality of the approach, which is particularly relevant given its intended role as a tool to support and accelerate research across multiple scientific domains.

The authors also provide an interactive visualization interface that makes the optimization process and intermediate solutions transparent. The visual summaries of score improvements and tree structures substantially improve explainability and facilitate critical assessment by users.

The datasets and benchmark sources used for evaluation are publicly accessible, enabling independent verification and reproducibility of the reported results. The authors' clear documentation and citation of data sources further strengthen the work's transparency and scientific robustness.

Weaknesses

Solving scientific problems in the strict sense involves reasoning about theory, mechanisms, or mathematical frameworks. These are tasks that humans can perform with limited data and without relying on predictive modeling. The system presented in this paper, with its iterative search guided by external research ideas, could in principle support this kind of higher-level reasoning. However, the chosen benchmarks do not reflect this potential. Five of the six evaluated tasks are predictive modeling problems, and only the numerical integration example approaches scientific inference, albeit in a computational sense. As a result, the evaluation primarily demonstrates that the method is capable of engineering and optimization performance rather than genuine scientific discovery.

The paper tends to overstate the point of scientific discovery by implying that the methodology "solves scientific problems," although the demonstrated applications remain within the realm of performing predictive modelling in the context of machine learning. While the distinction between conducting science and supporting scientific work is stated clearly in the abstract and introduction, it becomes less precise later on (for example, in the caption of Figure 1 and in several passages on pages 15–17). Reframing such statements to emphasize that the system automates empirical software development in service of scientific inquiry, rather than performing scientific reasoning itself, would improve conceptual clarity and align the claims more closely with the evidence.

The code availability is insufficient for reproducing the main results. The authors have open-sourced only the code produced by their method, not the code for the optimization process itself. Therefore it is not possible for the reviewers to reproduce the experiments nor for practitioners and scientists to apply the method to new problems. This is inconsistent with Nature's reporting standards, which explicitly require authors to make all materials, data, code, and associated protocols available unless restrictions are clearly disclosed. No such disclosure appears in the manuscript or the submission materials. Beyond policy compliance, the absence of the optimization framework significantly limits the practical impact of the work: practitioners cannot verify the approach, extend it to other domains, or benchmark it against alternative systems. (This is not a reason for rejection, only a downside.)

While the method automates the creation and optimization of code, the scoring of candidate solutions remains the responsibility of the user. The paper does not discuss how a scientist would design or implement such scoring schemes in practice, even though this step is central to applying the method to new domains. A short section or example illustrating this process would make the approach more accessible.

The discussion of related work is limited. Several recent systems also perform iterative improvement of code based on scorable tasks, including Llamea [1], EoH [2], and ReEvo [3]. Situating the contribution relative to these systems and explicitly acknowledging their similarities and differences would substantially improve the contextual framing of the paper.

The manuscript characterizes AutoML as the automation of model and hyperparameter search, which is an oversimplification. Modern AutoML frameworks encompass broader functionality, including feature engineering, meta-learning, pipeline construction, and multi-objective optimization. Moreover, several agentic AutoML systems for automated data science already exist, such as AutoGluon assistant / ML-Zero, AgentK, AutoKaggle, etc. While the authors' system

indeed extends these ideas toward general-purpose code generation and literature-informed search, more accurate and nuanced description of AutoML would help clarify how the present work fits into and advances this line of research.

The description of the search algorithm lacks sufficient technical detail. The paper replaces the traditional Upper Confidence Bound (UCB) with a “Predictor + Upper Confidence bound applied to trees” (PUCT) strategy, yet this modification is only discussed superficially.

The pseudo-code provided omits crucial components: it remains unclear what the “GenerateAndExecute” function entails, how new nodes are generated, and what exactly constitutes a node (whether it represents a research idea, a piece of code, or both). Based on the examples, mutations appear to occur directly at the code level, but this should be explicitly clarified.

The baseline comparisons used for the Kaggle benchmark are missing relevant baselines. The “single sample” and “best of 1000 samples” conditions are weak baselines and do not adequately represent the current state of automated data science systems. The external comparison against AIDE is outdated, as more recent frameworks such as Agent K [7], MLZero [8], and AutoKaggle [9] address similar goals and would serve as stronger points of reference. The fact that this is a submission to Nature would otherwise (inaccurately) suggest to readers that it presents a state-of-the-art solution for Kaggle datasets. This limitation should therefore be stated explicitly.

Furthermore, the prompt for Kaggle datasets describes a highly specific and quite non-human-like approach of writing an entire new gradient boosting library from scratch. This does not yield a very interpretable solution and does not effectively use the domain knowledge a data scientist would have.
The evaluation on the GIFT-Eval benchmark appears outdated relative to the current state of the leaderboard. The paper reports results based on a “snapshot” of the leaderboard from May 18, 2025 that predates several strong recent baselines, including TiRex [4], Toto [5], and Timecopilot [6] (let alone yet newer methods like Chronos 2). Timecopilot, in particular, would provide a highly relevant comparison given its agent-based design, which parallels the authors’ approach. Moreover, the evaluation focuses exclusively on point forecasts and does not include distribution-based metrics, such as the Continuous Ranked Probability Score (CRPS), which are standard in time-series forecasting. Updating the evaluation to include these baselines and probabilistic metrics would give a more accurate and comprehensive picture of the method’s competitiveness. Similar to the Kaggle evaluation, given that this is a submission to Nature, without this comparison, readers would inaccurately assume that this presents a new state-of-the-art solution for time series. Indeed, the abstract falsely states this.

The paper does not report or discuss the computational budget required for its method, including token usage, runtime, or GPU hours. Such information is essential for full assessment and fair comparison, as search-based LLM optimization can be substantially more resource-intensive than single-shot generation. Discussing the computational cost would significantly improve the credibility of the reported results.

Minor Comments

In the statistical analysis on page 7 a t-test to compare cosine similarity scores of text embeddings is applied. However, cosine similarities are bounded between -1 and 1 and typically deviate strongly from normality, violating the assumptions of a t-test. Using this test gives a misleading impression of verifiability. A visualization-based approach, such as displaying projections of the embeddings, would more appropriately convey how distinct the code clusters are without relying on questionable statistical assumptions.

On page 2 (“Overview of Scorable Tasks”), the manuscript states that tasks were selected “using two criteria,” yet three are subsequently listed.

Figure 1 could be improved for coherence. Panel 1b presents empirical results, which feels inconsistent alongside Panels 1a and 1c that explain the methodology. Relocating 1b to the Results section would make the figure more focused.

Panel 1a could be visually refined for clarity, and Supplementary Figure 22, which offers a clearer depiction of the workflow, might be better suited for inclusion in the main text.

Several formatting and terminology issues could be addressed. Some hyperlinks on page 19 cross page boundaries, and a few figures and supplementary sections refer to “agents,” whereas the main text consistently uses “LLM.” For readers unfamiliar with agent-based terminology, this inconsistency can be confusing. The term “agent,” which does accurately describe the system’s behavior (e.g., page 4: “the agent discovers strategies”), should be explicitly introduced and defined when first used.

Recommendation

While we believe that the current submission does not pass the bar of Nature, we invite the authors to resubmit after major modifications.

Areas Outside Reviewer Expertise

The scientific fields of public health, neuroscience, and cell biology fall outside the reviewers’ area of expertise. The review

therefore does not assess the domain-specific validity of the corresponding benchmark results.

Sources
[1] van Stein, Niki, and Thomas Bäck. "Llamea: A large language model evolutionary algorithm for automatically generating metaheuristics." IEEE Transactions on Evolutionary Computation (2024).
[2] Liu, Fei, et al. "Evolution of heuristics: towards efficient automatic algorithm design using large language model." Proceedings of the 41st International Conference on Machine Learning. 2024.
[3] Ye, Haoran, et al. "Reevo: Large language models as hyper-heuristics with reflective evolution." Advances in neural information processing systems 37 (2024): 43571-43608.
[4] Auer, Andreas, et al. "TiRex: Zero-Shot Forecasting Across Long and Short Horizons with Enhanced In-Context Learning." arXiv preprint arXiv:2505.23719 (2025).
[5] Cohen, Ben, et al. "This Time is Different: An Observability Perspective on Time Series Foundation Models." arXiv preprint arXiv:2505.14766 (2025).
[6] Garza, Azul, and Reneé Rosillo. "TimeCopilot." arXiv preprint arXiv:2509.00616 (2025).
[7] Bou-Ammar, Haitham, et al. "Kolb-Based Experiential Learning for Generalist Agents with Human-Level Kaggle Data Science Performance." (2025).
[8] Fang, Haoyang, et al. "Mlzero: A multi-agent system for end-to-end machine learning automation." arXiv preprint arXiv:2505.13941 (2025).
[9] Li, Ziming, et al. "AutoKaggle: A Multi-Agent Framework for Autonomous Data Science Competitions." ICLR 2025 Third Workshop on Deep Learning for Code.

(Remarks on code availability)
Please see the main review for comments on code availability.

Referee #2

(Remarks to the Author)
I co-reviewed this manuscript with one of the reviewers who provided the listed reports.

(Remarks on code availability)

Referee #3

(Remarks to the Author)
Summary and Main Claims
The paper proposes using large language models combined with tree search (LLM+TS) to rapidly improve existing scientific software methods across multiple domains. The core approach involves searching the program space by mutating existing programs guided by domain information. The central claim is that this represents a fast and accurate method for software optimization that can be applied to hard coding problems across diverse domains.

Strengths
The paper tackles genuinely difficult coding problems, and the ability to solve them is impressive. The integration of program synthesis with 'initial idea search" is novel, while related to a lot of prior work in Genetic Programming (GP) before LLMs that integrated domain knowledge. Two examples: GP used grammars and program metrics (Schweim, 2021) (Schweim, 2022). It appears also, given the (slightly vague) description provided here, to be somewhat like GP approaches with LLMs that use the LLM to initialize a population of solutions. In these approaches, the bias of the model (arising from pre-training) is an implicit form of domain knowledge. Additionally, the tree-based guidance mechanism for search is a novel contribution, though it requires clearer exposition.

The breadth of the results is commendable, with some sort of optimization or further investigation performed within each problem domain. It spans multiple scientific domains demonstrating the generalizability of the approach.

Weaknesses
The paper does not present a clear research question, prior hypotheses, or conclusions drawn systematically from experimentation. There is minimal discussion of failure cases or conditions under which the method struggles.

The paper provides no description of computational costs and resources consumed during the search process. Standard practice in the GP and related evolutionary computation literature requires explicit reporting of the number of fitness evaluations, hardware specifications, model details, computational duration, and financial cost. This omission makes it impossible to assess the practical feasibility or efficiency of the method. It make replicability impossible.

The work lacks essential elements of rigorous empirical evaluation: (1) replicability details including specific models, API versions, and implementation details; (2) robustness assessment through multiple runs with statistical analysis; (3) ablation studies to identify which components contribute to success; (4) sensitivity analysis across different LLM families, versions, and costs; and (5) comparison with alternative methods. The method's sensitivity to hyperparameters (e.g., tree search size, node evaluation time) is not discussed. For example, Figure 1b does not make it clear whether there are statistically significant differences between the methods. For example, the results conflate improvements from algorithmic innovation

with performance gains from software optimization. A breakdown is needed: how much speedup came from discovering faster algorithms versus optimizing implementation details? Given a fixed computational budget, did faster algorithms enable more evaluations within the search process itself?

Descriptions require more clarity. For example:
- The tree-based search mechanism is inadequately described—for example, it is unclear whether it relates to (1+5) evolution strategies, beam search, or standard tree search variants.
- "three in parallel" is unexplained.
- Figure 1c and its inline explanation are not explanatory enough
- The differences between what was done during development and training using Kaggle competitions vs what was done during evaluation with the set of scientific coding problems is unclear.

From an evolutionary computation perspective, the recombination approach appears similar to work by (Meyerson, 2024), but with careful parent selection;.

The claim that software development is slow lacks supporting data or citations.

While evolved solutions can be inspected, they remain difficult to understand and validate. The generated methods may lack the explainable arguments needed to justify their correctness. This is not considered.

The results presentation becomes formulaic after the initial examples, with reduced depth in later experiments. This suggests either space constraints forced shortcuts, or the approach's novelty diminishes across repetitions. For an evolutionary computation audience, the result—that a model trained on multi-domain examples performs well across those domains when given appropriate guidance—is not surprising.
There is no source code for the method. Only code for solutions discovered by the method are provided. The discovered solutions are not possible do run without more configuration and setup.

Recent work on LLM-enhanced evolutionary algorithms (Wu, 2024), (Ma, 2025), (Hemberg, 2024) and language model variation (Meyerson, 2024), (Grishina, 2025) address related problems and should be more thoroughly contrasted with this approach. The conceptual framing around "empirical software" and "scorable problems" appears to serve as window dressing for what is fundamentally a program search problem with domain-informed initialization.

Recommendation
While the paper demonstrates promising empirical results on challenging problems, it requires substantial revision to meet publication standards. The authors must provide detailed computational accounting, conduct rigorous ablation and sensitivity studies, discuss failure modes, and clarify the technical contributions relative to existing program synthesis and evolutionary computation literature.

References
Meyerson, E., Nelson, M.J., Bradley, H., Gaier, A., Moradi, A., Hoover, A.K. and Lehman, J., 2024. Language model crossover: Variation through few-shot prompting. ACM Transactions on Evolutionary Learning, 4(4), pp.1-40.

Grishina, A., Liventsev, V., Härmä, A. and Moonen, L., 2025. Fully autonomous programming using iterative multi-agent debugging with large language models. ACM Transactions on Evolutionary Learning, 5(1), pp.1-37.
Wu, X., Wu, S.H., Wu, J., Feng, L. and Tan, K.C., 2024. Evolutionary computation in the era of large language model: Survey and roadmap. IEEE Transactions on Evolutionary Computation.
Ma, Z., Guo, H., Gong, Y.J., Zhang, J. and Tan, K.C., 2025. Toward automated algorithm design: A survey and practical guide to meta-black-box-optimization. IEEE Transactions on Evolutionary Computation.
Hemberg, E., Moskal, S. and O'Reilly, U.M., 2024. Evolving code with a large language model. Genetic Programming and Evolvable Machines, 25(2), p.21.
Schweim, D., Hemberg, E., Sobania, D. and O'Reilly, U.M., 2022, April. Exploiting knowledge from code to guide program search. In European Conference on Genetic Programming (Part of EvoStar) (pp. 262-277). Cham: Springer International Publishing.
Schweim, D., Hemberg, E., Sobania, D., O'Reilly, U.M. and Rothlauf, F., 2021, July. Using knowledge of human-generated code to bias the search in program synthesis with grammatical evolution. In Proceedings of the Genetic and Evolutionary Computation Conference Companion (pp. 331-332).

(Remarks on code availability)
We looked for code. Results are provided with a helpful interface. Prompts to LLM are provided. No code to the method is provided

Referee #4

(Remarks to the Author)
I co-reviewed this manuscript with one of the reviewers who provided the listed reports.

(Remarks on code availability)
There was no code regarding the method available. The method does not seem reproducible. There was code for solutions

that the method found. These solutions were not directly executable

Referee #5

(Remarks to the Author)
Summary
The authors combine an LLM with tree search to generate code that maximizes a quality metric. They test the system on six scientific tasks: scRNA-seq batch integration, COVID-19 forecasting, satellite image segmentation, zebrafish neural activity prediction, time series forecasting, and numerical integration. The breadth of applications could interest the Nature audience.
The benchmark performance and breadth seems compelling, such as in scRNA-seq batch integration, where the system produced 40 methods that beat all existing methods on the OpenProblems benchmark.

Originality and Significance
I am not a domain expert in the specific applications, so findings within those domains are outside my expertise.
From a machine learning perspective, novelty lacks clarity. Several works, most closely AlphaEvolve, FunSearch, and ADAS, share very similar abstractions: maximize a continuous quality metric using sophisticated search algorithms to solve scientifically relevant computational problems. The authors cite these works but do not clearly distinguish their algorithmic contribution. If they argue their algorithm is more performant, I would expect a direct comparison to these prior methods.
Lack of proper baselines also makes it hard to assess significance. Correct baselines must be cost-equivalent where possible. I would like to see the performance difference between Best-of-N sampling with equivalent compute budget and the proposed tree search. If the gap is marginal or absent, it is unclear why one would use a more complicated system instead of i.i.d. samples. Similarly, I would need a Best-of-N baseline that also uses human-designed task-specific priors—only then can we attribute success to the algorithm itself.

Another way to establish significance would be to test on tasks where prior methods are already deployed, such as AlphaEvolve's mathematical constructions, kernel engineering, or job scheduling benchmarks.
If the main contribution is demonstrating new applications for AI rather than algorithmic novelty, this requires different framing with different implications for significance.

Data, Methodology, and Uncertainties
Kaggle competitions and existing benchmarks seem standard and valid. I list suggested improvements about baselines and evaluations below.
Figure 1 states "standard deviation" for error bars, but this is not common practice for reporting uncertainty across trials. I would like the authors to clarify whether this is standard deviation or standard error.
How many times did the authors repeat each experiment, and how often did they discover state-of-the-art? I could not find information about repeated trials in the manuscript. If this is stated, I apologize for missing it.

Conclusions
The authors frame the problem broadly, e.g., solving a large array of problems in empirical sciences. Given this broad framing, the lack of discussion about risks and ethical implications is concerning. Automated systems optimizing evaluation metrics can exploit spurious correlations or perform reward hacking, with unexpected implications. It is unclear how interpretable the generated code is, or whether it could produce dangerous outcomes. There is little evidence that these outputs are safely usable by humans. Scientific discovery itself can be dangerous if not performed with care.
Overall, the lack of extensive discussion on risks is concerning, especially for a potential Nature paper given its broad audience.

Suggested Improvements
Baselines should include cost-equivalent Best-of-N sampling, as well as Best-of-N with human-designed task-specific priors. Without these, we cannot attribute success to the algorithm itself.
A direct comparison on AlphaEvolve tasks (mathematical constructions, kernel engineering, job scheduling) would help clarify the contribution. If existing methods like AlphaEvolve, FunSearch, or ADAS cannot be applied to these tasks, the authors should explain why.
The error bars in Figure 1 need clarification—are they standard deviation or standard error? The manuscript should also report how many times each experiment was repeated and how often the system discovered state-of-the-art.
A systematic accounting of compute costs across all experiments would help assess practicality.
For reproducibility, the authors should release code for the tree search algorithm and evaluations, not just output artifacts. Whether tasks will be released should also be clarified.
Finally, discussion of risks needs expansion—reward hacking, spurious correlations, interpretability of generated code, potential for dangerous outcomes. This is especially important for a Nature paper with broad audience.

Code Availability
I could not find code for the algorithm or evaluations. I found only the output artifacts and LLM-generated summaries at https://github.com/google-research/score/tree/main. It does not appear possible to reproduce results, i.e., run the tree search algorithm and observe its progress.

(Remarks on code availability)
No code is provided for the method, which is a significant concern.

Referee #6

(Remarks to the Author)
I co-reviewed this manuscript with one of the reviewers who provided the listed reports.

(Remarks on code availability)

Referee #7

(Remarks to the Author)
Summary:

This paper introduces a general-purpose tree-search-based method for using LLMs to write software that optimizes a score, i.e., “empirical software”. The tree-search implementation is inspired by the success of systems like AlphaGo. The capabilities of the method are demonstrated across a diverse set of empirical software domains, ranging from machine learning to simulating neurons to solving difficult integrals. As a consequence of its implementation through LLMs, the system is also naturally able to effectively incorporate prior expert ideas (from both humans and AI). Altogether, the paper presents a convincing study of how combining LLMs with search is a powerful technology for writing software whose quality can be effectively measured with a score.

Comments on the COVID prediction experiments:

The approach is a good fit for optimize code for Covid hospitalization prediction, since there are a handful of diverse established approaches originating in different communities, such as epidemiology and time-series prediction, and it makes sense to try to advance and recombine the most effective aspects of each. The tree-structure figure shows pruning and deepening indicative of a healthy search process. Addressing the following points would solidify the results further:

1. The proposed system demonstrates substantial numerical improvements over the CovidHub ensemble, and the qualitative explanations for how the best software developed by TS combines preexisting ideas to discover improvements makes sense. The most helpful additional sanity check for the reader would be to include plots of actual predictions, for example, including a Supplemental Figure of ground truth and predicted hospitalizations for at least the locations and time periods where TS shows the greatest improvement over the CovidHub ensemble. Prediction curves can be shown for ground truth, CovidHub-ensemble, Google Retrospective, and the lowest WIS solution discovered by TS (CEPH-Rtrend_covid x CMU-climate_baseline). The reason these plots would be useful is that in some temporal regimes of COVID simply predicting a low constant value with low variance can lead to significantly improved scores. It would be good to confirm that the system has not simply been lucky to find itself in such a regime. Seeing how exactly the predictions from the AI-written code improve compared to CovidHub ensemble will also generally make the results more impressive: From the scale of the numerical improvements, we should expect to see a visible qualitative improvement in the prediction curves.
2. Please clarify whether the proposed approach in the Covid domain is to run TS every few weeks to get new code or to discover a single piece of code that will generalize across temporal and geographic regimes.
3. There are eight root nodes instead of one in the search tree. Was the root node left out? Or did TS start with the eight baseline solutions here?
4. “Models that perform worse than CovidHub-baseline are not shown.” Please clarify whether this includes solutions produced by TS, or just other models uploaded to CovidHub.
5. Please clarify what the 3-week evaluation period is in Figure 1e. Is this after the 14 search weeks in Figure 1a? Does that help explain why CovidHub-ensemble outperformed Google Retrospective here? Was there evidence in validation that the best TS models in this test period (e.g., CEPH-Rtrend_covid x CMU-climate_baseline) were going to be the best? A Supplementary Table could be added that details the dates, time horizons, and locations used. More generally, since so many different system configurations are tried, it would help to more clearly delineate which scores correspond to validation and which to testing.

Comments on the methodology:

The paper presents a convincing study of the power of combining LLMs with search to write expert-level empirical software, but the particular TS approach used is undermotivated. As is, the main motivation appears to be that TS worked well for AlphaGo and related systems, so why not try something similar here. The comparison to Best of 1000 in the Kaggle Playground confirms the value over random sampling, but not over other non-trivial search methods. Even a small amount of additional articulation of the advantages of TS could go a long way in making the approach feel less arbitrary and more informed by prior work, for example, by clarifying the following points:

1. TS comes out of a now long lineage of methods that combine LLMs with search to optimize code, including FunSearch (as referenced in the Discussion), but originating with methods using LLMs as drop-in operators in genetic programming, i.e. ELM for mutation (https://arxiv.org/abs/2206.08896) and LMX for recombination (https://arxiv.org/abs/2302.12170). TS does not “generalize” FunSearch, as claimed in the Discussion, rather it is a different class of search algorithm with different properties. E.g., one key difference is that the experiments in this paper use orders-or-magnitude fewer evaluations than

those in the FunSearch paper, suggesting one potential advantage of TS is its sample efficiency. On the other hand, the Introduction claims the power comes from the ability of TS to “exhaustively” search, which suggests that if one were to run TS indefinitely, all potentially optimal solutions would be sampled. So, perhaps the advantage of TS is that it is both sample-efficient (as shown empirically across diverse domains) and (in some sense) complete.
2. Another claimed advantage is the ability to recombine existing expert ideas. The approach is clearly inspired by evolutionary methods, but this inspiration is not articulated. The closest in spirit seems to be RHEA (https://arxiv.org/abs/2411.00156), where the comparative advantage of TS is that it recombines expert ideas in conceptual space instead of distilled model space.
3. AIDE is included in Figure 1b but not mentioned in the text. Since this is the only quantitative comparison to prior approaches, it would be helpful to articulate features of TS might make it better than AIDE.
4. Since many readers will have used LLMs to help write software, it would be helpful to somehow compare TS to the most common approach used: Asking the LLM for a solution and then iteratively asking the LLM to improve it. It might be possible to do this comparison without running additional experiments, but instead by analyzing the existing search trees, e.g., what would have happened if we had always expanded the most recent node? What would have happened if we had always expanded the best node so far? Presumably, both of these approaches would lead to much earlier stagnation. This gets back to the “completeness” of TS suggested in (1): It provides an elegant and practical way to get unstuck.

Minor Comments:

1. How was the c_puct parameter tuned? A Supplementary Table with the c_puct used for each experiment would make the methodology more tangible and concrete. Ideally, the caption would include a description of how the values were tuned.
2. For replicability, it would be helpful to include a Supplementary Table listing the Kaggle competitions included in the Kaggle Playground experiments.
3. Clarify use of TS in the following: “In total, 6/11 base methods...” -> “After TS was applied to each, 6/11 base methods…”
4. “We allowed library to have” -> “We allowed the library to have”
5. I like that the hybrid prompt asks for something “TRULY WONDERFUL”.

(Remarks on code availability)
The code produced by the system is provided for several experimental runs. The interface for exploring this code is useful, particularly in how it shows the diff from parent to child. Evaluation harnesses for running the AI-written code are not provided, but presumably would be not-too-difficult to replicate, since these are standard benchmarks. The code for tree search is not provided, but the algorithm is clear enough to reproduce from the pseudocode.

Referee #8

(Remarks to the Author)
I co-reviewed this manuscript with one of the reviewers who provided the listed reports.

(Remarks on code availability)
Code link contains documentation but not runnable source files.

Referee #9

(Remarks to the Author)
The authors implement an AI system for creating expert-level scientific software to maximize a given metric. Focusing on the scRNAseq batch integration method, the AI system does indeed appear to develop improved methods for batch integration. However, the best method remains an implementation of an existing method, that combines it with the top prior method. The gains are minimal for most models better than the prior best and are modest for the top model. It would be more effective to demonstrate a new method that is not a recombination of others but a completely different approach that significantly outperforms the prior alternatives. That being said, the AI system does accomplish the stated goal of improving the metric as directed.

The choice of bioinformatics task of batch integration was likely due to the existence of a benchmark that is straightforward to score. This is a preprocessing task though and not one that drives new biological insights, and the benchmark is affected by issues including the fact cell types are annotated based on clusters derived other models and not a clear ground truth, that batch integration in CELLxGENE data is likely removing true biological signal if integrating across patients and other biological features rather than, for example, the same sample sequenced side by side on different platforms. However, these are issues with the task and benchmark in general. Overall, the AI system is simply improving the metric it was directed to improve, and it does appear to accomplish that, albeit without the most creative approach, rather by recombining prior methods.

(Remarks on code availability)

Version 1:

Reviewer comments:

Referee #1

(Remarks to the Author)
We thank the authors for their detailed rebuttal and the valuable additions to the manuscript. The inclusion of the pseudo-code and the new section on designing scoring metrics bring much-needed clarity to the optimization mechanics and the practical application of the framework. However, the overarching claim that the system "solves scientific problems" still receives only limited evidence. While we appreciate the updated figure caption and the references to recent preprints, citing non-peer-reviewed work is not quite sufficient to validate the broad claims made in this manuscript.

Regarding the evaluation on the GIFT-Eval benchmark, we understand that the research was conducted prior to the release of several newer baselines. However, retaining the claim in the abstract that the method produces "state-of-the-art" software for time series forecasting is highly problematic given the current research landscape. Because the evaluation relies on an older snapshot and omits standard probabilistic metrics like the Continuous Ranked Probability Score (CRPS), the SOTA claim cannot be comfortably maintained. We strongly recommend either removing this specific SOTA claim or updating the evaluation to reflect current standards and baselines.

While the proposed method represents a genuinely strong and innovative approach to empirical software optimization, the persistence of these fundamental issues prevents us from recommending the manuscript in its current form. The disconnect between the empirical evidence and the conceptual framing of "solving science," combined with the unsupported SOTA claims in the abstract, remain critical barriers. Consequently, these unresolved concerns lead us to decline the current submission.

(Remarks on code availability)
The results are not reproducible, as non-publicly-available imports are performed, and usage instructions and examples are missing. The submitted code is also very short and the comments state that it is a "Generic implementation"; I therefore wonder how it relates to the code actually used to obtain the experimental results.

Referee #2

(Remarks to the Author)
I co-reviewed this manuscript with one of the reviewers who provided the listed reports.

(Remarks on code availability)

Referee #4

(Remarks to the Author)
I co-reviewed this manuscript with one of the reviewers who provided the listed reports.

(Remarks on code availability)
There was a basic example and a README file. The code was easy to run and understand for the basic example. There was one minor issue for saving the output. Could not see `futz_progress.json` file initial, but then I edited line 252 in playground_s3e1.py to `/results`
A general problem is that the use of proprietary LLMs make it difficult to claim reproducibility without access to all the prompts and settings used for LLLs.

Referee #6

(Remarks to the Author)
I co-reviewed this manuscript with one of the reviewers who provided the listed reports.

(Remarks on code availability)
I remain extremely concerned about the code that was provided, if I am not missing anything. Please feel free to correct me for what I say below, but I tried to look hard at the provided code.

The implementation describes itself as a "Generic implementation of the Flat Upper Confidence Bound (UCB) Tree Search algorithm" and contains none of the implementation details that would actually be needed to reproduce the results in the paper. A generic UCB implementation is not what was requested, generic UCB code is freely available and reproducing it adds nothing.
Likewise, the accompanying experiment notebook appears to demonstrate single-prompt LLM sampling, which is similarly trivial and ubiquitous. What is missing is precisely the substance of the system: the prompt construction pipeline, the integration between the search loop and the LLM, the sandboxing and scoring infrastructure, the recombination machinery, the per-task prompt templates and configurations, and the harnesses used for each of the six benchmarks. Without these components, the released code does not enable reproduction of any experiment in the paper, nor does it allow practitioners to apply the method to new problems, which were the two motivations behind the original code availability request.
Given that this is a Nature submission and that the authors had the opportunity to address this concern during revision, I find the current release inadequate. I would ask the authors to release the full system used to produce the reported results, or to provide a clear explanation of which specific components cannot be released and why.

Referee #7

(Remarks to the Author)
Comments on requested updates:

Thank you to the authors for providing the requested additional details and updates. These go a long way in making the paper clearer and more concrete.

The extended plot including the 9 TS models and 3 from other submissions that were not better than the baseline would be great to include as a Supplemental Figure, as would the validation performance plot corresponding to the test performance plot in Figure 3e. Both are useful for concluding the results are meaningful.

The plots visualizing the actual predictions from different models are especially helpful in making the contribution concrete. However, since there are only a handful of prediction points on each curve, it is not immediately obvious to the reader why certain curves are better than others, so it would be very helpful to include in the caption of the figures a qualitative description of why the predictions from TS are better than the Ensemble, e.g., the middle column predictions are apparently linear, but are a very effective linear predictor, while the Retrospective predictions are relatively flat but with a small dip in the middle, and the clearest advantage of the TS methods are the narrower confidence bands that still usually contain the ground truth. This is the main behavior I see; I may have missed other qualitative advantages of the TS predictions.

Thank you for mentioning AIDE in the main text. Please provide a citation there and mention what it is, e.g., "… AIDE^cite, a prior LLM-based tree-search method,..."

The author response notes that RHEA will be mentioned in the discussion, but it is not in the revised version of the paper.

Comments on framing:

The main consensus across reviewers seems to be that the paper offers valuable contributions, but the framing is not well-aligned with the contributions. The authors appear to agree with the framing concerns and that the framing can be clarified, but the paper text has not yet been updated to match.

Would the authors be willing to update the framing? This would be especially meaningful in the abstract and introduction.

For example, based on the reviews and author responses, an updated framing could be something like:

1. Writing scientific software is hard and time-consuming.
2. Recently, LLMs have shown the ability to improve code whose quality is "scorable".
3. Inspired by this ability and the success of AlphaGo/AlphaZero, this paper introduces a general system that combines LLMs with tree search to write and improve scientific software.
4. The system makes meaningful improvements to scientific software across a diverse range of important scientific problems, including…
5. The system is able to effectively integrate expert ideas, and the validity and value of the improvements in each domain have each been verified by domain experts.
6. The results constitute the first demonstration of a system with the capacity to improve high-quality scientific software in general, thus representing a significant step in accelerating scientific progress.

It doesn't need to be exactly this, but a framing like this makes it clear that, as the authors note, the main contribution not algorithmic, but rather about presenting a broad convincing study whose value is supported by expert scientists from diverse fields.

(Remarks on code availability)
The code provided looks sufficient for implementing the algorithm and applying it to new applications. I did not try running it. Ideally, the code would also come with complete instructions for how to apply it to one or more of the applications in the paper, including complete prompts, so that future researchers can confirm their experiments are set up correctly.

Referee #8

(Remarks to the Author)
I co-reviewed this manuscript with one of the reviewers who provided the listed reports.

(Remarks on code availability)

Open Access This Peer Review File is licensed under a Creative Commons Attribution 4.0 International License, which permits use, sharing, adaptation, distribution and reproduction in any medium or format, as long as you give appropriate credit to the original author(s) and the source, provide a link to the Creative Commons license, and indicate if changes were made.
In cases where reviewers are anonymous, credit should be given to 'Anonymous Referee' and the source.
The images or other third party material in this Peer Review File are included in the article's Creative Commons license, unless indicated otherwise in a credit line to the material. If material is not included in the article's Creative Commons license and your intended use is not permitted by statutory regulation or exceeds the permitted use, you will need to obtain permission directly from the copyright holder.
To view a copy of this license, visit https://creativecommons.org/licenses/by/4.0/

Feb 23, 2026

Dear Yann,

Thank you again for the very helpful referee reports on our paper, *An AI system to help scientists write expert-level empirical software.* We are very grateful to you and the referees for their detailed and helpful feedback, which has allowed us to substantially improve the manuscript. In the attached manuscript file, we have addressed the comments.
This version also includes responses to the domain expert Referee 7, who provided very useful comments on both the Covid example and the general method.

Modulo small wording changes, the changes to the manuscript are colored with red text.

We have also developed an implementation of the core algorithm to open source with the publication and are including this here. For now in this resubmission, we are including both the core algorithm and tests of it; this can easily be hooked up to sandbox and problem evaluation, and we are open to advice about how this should be done for the publication.

Although I know you haven't yet heard back from the domain reviewers, I thought it prudent to send this back to you at this point by email. This field is moving quickly.

Thanks so much

Michael Brenner

Referee #1:

Remarks to the Author:
Summary

The authors propose an AI-based system that autonomously generates and optimizes scientific software. The approach combines a large language model with Monte Carlo Tree Search (MCTS) to iteratively improve empirical software according to a measurable performance score. The authors define empirical software as code designed to solve “scorable tasks,” i.e., problems where quantitative evaluation metrics are available.

By framing software creation as a search over programs that maximize such metrics, the system is able to explore, refine, and evaluate candidate solutions in an empirically grounded fashion. The method aims to accelerate the development of high-quality computational tools in scientific domains where empirical experimentation is typically time-consuming and limited by human effort.

The proposed methods generate multiple candidate code solutions and apply MCTS to determine which candidates merit further exploration. Each node in the search tree corresponds to a code variant, and expansion decisions are guided by a PUCT selection rule that balances exploration and exploitation, which is an adaptation of the Upper Confidence Bound (UCB) used in traditional MCTS (as used in AlphaZero). Code generation itself follows an iterative mutation process, where an LLM rewrites existing code to improve the measured score. This process is augmented by the injection of “research ideas,” drawn from external sources such as academic papers, textbooks, or search results, which can either be automatically retrieved or manually provided by the user. In this way, the system integrates domain knowledge into the search loop.

The system was initially refined on a curated set of Kaggle playground competitions, which served as a benchmark environment. Finally the authors evaluated their approach across six scorable tasks drawn from diverse scientific domains, including genomics, neuroscience, public health, geospatial analysis, time-series forecasting, and numerical analysis. Task selection followed three criteria: (i) areas where recent methodological progress has been slow, (ii) relevance to active scientific questions, and (iii) direct usefulness to the research agenda of at least one co-author. This selection aims to demonstrate both the breadth of applicability and the practical scientific value of the proposed method.

Strengths

The paper is well motivated, drawing on the success of MCTS in accelerating complex search and optimization problems across domains such as games, planning, and automated reasoning. Adapting this paradigm to the creation of scientific software is a natural and innovative

extension, as it provides a principled way to explore vast program spaces where direct optimization is infeasible.

The authors identify a timely and relevant problem: writing and refining scientific software is often slow, manual, and domain-specific, creating a significant bottleneck for research progress. Addressing this challenge through an automated, performance-guided framework is conceptually sound and could be practically valuable to practitioners of numerous scientific fields.

The methods generated for the scientific tasks appear to be immediately relevant to a wide range of scientific disciplines. In general, fields that rely on computational experimentation could benefit from methods that accelerate software development and optimization while maintaining domain-specific rigor.

A notable strength of the method is its capacity to incorporate human knowledge. Researchers can inject domain insights, hypotheses, or literature-based ideas into the search process, enabling a hybrid workflow that combines human expertise with large-scale automated exploration. This positions the work as a step towards a human–machine collaborative research paradigm, accelerating scientific progress in various fields.

The outcome of the optimization process is executable code, which is generally interpretable and verifiable by human experts. This transparency distinguishes the method from many black-box AI systems and contributes to its credibility and potential for adoption in scientific contexts.

The method proves to be competitive across the diverse set of benchmarks selected by the authors, spanning areas such as genomics, public health forecasting, and geospatial analysis. This breadth of applicability underscores the generality of the approach, which is particularly relevant given its intended role as a tool to support and accelerate research across multiple scientific domains.

The authors also provide an interactive visualization interface that makes the optimization process and intermediate solutions transparent. The visual summaries of score improvements and tree structures substantially improve explainability and facilitate critical assessment by users.

The datasets and benchmark sources used for evaluation are publicly accessible, enabling independent verification and reproducibility of the reported results. The authors' clear documentation and citation of data sources further strengthen the work's transparency and scientific robustness.

RESPONSE: Thank you for the positive comments on the manuscript and how it was presented, we really appreciate it.

Weaknesses

Solving scientific problems in the strict sense involves reasoning about theory, mechanisms, or mathematical frameworks. These are tasks that humans can perform with limited data and without relying on predictive modeling. The system presented in this paper, with its iterative search guided by external research ideas, could in principle support this kind of higher-level reasoning. However, the chosen benchmarks do not reflect this potential. Five of the six evaluated tasks are predictive modeling problems, and only the numerical integration example approaches scientific inference, albeit in a computational sense. As a result, the evaluation primarily demonstrates that the method is capable of engineering and optimization performance rather than genuine scientific discovery.

The paper tends to overstate the point of scientific discovery by implying that the methodology “solves scientific problems,” although the demonstrated applications remain within the realm of performing predictive modelling in the context of machine learning. While the distinction between conducting science and supporting scientific work is stated clearly in the abstract and introduction, it becomes less precise later on (for example, in the caption of Figure 1 and in several passages on pages 15–17). Reframing such statements to emphasize that the system automates empirical software development in service of scientific inquiry, rather than performing scientific reasoning itself, would improve conceptual clarity and align the claims more closely with the evidence.

RESPONSE: We appreciate the reviewer’s point regarding the definition of 'genuine scientific discovery.' Indeed, as stated, the evaluated benchmarks are primarily predictive/optimization tasks, while the text of our paper does hypothesize that our system, 'with its iterative search guided by external research ideas, could in principle support this kind of higher-level reasoning' .

We formulated the paper around the need to rigorously benchmark the ability to solve scientific problems against human performance -- which led us to focus on scientific tasks that are directly graded against humans. Yet, as the reviewer observes, the technology is not limited to such problems, and indeed, we have worked out a large number of other examples.

We agree it is useful at this point to highlight a few of these explicitly. Since our initial submission of this manuscript, our core algorithm has been deployed on new tasks that map onto the reviewer's three criteria for genuine discovery. We have added a new paragraph to the Discussion ('Beyond Predictive Modeling: Toward Mechanistic and Theoretical Discovery') detailing these advances:

1. **Mathematical Frameworks:** Directly addressing the reviewer's criterion of reasoning 'without relying on predictive modeling' or 'with limited data', the system was deployed in theoretical astrophysics to derive exact analytical spectra for cosmic strings. This example has no empirical data, instead the system utilized

pure symbolic mathematical reasoning to discover a closed-form analytical solution to an integral that previous human efforts had only solved asymptotically. The scorable task corresponded to requiring that the analytical solution, expressed in python, matched numerical calculations for random parameters. (Posted as part of http://arxiv.org/abs/2602.03837, with a longer paper to be posted soon).

2. **Mechanism Discovery in a Neural Circuit:** Tasked with system identification in an in silico zebrafish, the system utilized structural priors (wiring diagrams) to bypass statistical shortcuts and autonomously discover the true underlying mechanisms of the neural circuit. The paper is posted to the arxiv here: http://arxiv.org/abs/2602.04492

These are but a few of the applications that have been subsequently studied that meet these criteria. Another application we are quite proud of is the use of the system to capture the **causal** effect of air traffic on reflected shortwave radiation. This has been an open problem for years and the system was able to solve the problem extremely quickly. This is currently being written up for publication, and although it is premature to include it here as part of this resubmission, we anticipate it will be published and posted soon.

We have added these new theoretical and mechanistic applications to a new section in the Discussion, where we clarify the boundary between what the initial benchmarks test (predictive engineering) and what the system architecture has now empirically proven it can achieve (genuine scientific discovery), citing the papers above. We thank the reviewer for prompting this crucial clarification.

At the same time, we have updated the caption of Figure 1 to more clearly indicate the distinction between empirical software development and scientific reasoning.

The system automates empirical software development by iteratively optimizing predictive models and computational algorithms based on external literature and a defined quality metric. We use LLM summaries of scientific papers or AI assisted literature as part of the prompt, and also recombine successful implementations of ideas to create more powerful methods.

Finally, we want to remark that we believe that the main power of the tool presented here is that it *assists* human reasoning while making scientific discoveries; the problems we are working on were already posed with candidate solutions by the community, letting us evaluate against human performance. But a large part of scientific reasoning is **getting to the question** that is the heart of the matter. We believe this tool will help scientists accelerate this process, but we do not think that it in itself is encompassing all scientific reasoning. We could add this comment to the paper if the referee/editor found it helpful -- but this is slightly out of scope from what we presented.

The code availability is insufficient for reproducing the main results. The authors have open-sourced only the code produced by their method, not the code for the optimization process itself. Therefore it is not possible for the reviewers to reproduce the experiments nor for practitioners and scientists to apply the method to new problems. This is inconsistent with

Nature's reporting standards, which explicitly require authors to make all materials, data, code, and associated protocols available unless restrictions are clearly disclosed. No such disclosure appears in the manuscript or the submission materials. Beyond policy compliance, the absence of the optimization framework significantly limits the practical impact of the work: practitioners cannot verify the approach, extend it to other domains, or benchmark it against alternative systems. (This is not a reason for rejection, only a downside.)

RESPONSE: Thank you for this comment. We are open sourcing a reference implementation of the code with the publication of this manuscript. This contains the code for the core algorithm, hooked up to a reference LLM and execution environment, which enables replication of the paper results.

While the method automates the creation and optimization of code, the scoring of candidate solutions remains the responsibility of the user. The paper does not discuss how a scientist would design or implement such scoring schemes in practice, even though this step is central to applying the method to new domains. A short section or example illustrating this process would make the approach more accessible.

RESPONSE: This is a valuable point. The reviewer is correct that while the system automates the generation and search of solutions, defining the objective (the scoring metric) remains a central responsibility of the user. We agree that our original manuscript elided this step, and that it is critical to elaborate on that aspect for making the method accessible to new domains.

To address this, we have added a dedicated subsection to the Discussion section titled *Designing Scoring Metrics,* where, we explicitly highlight that outside of canonical ML tasks, inventing a scoring metric requires significant scientific creativity. As an example, we detail how this was handled in the newly cited Cosmic Strings theoretical physics deployment (Brenner et al., 2025). Here we had to design a scoring function that measured how closely the AI's proposed analytical solution matched a high-precision numerical solution across a randomized parameter grid.

Furthermore, we highlight the broader philosophical point about scoring functions and the scientific workflow: the scientific process implicitly includes the creation of scoring functions to try to reach a hypothesized goal, and the iteration of such scoring functions when the solution maximizes the metric but fails to meet the true scientific objective.

We thank the reviewer for pointing out this gap.

The discussion of related work is limited. Several recent systems also perform iterative improvement of code based on scorable tasks, including Llamea [1], EoH [2], and ReEvo [3]. Situating the contribution relative to these systems and explicitly acknowledging their similarities and differences would substantially improve the contextual framing of the paper.

RESPONSE: We thank the reviewer for pointing out these highly relevant and important works. We agree that situating our contribution relative to Llamea, EoH, and ReEvo provides better contextual framing, as the field of LLM-based iterative code generation is advancing rapidly.

In our original manuscript, we introduced this concept under the 'Combining LLMs and Search' section using Google DeepMind's FunSearch (Romera-Paredes et al., 2024) as our primary example. Following this suggestion, we have substantially expanded this section, and now group FunSearch with the reviewer's three suggested papers (Llamea, EoH, ReEvo), and concurrent agentic frameworks (AlphaEvolve) into a comprehensive discussion of LLM-driven Evolutionary Algorithms (EAs).

We use this expanded section to explicitly acknowledge the similarities (the iterative, score-based feedback loop) while sharply delineating the algorithmic differences between these EA-based systems and our own.

We highlight two major differences between our approach and previous work:
1. **Search Mechanics:** The baseline EA systems inherently discard historical states that fail to survive generational culling. In contrast, our system generalizes the search process using a persistent Flat UCB Tree Search (PUCT), allowing principled global backtracking without losing historical search branches.
2. **Initialization via Scientific Literature:** Whereas heuristic-based evolutionary systems generally initialize from simple heuristics or heavily constrained code skeletons, our system uniquely incorporates unstructured domain knowledge, seeding the root nodes of the search tree with 'research ideas' synthesized from external literature. This step was critical for enabling us to invent such a large number of new methods.

We would like to point out that the main novelty of this paper in our view is *not* algorithmic, though we believe that the introduction of tree search as a search algorithm is novel in this type of problem. The main contributions are that:

1. This work is, to our knowledge, the first to demonstrate beating human performance on a wide range of relevant and well studied **scientific** problems, from epidemiology to single cell biology to geospatial analysis and applied mathematics. Despite the growing literature, we are not aware of another study that achieves this milestone.
2. Secondly, this paper demonstrates that by prompting our system with the method description from cutting edge papers, our algorithms not only produce code that follows the methods of the paper but they often outperform the performance of those methods on SOTA benchmarks. This has wide implications for scientific practice.
3. Third, we demonstrate that when (method,optimal solutions) are recombined, we in general produce novel methods that are more performant than anything ever published. We emphasize that these results are not in obscure fields or a single application -- this

includes problems like single cell RNA sequence analysis and epidemiological forecasting where there are large communities innovating constantly.

The combination of these results portends a sea change in the way empirical software development will proceed into the future. We believe it is this empirical demonstration across diverse scientific disciplines that will be of broadest interest to the general scientific community. While we do think that the algorithmic differences emphasized here are important to clarify, we want to be sure that our main point is clear.

The manuscript characterizes AutoML as the automation of model and hyperparameter search, which is an oversimplification. Modern AutoML frameworks encompass broader functionality, including feature engineering, meta-learning, pipeline construction, and multi-objective optimization. Moreover, several agentic AutoML systems for automated data science already exist, such as AutoGluon assistant / ML-Zero, AgentK, AutoKaggle, etc. While the authors' system indeed extends these ideas toward general-purpose code generation and literature-informed search, more accurate and nuanced description of AutoML would help clarify how the present work fits into and advances this line of research.

RESPONSE: We have expanded our Related Work section to reflect a broader definition of AutoML (including feature engineering and pipelines) and integrated comparisons to the suggested agentic systems (AutoGluon-Assistant/ML-Zero, AgentK, and AutoKaggle).

The description of the search algorithm lacks sufficient technical detail. The paper replaces the traditional Upper Confidence Bound (UCB) with a "Predictor + Upper Confidence bound applied to trees" (PUCT) strategy, yet this modification is only discussed superficially.

The pseudo-code provided omits crucial components: it remains unclear what the "GenerateAndExecute" function entails, how new nodes are generated, and what exactly constitutes a node (whether it represents a research idea, a piece of code, or both). Based on the examples, mutations appear to occur directly at the code level, but this should be explicitly clarified.

RESPONSE: We are now providing a reference implementation of our core algorithm. When attached to an LLM and a Sandbox, it produces candidates as described in the paper. While the reference implementation itself sorts out the issues raised by the referee, we have also updated the description of the algorithm in the Methods section to clarify both the details of the algorithm, and to emphasize that the mutations occur at the code level -- ideas are part of prompts that produce code, that is then scored and iterated upon.

The baseline comparisons used for the Kaggle benchmark are missing relevant baselines. The "single sample" and "best of 1000 samples" conditions are weak baselines and do not adequately represent the current state of automated data science systems. The external comparison against AIDE is outdated, as more recent frameworks such as Agent K [7], MLZero [8], and AutoKaggle [9] address similar goals and would serve as stronger points of reference. The fact that this is a submission to Nature would otherwise (inaccurately) suggest to readers

that it presents a state-of-the-art solution for Kaggle datasets. This limitation should therefore be stated explicitly.

RESPONSE: The work outlined here was conducted over the time period when all of these papers were written and done quite independently. Our choice of comparison to AIDE is that we implemented it ourselves. The Autokaggle paper has results that are weaker than those we present (their scores correspond to making valid submissions and a complex comprehensive score, without scoring to actual percentile ranks on the Kaggle leaderboard). Similarly MLZero reports sparse results and is much weaker than what we show here.

On the other hand, AgentK -- submitted after we submitted this manuscript -- is much more impressive, with results that qualitatively mirror those that we have obtained. Their Figure 3 shows percentile ranks on the kaggle leaderboard across a wide range of competitions. A detailed comparison, competition by competition, would be quite interesting but is beyond the scope of the current work. Nonetheless we are adding a reference to AgentK together with the conclusion that its performance is impressive

Indeed the point of the current work is ***not to benchmark against Kaggle competitions,*** *but instead* to illustrate the use and potential of our **system,** by benchmarking on Kaggle competitions to solve **real science problems** where we can likewise accurately benchmark against human performance. The results of these are why we sent this to Nature. The question of whether the underlying algorithm is matched by other algorithms is interesting and will be addressed in detail in the future -- against Kaggle competitions but more importantly against science problems at the frontier of research.

Furthermore, the prompt for Kaggle datasets describes a highly specific and quite non-human-like approach of writing an entire new gradient boosting library from scratch. This does not yield a very interpretable solution and does not effectively use the domain knowledge a data scientist would have.

RESPONSE: We see this point differently. The libraries that exist for gradient boosting have specific design choices that were made for performance against the datasets that were studied by the community when these were first put together. Writing from scratch frees the system from this constraint. We verified manually that the resulting algorithms do create gradient boosting algorithms according to known theoretical principles, but by not being constrained to the standard libraries, performance improved. We think it is quite interpretable -- the code is clear, and one can explore parameters that are different in the code from typical libraries. We call the reviewers attention to the tree plots (https://google-research.github.io/score/) accompanying these papers: for each node we include a LLM summary of the change made -- which summarizes quite clearly what happened. Moreover, we provide the code diff itself as well, so a reader/scientist can examine in detail why a solution improved.

The ability of the system to rewrite a specialized library from scratch given a different distribution of data than those where the original libraries were introduced could be quite useful.

The evaluation on the GIFT-Eval benchmark appears outdated relative to the current state of the leaderboard. The paper reports results based on a “snapshot” of the leaderboard from May 18, 2025 that predates several strong recent baselines, including TiRex [4], Toto [5], and Timecopilot [6] (let alone yet newer methods like Chronos 2). Timecopilot, in particular, would provide a highly relevant comparison given its agent-based design, which parallels the authors’ approach. Moreover, the evaluation focuses exclusively on point forecasts and does not include distribution-based metrics, such as the Continuous Ranked Probability Score (CRPS), which are standard in time-series forecasting. Updating the evaluation to include these baselines and probabilistic metrics would give a more accurate and comprehensive picture of the method’s competitiveness. Similar to the Kaggle evaluation, given that this is a submission to Nature, without this comparison, readers would inaccurately assume that this presents a new state-of-the-art solution for time series. Indeed, the abstract falsely states this.

RESPONSE: We thank the reviewer for highlighting the rapid evolution of the GIFT-Eval benchmark. While we acknowledge the emergence of recent baselines like TiRex and Timecopilot, we maintain that our evaluation based on a fixed May 18, 2025 snapshot is the most scientifically rigorous approach for this study. Our reasoning for illustrating the platform's capability with that static snapshot is described in four parts below.

Benchmark Instability and Reproducibility We utilized a fixed snapshot of the GIFT-Eval dataset and leaderboard to ensure stability. The GIFT-Eval protocols underwent significant structural changes after our experiments concluded, including major scoring/dataset corrections on July 24, 2025, and the introduction of an "Agentic" category on August 5, 2025. Later updates on August 25 redefined "Zero-shot" to account for widespread test data leakage in foundation models using the large pre-train dataset (Note: our method does not use the large pre-training data).
To avoid unsound comparisons against baselines developed under these revised protocols, we deliberately chose not to submit to the live public leaderboard, restricting our comparison to the May 18th snapshot to ensure a valid, stable baseline.

Evolution of Gemini The Gemini models driving our Tree Search have evolved significantly since our experiments. We utilized Gemini 2.5 Flash, which allowed us to ensure that the GIFT-Eval test data was not part of the model's training set and verify that the generated code was derived from first principles rather than copied from existing solutions online. Re-running experiments with current models (e.g., Gemini 3.0) introduces the risk that the model has since been trained on open-source GIFT-Eval data or solutions, potentially causing data leakage. Reliance on the snapshot ensures we measure the efficacy of the search method rather than the memorization capabilities of newer foundation models.

Probabilistic Metrics Regarding the request for the Continuous Ranked Probability Score (CRPS), we initially selected Point Forecasts (MASE) because it was the primary leaderboard ranking metric at the time. Unlike deep learning where changing metrics only requires retraining the weights, our method iteratively writes and executes deterministic code in order to discover a

performant forecasting model. We cannot approximate CRPS from this model without making invalid distributional assumptions, so producing probabilistic forecasts would require restarting the computationally expensive search to discover a new model. Moreover, an analysis of the current leaderboard reveals that ranking models by CRPS versus MASE yields nearly identical ranking results, suggesting that MASE remains a robust proxy for expert-level performance given the benchmark's scale.

Utility of the Generated Artifact Finally, our method produced a tangible asset: a standalone, general-purpose forecasting library that requires no external machine learning dependencies. We are planning to release this code to demonstrate its immediate utility on novel forecasting tasks beyond the GIFT-Eval context.

We have updated the methods section for GIFT-Eval to emphasize the reasons for snapshotting the date of the leaderboard.

The paper does not report or discuss the computational budget required for its method, including token usage, runtime, or GPU hours. Such information is essential for full assessment and fair comparison, as search-based LLM optimization can be substantially more resource-intensive than single-shot generation. Discussing the computational cost would significantly improve the credibility of the reported results.

RESPONSE: We thank the reviewer for raising this point. We completely agree that providing a detailed account of the computational budget—including token usage, runtime, and hardware requirements—is essential for transparency, reproducible assessment, and fair comparison against single-shot baselines. To address this, we have extracted the exact inference and execution telemetry from our experiments and added a new part of the methods section that gives the results for representative tree search runs for each of the 6 tasks. This includes a comprehensive new table that breaks down the exact average Request Tokens, Response Tokens, Sandbox Duration (in minutes), and Hardware Type (CPU vs. GPU) per search node across all six evaluated benchmarks. The GPUs used in this study are Nvidia Tesla T4s, noted in the Table.

Minor Comments

In the statistical analysis on page 7 a t-test to compare cosine similarity scores of text embeddings is applied. However, cosine similarities are bounded between -1 and 1 and typically deviate strongly from normality, violating the assumptions of a t-test. Using this test gives a misleading impression of verifiability. A visualization-based approach, such as displaying projections of the embeddings, would more appropriately convey how distinct the code clusters are without relying on questionable statistical assumptions.

RESPONSE: Thank you for this comment. We have supplemented the original visualization referenced on page 7 with a new figure displaying a 2D UMAP projection of the text embeddings representing the tree search-generated methods for the single-cell

batch integration task. The new figure shows that the projection clearly reveals distinct spatial clusters. To verify the algorithmic meaning of these clusters, we used Gemini to classify the raw code associated with each point. We found that the UMAP separates non-deep-learning methods (the blue cluster) from deep-learning-based methods (the green cluster).

The associated figure is shown here:

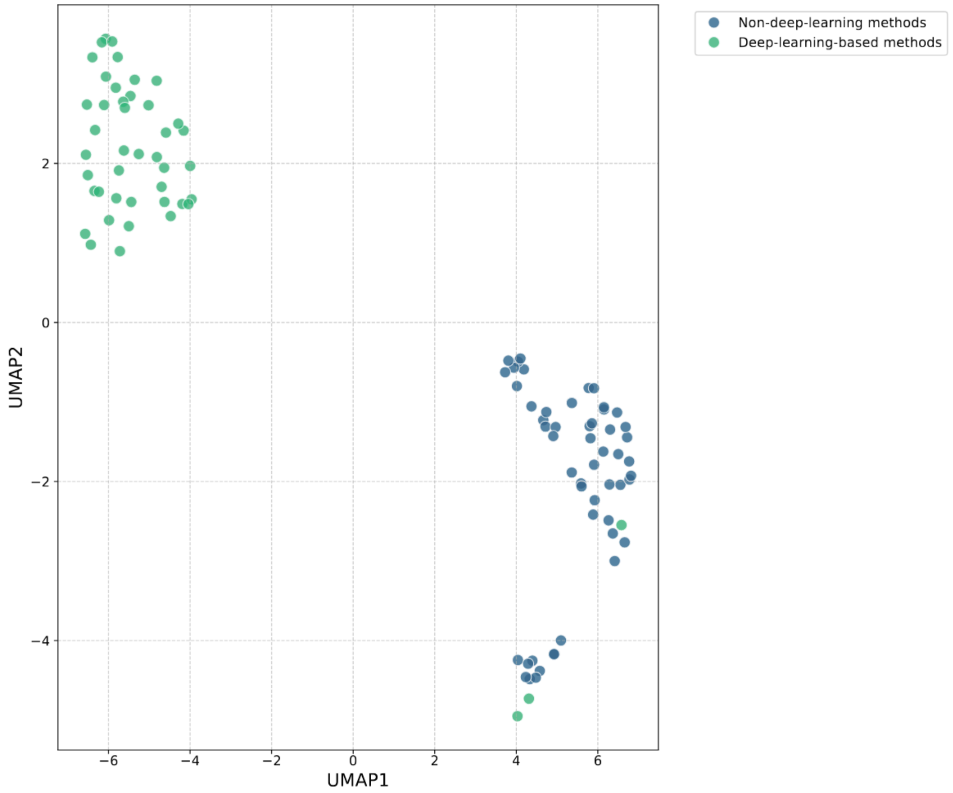

Supplementary Fig. 9 | **UMAP of text embeddings representing tree search-generated methods for single-cell batch integration.** The UMAP shows two major clusters: non-deep-learning methods (blue) and deep-learning-based methods (green). We confirmed this clustering by using Gemini to classify the code associated with each method.

On page 2 ("Overview of Scorable Tasks"), the manuscript states that tasks were selected "using two criteria," yet three are subsequently listed.

RESPONSE: Reworded. Thanks.

Figure 1 could be improved for coherence. Panel 1b presents empirical results, which feels inconsistent alongside Panels 1a and 1c that explain the methodology. Relocating 1b to the Results section would make the figure more focused.

RESPONSE: We understand the referee's point here but this choice was made after much internal debate, and we would prefer to keep it as is. The reason is that our point in showing these results is to explain the logic of how the code mutation system was built: a number of the authors spent 6 months hill climbing against a set of Kaggle competitions, and the major algorithmic choices were made to optimize performance. Thus, from this perspective, Fig. 1b is actually part of the methodology. Additionally, we are actually not showing our best results on Kaggle in this manuscript -- these require, for example, ensembling models from tree search, and in context learning from related competitions. The point of this paper and the reason why we sent it to *Nature* is because the resulting system achieves state of the art performance on *scientific* problems.

Panel 1a could be visually refined for clarity, and Supplementary Figure 22, which offers a clearer depiction of the workflow, might be better suited for inclusion in the main text.

RESPONSE: The referee is making a perceptive point: Indeed Supplementary Fig. 22 was originally our draft for Fig. 1a. Yet after much deliberation, the authors decided that Fig 1a was simpler to understand for most readers and we pushed Supp Fig 22 to the methods section. If the editor wants us to shift this we can, but for now we would prefer to leave it.

Several formatting and terminology issues could be addressed. Some hyperlinks on page 19 cross page boundaries, and a few figures and supplementary sections refer to "agents," whereas the main text consistently uses "LLM." For readers unfamiliar with agent-based terminology, this inconsistency can be confusing. The term "agent," which does accurately describe the system's behavior (e.g., page 4: "the agent discovers strategies"), should be explicitly introduced and defined when first used.

RESPONSE: Thanks for these comments. We have gotten rid of the references to "agents" that the referee brings up, and fixed the hyperlinks.

Recommendation

While we believe that the current submission does not pass the bar of Nature, we invite the authors to resubmit after major modifications.

Areas Outside Reviewer Expertise

The scientific fields of public health, neuroscience, and cell biology fall outside the reviewers' area of expertise. The review therefore does not assess the domain-specific validity of the corresponding benchmark results.

Sources

[1] van Stein, Niki, and Thomas Bäck. "Llamea: A large language model evolutionary algorithm for automatically generating metaheuristics." IEEE Transactions on Evolutionary Computation (2024).
[2] Liu, Fei, et al. "Evolution of heuristics: towards efficient automatic algorithm design using large language model." Proceedings of the 41st International Conference on Machine Learning. 2024.
[3] Ye, Haoran, et al. "Reevo: Large language models as hyper-heuristics with reflective evolution." Advances in neural information processing systems 37 (2024): 43571-43608.
[4] Auer, Andreas, et al. "TiRex: Zero-Shot Forecasting Across Long and Short Horizons with Enhanced In-Context Learning." arXiv preprint arXiv:2505.23719 (2025).
[5] Cohen, Ben, et al. "This Time is Different: An Observability Perspective on Time Series Foundation Models." arXiv preprint arXiv:2505.14766 (2025).
[6] Garza, Azul, and Reneé Rosillo. "TimeCopilot." arXiv preprint arXiv:2509.00616 (2025).
[7] Bou-Ammar, Haitham, et al. "Kolb-Based Experiential Learning for Generalist Agents with Human-Level Kaggle Data Science Performance." (2025).
[8] Fang, Haoyang, et al. "Mlzero: A multi-agent system for end-to-end machine learning automation." arXiv preprint arXiv:2505.13941 (2025).
[9] Li, Ziming, et al. "AutoKaggle: A Multi-Agent Framework for Autonomous Data Science Competitions." ICLR 2025 Third Workshop on Deep Learning for Code.
Remarks on code availability: Please see the main review for comments on code availability.

Referee #3:
Remarks to the Author:
Summary and Main Claims
The paper proposes using large language models combined with tree search (LLM+TS) to rapidly improve existing scientific software methods across multiple domains. The core approach involves searching the program space by mutating existing programs guided by domain information. The central claim is that this represents a fast and accurate method for software optimization that can be applied to hard coding problems across diverse domains.

Strengths
The paper tackles genuinely difficult coding problems, and the ability to solve them is impressive. The integration of program synthesis with 'initial idea search" is novel, while related to a lot of prior work in Genetic Programming (GP) before LLMs that integrated domain knowledge. Two examples: GP used grammars and program metrics (Schweim, 2021) (Schweim, 2022). It appears also, given the (slightly vague) description provided here, to be somewhat like GP approaches with LLMs that use the LLM to initialize a population of solutions. In these approaches, the bias of the model (arising from pre-training) is an implicit form of domain knowledge. Additionally, the tree-based guidance mechanism for search is a novel contribution, though it requires clearer exposition.

RESPONSE: Thank you for these comments, and for providing such an excellent, historically grounded perspective on program synthesis. We completely agree that our 'initial idea search' shares deep conceptual roots with the Genetic Programming (GP) and Evolutionary Computation (EC) communities' long-standing efforts to integrate domain knowledge into search. We have included these excellent references into our GP discussion, including the modern LLM-GP hybrids (Meyerson et al., 2024; Hemberg et al., 2024; Grishina et al., 2025; Wu et al., 2024; Ma et al., 2025).

Finally, the reviewer noted that our novel tree-based guidance mechanism required clearer exposition. We completely agree; the initial description was slightly vague. Prompted by this (and similar feedback from another reviewer), we have completely rewritten the algorithmic exposition. We added a formal 'Search Algorithm Mechanics: Flat UCB Tree Search' section to the Methods, which explicitly defines the global tree structure, the PUCT acquisition function, and mathematically details exactly how the LLM navigates the search space. We believe this thoroughly addresses the reviewer's concern regarding clarity.

The breadth of the results is commendable, with some sort of optimization or further investigation performed within each problem domain. It spans multiple scientific domains demonstrating the generalizability of the approach.

RESPONSE: Thank you for these comments

Weaknesses
The paper does not present a clear research question, prior hypotheses, or conclusions drawn systematically from experimentation. There is minimal discussion of failure cases or conditions under which the method struggles.

RESPONSE: We agree that discussing limitations is crucial. We have added a 'Limitations and Failure Modes' section to the Discussion detailing where the system struggles (e.g., search saturation and the risk of reward hacking if the scorable metric is misaligned).

We would argue that the entire paper is the result of a research hypothesis -- which is that LLMs, together with ideas from the literature, and scorable tasks, make it possible to search through solutions reaching state of the art performance in different fields. One thing we would emphasize is that we did not cherry pick the examples: we have continued to use the system in diverse fields and it consistently performs at the state of the art of human performance.

The paper provides no description of computational costs and resources consumed during the search process. Standard practice in the GP and related evolutionary computation literature requires explicit reporting of the number of fitness evaluations, hardware specifications, model details, computational duration, and financial cost. This omission makes it impossible to assess the practical feasibility or efficiency of the method. It make replicability impossible.

RESPONSE: We thank the reviewer for raising this point. We completely agree that providing a detailed account of the computational budget—including token usage, runtime, and hardware requirements—is essential for transparency, reproducible assessment, and fair comparison against single-shot baselines. To address this, we have extracted the exact inference and execution telemetry from our experiments and added a new part of the methods section that gives the results for representative tree search runs for each of the 6 tasks. This includes a comprehensive new table that breaks down the exact average Request Tokens, Response Tokens, Sandbox Duration (in minutes), and Hardware Type (CPU vs. GPU) per search node across all six evaluated benchmarks. The GPUs are Nvidia Tesla T4s, now noted in the table.

The work lacks essential elements of rigorous empirical evaluation: (1) replicability details including specific models, API versions, and implementation details; (2) robustness assessment through multiple runs with statistical analysis; (3) ablation studies to identify which components contribute to success; (4) sensitivity analysis across different LLM families, versions, and costs; and (5) comparison with alternative methods. The method's sensitivity to hyperparameters (e.g., tree search size, node evaluation time) is not discussed. For example, Figure 1b does not make it clear whether there are statistically significant differences between the methods. For example, the results conflate improvements from algorithmic innovation with performance gains from software optimization. A breakdown is needed: how much speedup came from discovering faster algorithms versus optimizing implementation details? Given a fixed computational budget, did faster algorithms enable more evaluations within the search process itself?

RESPONSE: Thank you for these comments.

(1) **Replicability** We now include replicability details, both in giving a reference implementation of the code, how much compute and tokens were used for representative runs. All runs were done with Gemini 2.5 Flash, as stated in the paper. Although we did of course experiment with different models, we found that this was able to achieve the state of the art results of the manuscript, and while better models (eg Gemini 2.5 pro) improved things somewhat they weren't worth the cost. We added a sentence to the methods highlighting this.

(2) **Replication** We do provide replications of the experiments -- carrying out the experiments in triplicate. We found the runs to be highly reproducible, both with respect to variability of the score and also the replicability of the code to follow the method we are prompting for (See Supplementary Table 5 (Single Cell),9 (Covid), where we detail assessments of the code for the replicates) Supplementary Figure 4 shows the relative performance of different methods over both hyperparameters **and** replicates for the biological example. Each experiment in this figure is a dot. Quantitatively score variation from run to run is around 5%.

(3) **Ablation studies:** The algorithm we are presenting here is the most minimal we found that achieves the state of the results across the kaggle benchmark. In the context of the Kaggle competitions, we tried many ideas that did not lead to consistent increase in scores. The present study then takes the *same* configuration from this search and applies it to real scientific problems where we show it outperforms SOTA human performance. For the science problems, we do extensive comparisons of performance with different research ideas in context. These are ablations -- showing how performance changes by running the same system with methodological priors. For example see Supp Fig 6 which shows an ablation over methods, showing how small changes in the framing lead to changes in score.

(4) **Costs:** To explicitly address the computational cost of the system, we have added a highly detailed 'Computational Cost and Resource Utilization' subsection, complete with a new table detailing the exact token usage and GPU/CPU duration per search node for every benchmark. We determined that Gemini 2.5 Flash was sufficient for achieving the results of this manuscript; 2.5 Pro led to slight improvements on these tasks but not enough to make it necessary. We expect these results to translate to other LLMs with similar benchmark performance (via eg. lmsys.org)

(5) **Alternative Methods:** Prompted by this and similar reviewer feedback, we have significantly expanded our Related Work section. We now explicitly compare our system against the leading alternative paradigms—LLM-guided Evolutionary Algorithms (including FunSearch, Llamea, ReEvo, and AlphaEvolve) and macro-level workflow orchestrators (data-to-paper). We clearly delineate how our Flat UCB Tree Search mathematically avoids the generational forgetting inherent in these alternative methods. On the Kaggle benchmark, we explicitly implemented and compared to AIDE, shown in Figure 1. We have also done internal comparisons in Google to AlphaEvolve and the performance of the two is largely similar. Detailed comparison goes beyond the scope of the present work.

(6) **Why higher scores**. The referee asks why the LLM achieves higher scores, was it mathematically superior algorithms or better python code. Our experience is that it is a combination of both -- as can be observed from the breakthrough plots in the supplemental figures and also in our github, the search continues to discover good ideas as it proceeds. On the other hand, it also does improve implementation details. One amusing anecdotal observation is that when the LLM has trouble improving the score, it tries to improve the code.

Descriptions require more clarity. For example:
- The tree-based search mechanism is inadequately described—for example, it is unclear whether it relates to (1+5) evolution strategies, beam search, or standard tree search variants.

RESPONSE: We are now providing a reference implementation of our core algorithm. When attached to an LLM and a Sandbox, it produces candidates as described in the paper. While the reference implementation itself sorts out the issues raised by the referee, we have also updated the description of the algorithm in the Methods section to clarify both the details of the algorithm, including its relation to the methods outlined here.

- "three in parallel" is unexplained.

RESPONSE: By 'three in parallel', we meant that we ran three independent tree search replicas per method to probe replicability. We have updated the terminology in the text to explicitly state 'three independent tree search replicas' to remove any ambiguity.

Figure 1c and its inline explanation are not explanatory enough

RESPONSE: We have increased the detail in this figure caption substantially to improve clarity.

- The differences between what was done during development and training using Kaggle competitions vs what was done during evaluation with the set of scientific coding problems is unclear.

RESPONSE: The system was originally developed and optimized on Kaggle competitions: a part of the author list hill climbed competitive on the Kaggle Benchmark for about 6 months, figuring out which aspects of the system were most important for high performance. The major aspects of the system were then taken on scientific coding problems. There is no major difference in the method or approach.

From an evolutionary computation perspective, the recombination approach appears similar to work by (Meyerson, 2024), but with careful parent selection;.

RESPONSE: Thank you for this reference. We have now included this in the methods section where we discuss recombination.

The claim that software development is slow lacks supporting data or citations.

RESPONSE: We now provide references to two classic studies (Hannay et al, and Sculley et al) citing the slow nature of software development. While the first study Hannay et al. (2009) shows that scientists spend over 30% of their time strictly on software development, the second study (Sculley et al. (2015)) highlights the massive 'hidden technical debt' and engineering bottlenecks in scientific/ML workflows.

While evolved solutions can be inspected, they remain difficult to understand and validate. The generated methods may lack the explainable arguments needed to justify their correctness. This is not considered.

RESPONSE: It's important to distinguish the complexity of the algorithm from the straightforwardness and familiarity of a given result. Because the output of each node is a notebook with clearly delineated, transparent and executable Python code, it can be readily interpreted and checked. Each node is analogous to what might be shown by a student, postdoc, or other researcher in a lab meeting or conference: the problem statement, a description of the approach taken, the code, a measure of the code's fit to the data, and charts/maps/statistics pertinent to the particular problem. As a simple example, if a solution used a simple regression tree, the notebook would contain imports of any package needed to train or test the tree, and the tree itself is created and inspectable by running the code in the notebook. In our experience, in fact, because the notebook must include all package imports and code, it is repeatably executable and individual solutions can easily be shared across researchers and groups than in our usual somewhat ad-hoc workflows. The search system produces code that is precisely interpretable. One can read the code, devise tests and probe it and why it satisfies the conclusions in detail. Even for complex code, LLMs are very skilled at breaking it down into representative parts of the code and even design ablation tests, as demonstrated in the trees of solutions published at https://google-research.github.io/score/

The results presentation becomes formulaic after the initial examples, with reduced depth in later experiments. This suggests either space constraints forced shortcuts, or the approach's novelty diminishes across repetitions. For an evolutionary computation audience, the result—that a model trained on multi-domain examples performs well across those domains when given appropriate guidance—is not surprising.

RESPONSE: The fact that this system is able to achieve SOTA performance across so many tasks was very surprising to us, as was the ability of the system to accurately produce code implementations from papers that perform at or better than the level of the original paper. It has huge implications for the way science is going to be done.

There is no source code for the method. Only code for solutions discovered by the method are provided. The discovered solutions are not possible do run without more configuration and setup.

RESPONSE: We are now providing a reference implementation of our core algorithm. When attached to an LLM and a Sandbox, it produces candidates as described in the paper. While the reference implementation itself sorts out the issues raised by the referee, we have also updated the description of the algorithm in the Methods section to clarify both the details of the algorithm, and to emphasize that the mutations occur at the code level -- ideas are part of prompts that produce code, that is then scored and iterated upon.

Recent work on LLM-enhanced evolutionary algorithms (Wu, 2024), (Ma, 2025), (Hemberg, 2024) and language model variation (Meyerson, 2024), (Grishina, 2025) address related problems and should be more thoroughly contrasted with this approach. The conceptual framing around "empirical software" and "scorable problems" appears to serve as window dressing for what is fundamentally a program search problem with domain-informed initialization.

RESPONSE: Thank you for these references, we have integrated all five of the reviewer's excellent references directly into our Related Work section. We use these citations (Wu, Ma, Hemberg, Meyerson, Grishina) to acknowledge the paradigm of LLM-enhanced evolutionary algorithms and crossover variation. By placing these alongside our explanation of Flat UCB Tree Search, the manuscript now firmly situates our approach within the EC literature and clearly contrasts our search mechanics with generational EAs.

Secondly, regarding the conceptual framing: We respectfully maintain that terms like "empirical software" and "scorable problems" are not intended as algorithmic window dressing, but rather as necessary definitions of the *application domain* our system targets. The vast majority of classical program search (including the excellent LLM-enhanced EAs cited above) is evaluated on highly structured, localized algorithmic tasks (e.g., evolving short mathematical heuristics, combinatorial optimization, or isolated unit tests). In contrast we use the term "empirical software," we are deliberately signaling to the broader scientific and machine learning communities that our search operates at the macro-level of modern scientific workflows. Our system generates end-to-end machine learning pipelines, complex data processing scripts, and full neural network architectures (such as the multi-hour GPU training runs in our ZAPBench task) that depend heavily on external libraries and must be empirically executed against massive datasets.

Recommendation
While the paper demonstrates promising empirical results on challenging problems, it requires substantial revision to meet publication standards. The authors must provide detailed computational accounting, conduct rigorous ablation and sensitivity studies, discuss failure

modes, and clarify the technical contributions relative to existing program synthesis and evolutionary computation literature.

References

Meyerson, E., Nelson, M.J., Bradley, H., Gaier, A., Moradi, A., Hoover, A.K. and Lehman, J., 2024. Language model crossover: Variation through few-shot prompting. ACM Transactions on Evolutionary Learning, 4(4), pp.1-40.

Grishina, A., Liventsev, V., Härmä, A. and Moonen, L., 2025. Fully autonomous programming using iterative multi-agent debugging with large language models. ACM Transactions on Evolutionary Learning, 5(1), pp.1-37.

Wu, X., Wu, S.H., Wu, J., Feng, L. and Tan, K.C., 2024. Evolutionary computation in the era of large language model: Survey and roadmap. IEEE Transactions on Evolutionary Computation.

Ma, Z., Guo, H., Gong, Y.J., Zhang, J. and Tan, K.C., 2025. Toward automated algorithm design: A survey and practical guide to meta-black-box-optimization. IEEE Transactions on Evolutionary Computation.

Hemberg, E., Moskal, S. and O'Reilly, U.M., 2024. Evolving code with a large language model. Genetic Programming and Evolvable Machines, 25(2), p.21.

Schweim, D., Hemberg, E., Sobania, D. and O'Reilly, U.M., 2022, April. Exploiting knowledge from code to guide program search. In European Conference on Genetic Programming (Part of EvoStar) (pp. 262-277). Cham: Springer International Publishing.

Schweim, D., Hemberg, E., Sobania, D., O'Reilly, U.M. and Rothlauf, F., 2021, July. Using knowledge of human-generated code to bias the search in program synthesis with grammatical evolution. In Proceedings of the Genetic and Evolutionary Computation Conference Companion (pp. 331-332).

Remarks on code availability:

We looked for code. Results are provided with a helpful interface. Prompts to LLM are provided. No code to the method is provided

Referee #4:
Remarks to the Author:
I co-reviewed this manuscript with one of the reviewers who provided the listed reports.

Remarks on code availability:
There was no code regarding the method available. The method does not seem reproducible. There was code for solutions that the method found. These solutions were not directly executable

Referee #5:
Remarks to the Author:
Summary
The authors combine an LLM with tree search to generate code that maximizes a quality metric. They test the system on six scientific tasks: scRNA-seq batch integration, COVID-19 forecasting, satellite image segmentation, zebrafish neural activity prediction, time series forecasting, and numerical integration. The breadth of applications could interest the Nature audience.
The benchmark performance and breadth seems compelling, such as in scRNA-seq batch integration, where the system produced 40 methods that beat all existing methods on the OpenProblems benchmark.

Originality and Significance
I am not a domain expert in the specific applications, so findings within those domains are outside my expertise.
From a machine learning perspective, novelty lacks clarity. Several works, most closely AlphaEvolve, FunSearch, and ADAS, share very similar abstractions: maximize a continuous quality metric using sophisticated search algorithms to solve scientifically relevant computational problems. The authors cite these works but do not clearly distinguish their algorithmic contribution. If they argue their algorithm is more performant, I would expect a direct comparison to these prior methods.

RESPONSE: There are a number of algorithms in the literature that use search algorithms to solve scientifically relevant computational problems. There are several aspects of novelty to this work:

1. While the introduction of MCTS/Tree search algorithms is (to our knowledge novel), we do not think the algorithmic innovation is the main point of this manuscript.
2. More significantly, this work is, to our knowledge, the first to demonstrate beating human performance on a wide range of relevant and well studied **scientific** problems, from epidemiology to single cell biology to geospatial analysis and applied mathematics.
3. The second significant contribution of this paper is the demonstration that with prompting method description from cutting edge papers, our algorithms not only produce code that follows the methods of the paper but in general they outperform the performance of those methods on SOTA benchmarks. This has wide implications for scientific practice.
4. The third significant contribution is to demonstrate that when (method,optimal solutions) are recombined, we in general produce novel methods that are more performant than anything ever published. We emphasize that these results are not in obscure fields -- this include problems like single cell RNA sequence analysis and epidemiological forecasting where there are large communities innovating constantly.

The combination of these results portends a sea change in the way empirical software development will proceed into the future. We believe it is this empirical demonstration across diverse scientific disciplines that will be of broadest interest to the general scientific community.

The referee mentions Funsearch, AlphaEvolve and ADAS. Funsearch is an early paper that worked on much more focused algorithmic innovation. AlphaEvolve is its generalization, and more comparable to what we have shown here. The present work and AlphaEvolve were independently developed by different groups at Google and Google-Deepmind; whereas Alpha Evolve was developed primarily for solving mathematics problems where discovery search was central, the present paper focused on data science and scientific computation more broadly. We agree with the referee that the comparison of these algorithms (as well as others which have appeared in the meantime) is of great interest, more research is needed to decide relative performance. Indeed, this is a topic of ongoing research. We have now included this point in the discussion section of the paper, stating

Alphaevolve showed that evolutionary algorithms performed well on a class of discovery problems. In this paper we explore the use of tree Search algorithms. More research is needed to decide on the relative performance of each.

In response to other referee comments, we have now expanded the discussion to compare to a wide class of evolutionary algorithms, in a section entitled *Combining LLMs and Search*, including algorithms that predate AlphaEvolve.

Lack of proper baselines also makes it hard to assess significance. Correct baselines must be cost-equivalent where possible. I would like to see the performance difference between Best-of-N sampling with equivalent compute budget and the proposed tree search. If the gap is marginal or absent, it is unclear why one would use a more complicated system instead of i.i.d. samples. Similarly, I would need a Best-of-N baseline that also uses human-designed task-specific priors—only then can we attribute success to the algorithm itself.

RESPONSE: We have extensively compared Best-of-N baselines with human designed task specific priors to the results of the tree search algorithm, with a significant performance difference. We compared best of N, greedy algorithms, both with or without human designed specific priors. For Kaggle, these were easy to calibrate as there is an extensive database called MetaKaggleCode which has human best solutions for a wide range of kaggle competitions -- so we could take summaries of these solutions as methods and compare the different situations. Tree search with the algorithm presented in the paper offered significant improvements over these baselines. This is intuitive - successive iterations have updated prompts that include the results and output logs from previous iterations, helping the model understand how to refine and improve.

Another way to establish significance would be to test on tasks where prior methods are already deployed, such as AlphaEvolve's mathematical constructions, kernel engineering, or job scheduling benchmarks.
If the main contribution is demonstrating new applications for AI rather than algorithmic novelty, this requires different framing with different implications for significance.

As stated above, we agree with the referee that the comparison of these algorithms is of great interest, and this is a topic of ongoing research. The preliminary results from both the authors of this paper as well as those responsible for AlphaEvolve is that the methods are comparable when baselined appropriately.

Data, Methodology, and Uncertainties
Kaggle competitions and existing benchmarks seem standard and valid. I list suggested improvements about baselines and evaluations below.
Figure 1 states "standard deviation" for error bars, but this is not common practice for reporting uncertainty across trials. I would like the authors to clarify whether this is standard deviation or standard error.

RESPONSE: We apologize, this confusion arises from a confusion in the meaning of the standard deviation, which we have now clarified. The performance in Fig 1 is the average percentile rank across all 16 competitions in the entire Kaggle Benchmark. Thus while in one competition we might score 85 percentile, in another we might score 75 percentile. The standard deviation measures the spread of this distribution. These are not different trials, they are different tasks. The point is that we wanted to design the tree search system to be robust against variation in the problem statement and we chose this set of problems for benchmarking and designing the system.

We have clarified this in the figure caption stating:

Error bars indicate standard deviation of performance between different competitions in the benchmark.

How many times did the authors repeat each experiment, and how often did they discover state-of-the-art? I could not find information about repeated trials in the manuscript. If this is stated, I apologize for missing it.

RESPONSE: Experiments were generally repeated three times. We observed across all of the studies we have done -- the Kaggle benchmark, and each of the six scientific tasks -- that as the size of the tree increases, the best score saturates -- at some point the model cannot improve it any further given the way we are searching. This phenomenon occurred repeatedly over trials. We therefore set the maximum number of nodes between 300-1000.The breakthrough plots in the supplemental figures outline when and why state of the art is reached. As an example this is the breakthrough plot (Supp Fig 21) from the Integrals task. The jumps give places where the score increases discontinuously -- and the labels give the specific idea that leads to the breakthrough. One can observe that the size of the jump decreases with increasing node number, leading to ultimate saturation.

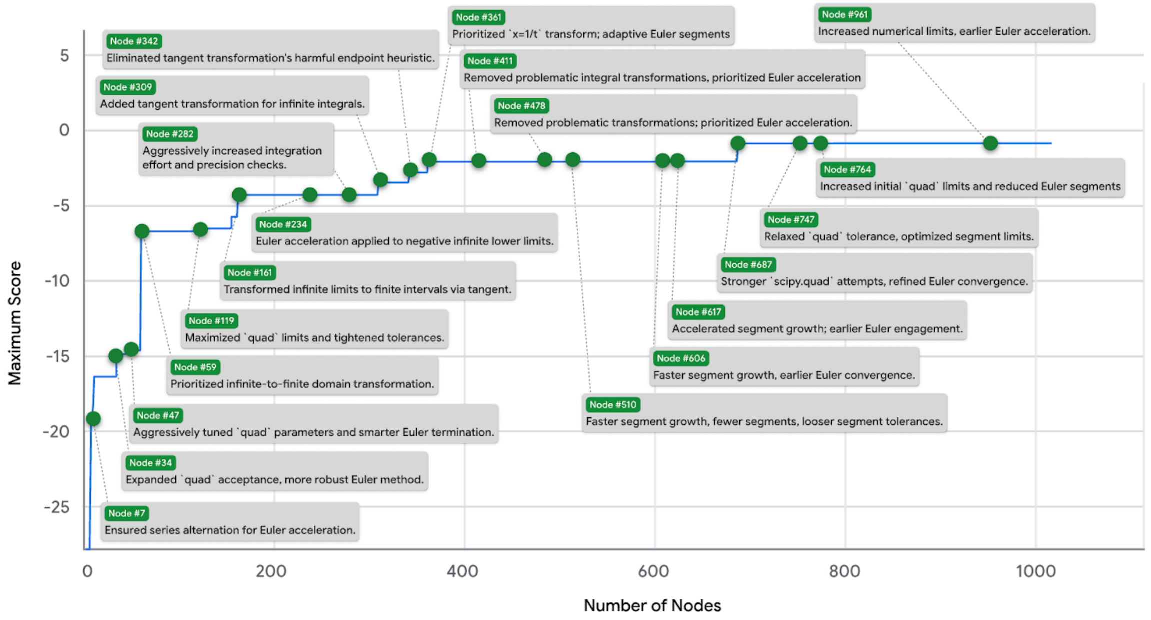

We have added a statement to the Methods section on the saturation of score

With increasing nodes in the tree, the score saturates after 300-1000 nodes (See Breakthrough plots in Supplemental Figures 12,16, 17,etc.)

Conclusions

The authors frame the problem broadly, e.g., solving a large array of problems in empirical sciences. Given this broad framing, the lack of discussion about risks and ethical implications is concerning. Automated systems optimizing evaluation metrics can exploit spurious correlations or perform reward hacking, with unexpected implications. It is unclear how interpretable the generated code is, or whether it could produce dangerous outcomes. There is little evidence that these outputs are safely usable by humans. Scientific discovery itself can be dangerous if not performed with care.

Overall, the lack of extensive discussion on risks is concerning, especially for a potential Nature paper given its broad audience.

RESPONSE: The referee touches on two risks.

1. *Reward hacking.* This is indeed a risk. AI systems can "cheat" and produce code that achieves a high score that is unmerited. The way to combat this risk is that the human must examine the code and understand its correctness and ensure that the scoring meets the stated standards. The code that is produced by the AI system is perfectly interpretable -- every line can be examined to understand how it works and whether it is merited. The authors did this for the examples of the paper. Doing so takes time, but far less time than it would have to create such novel methods in the first place. We also took extensive measures to make sure that the test performance we are reporting cannot be cheated, by not letting the test set performance being reported anywhere close to the

agents carrying out the optimization of code. So, for example, in the batch integration example, our test set was that proposed by the authors of openproblems.bio, orders of magnitude larger and quite different than the dataset that we used to carry out tree search.

2. *Safety.* We agree with the referee that the ability of LLM systems to produce such high quality code does imply safety risks, especially when this code is applied to empirical code that could impact humans in some way. The ability to hit expert level performance across a broad range lowers the required expertise needed to carry out sophisticated technical tasks, which does impact safety. This is a general risk of large inference time compute together with increasing model quality.

In the paper we have added the following section to the discussion:

Safety and the Democratization of Expertise *The ability of LLM-based systems to autonomously produce expert-level empirical software carries broader safety risks, particularly when applied to domains that could directly impact human well-being. By automating complex engineering workflows, our system significantly lowers the technical expertise required to execute sophisticated computational tasks. While this democratization accelerates beneficial scientific discovery, it concurrently lowers the barrier to entry for deploying advanced models in sensitive or potentially dangerous domains. This phenomenon represents a broader, systemic risk associated with the combination of large-scale inference-time compute and exponentially increasing foundation model quality.*

Code Availability
I could not find code for the algorithm or evaluations. I found only the output artifacts and LLM-generated summaries at https://github.com/google-research/score/tree/main. It does not appear possible to reproduce results, i.e., run the tree search algorithm and observe its progress.

RESPONSE: We are now providing a reference implementation of our core algorithm. When attached to an LLM and a Sandbox, it produces candidates as described in the paper. While the reference implementation itself sorts out the issues raised by the referee, we have also updated the description of the algorithm in the Methods section to clarify both the details of the algorithm, and to emphasize that the mutations occur at the code level -- ideas are part of prompts that produce code, that is then scored and iterated upon.

Remarks on code availability:
No code is provided for the method, which is a significant concern.

Referee #6:
Remarks to the Author:
I co-reviewed this manuscript with one of the reviewers who provided the listed reports.

Referee #7 (Remarks to the Author):

Summary:

This paper introduces a general-purpose tree-search-based method for using LLMs to write software that optimizes a score, i.e., “empirical software”. The tree-search implementation is inspired by the success of systems like AlphaGo. The capabilities of the method are demonstrated across a diverse set of empirical software domains, ranging from machine learning to simulating neurons to solving difficult integrals. As a consequence of its implementation through LLMs, the system is also naturally able to effectively incorporate prior expert ideas (from both humans and AI). Altogether, the paper presents a convincing study of how combining LLMs with search is a powerful technology for writing software whose quality can be effectively measured with a score.

RESPONSE: Thank you for these positive comments.

Comments on the COVID prediction experiments:

The approach is a good fit for optimize code for Covid hospitalization prediction, since there are a handful of diverse established approaches originating in different communities, such as epidemiology and time-series prediction, and it makes sense to try to advance and recombine the most effective aspects of each. The tree-structure figure shows pruning and deepening indicative of a healthy search process. Addressing the following points would solidify the results further:

1. The proposed system demonstrates substantial numerical improvements over the CovidHub ensemble, and the qualitative explanations for how the best software developed by TS combines preexisting ideas to discover improvements makes sense. The most helpful additional sanity check for the reader would be to include plots of actual predictions, for example, including a Supplemental Figure of ground truth and predicted hospitalizations for at least the locations and time periods where TS shows the greatest improvement over the CovidHub ensemble. Prediction curves can be shown for ground truth, CovidHub-ensemble, Google Retrospective, and the lowest WIS solution discovered by TS (CEPH-Rtrend_covid x CMU-climate_baseline). The reason these plots would be useful is that in some temporal regimes of COVID simply predicting a low constant value with low variance can lead to significantly improved scores. It would be good to confirm that the system has not simply been lucky to find itself in such a regime. Seeing how exactly the predictions from the AI-written code improve compared to CovidHub ensemble will also generally make the results more impressive: From the scale of the numerical improvements, we should expect to see a visible qualitative improvement in the prediction curves.

RESPONSE: Thank you for this comment. We agree that visual validation is important to confirm that the system is capturing genuine epidemic dynamics rather than exploiting statistical artifacts of low-volatility periods.To address this, we have added two new supplementary figures (also pasted below)

Supplementary Figure 13 plots the ground truth against the CovidHub-ensemble, the Google Retrospective (TS), and our best recombination model across five major jurisdictions (CA, DC, FL, NY, OK). These plots clearly demonstrate that the AI-written code captures the non-linear shifts in hospitalization trends more accurately than the ensemble, even during periods of high volatility.
Supplementary Figure 14 provides a comprehensive view of the entire 2024–2025 season for representative locations, including all validation splits. This visualization confirms that our system maintains superior calibration and predictive power across multiple infection waves and seasonal shift.

Overall, these plots visibly confirm that the numerical improvements achieved by the TS model arise from qualitative advancements in prediction accuracy across diverse temporal and geographic regimes, rather than being confined to low-variance prediction periods.

To clarify the evaluation methodology, the 14 Google Retrospective models were assessed on held-out sets spanning the entire season to demonstrate generalization across time. However, for the primary comparison between the best TS model and the other competing models, we utilized a common held-out set defined by the reference dates 2025-04-05, 2025-04-12, and 2025-04-19. This approach ensures a direct and equitable comparison across all strategies. The actual forecasts for the three suggested models on the common held-out reference dates (2025-04-05, 2025-04-12, and 2025-04-19) are plotted below, alongside full-season plots for the Google Retrospective and CovidHub-ensemble models.

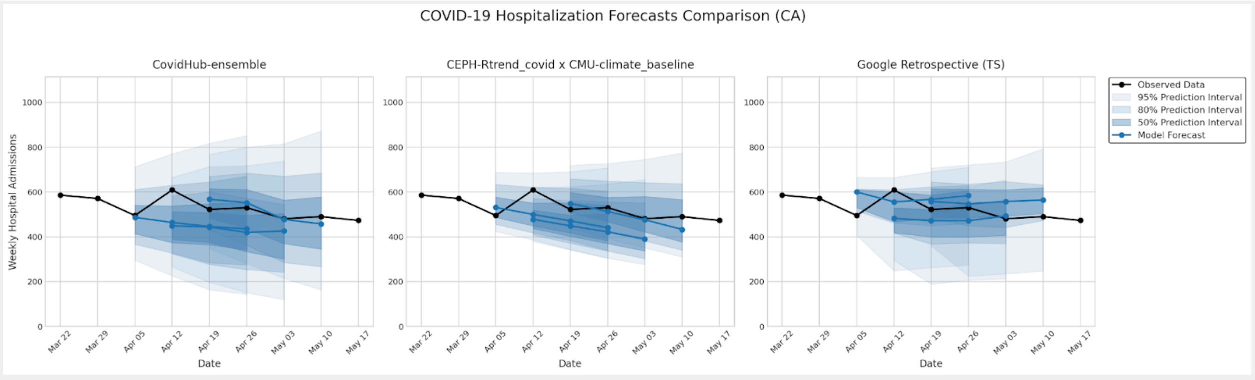

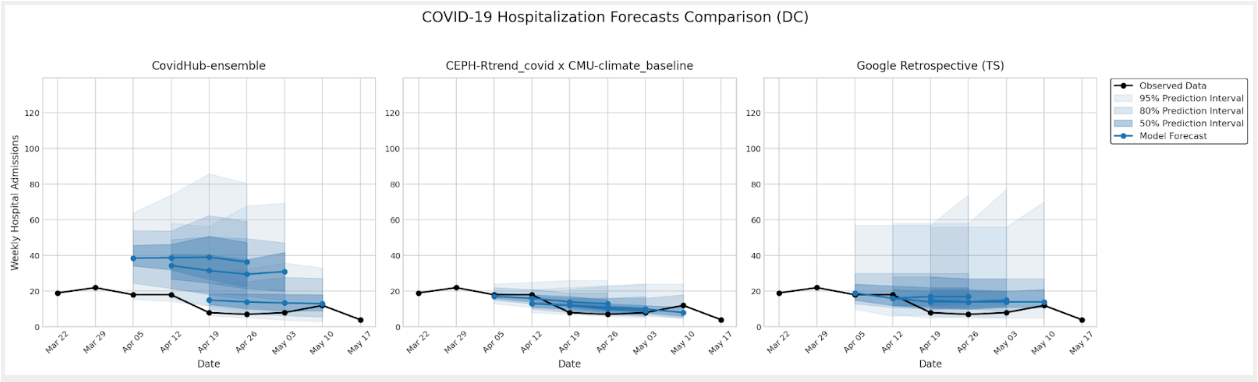
COVID-19 Hospitalization Forecasts Comparison (DC)
CovidHub-ensemble
CEPH-Rtrend_covid x CMU-climate_baseline
Google Retrospective (TS)
Weekly Hospital Admissions
Date
Mar 22
Mar 29
Apr 05
Apr 12
Apr 19
Apr 26
May 03
May 10
May 17
Observed Data
95% Prediction Interval
80% Prediction Interval
50% Prediction Interval
Model Forecast

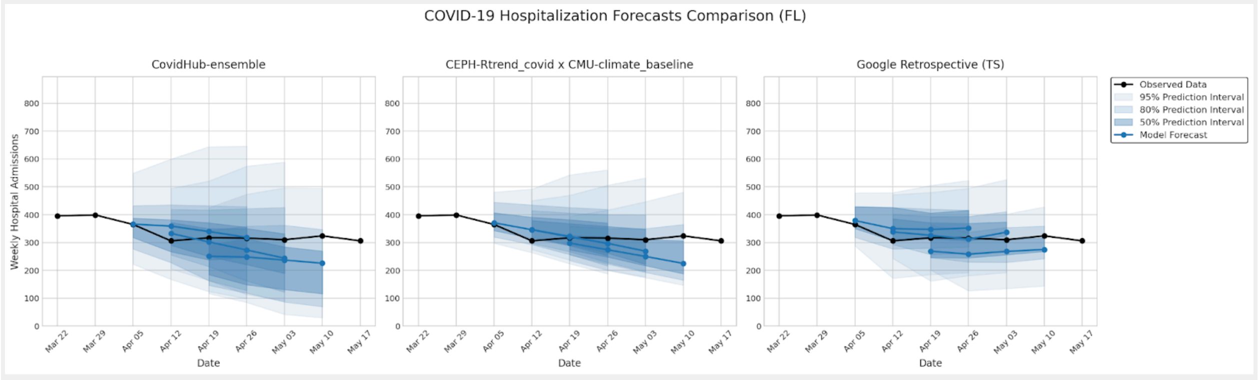
COVID-19 Hospitalization Forecasts Comparison (FL)
CovidHub-ensemble
CEPH-Rtrend_covid x CMU-climate_baseline
Google Retrospective (TS)
Weekly Hospital Admissions
Date
Observed Data
95% Prediction Interval
80% Prediction Interval
50% Prediction Interval
Model Forecast

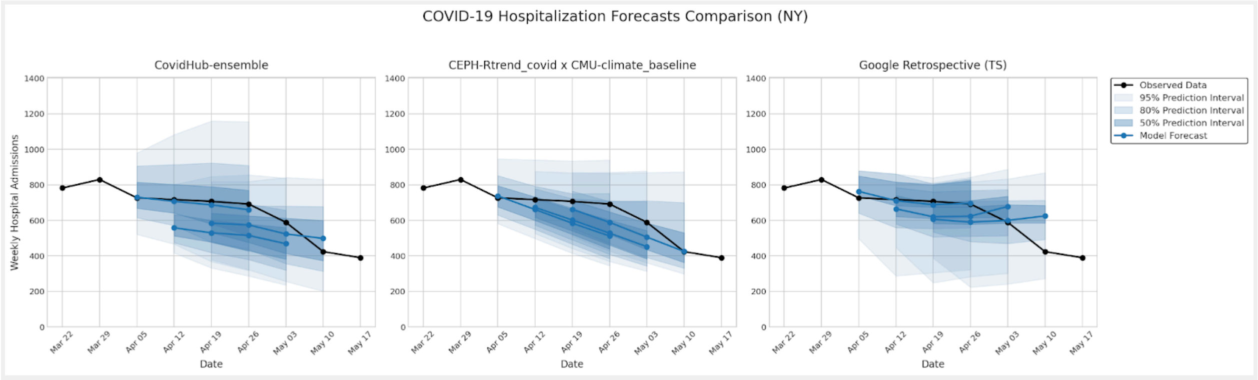
COVID-19 Hospitalization Forecasts Comparison (NY)
CovidHub-ensemble
CEPH-Rtrend_covid x CMU-climate_baseline
Google Retrospective (TS)
Weekly Hospital Admissions
Date
Observed Data
95% Prediction Interval
80% Prediction Interval
50% Prediction Interval
Model Forecast

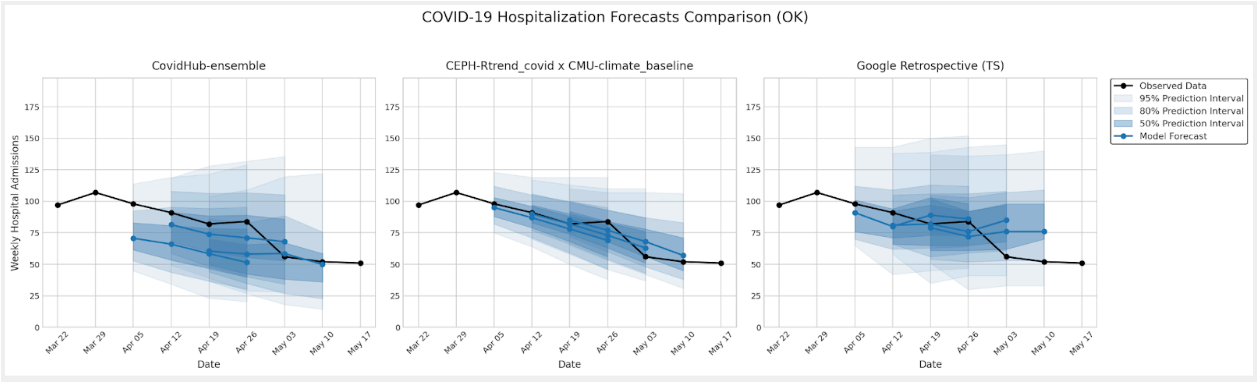
COVID-19 Hospitalization Forecasts Comparison (OK)
CovidHub-ensemble
CEPH-Rtrend_covid x CMU-climate_baseline
Google Retrospective (TS)
Weekly Hospital Admissions
Date
Observed Data
95% Prediction Interval
80% Prediction Interval
50% Prediction Interval
Model Forecast

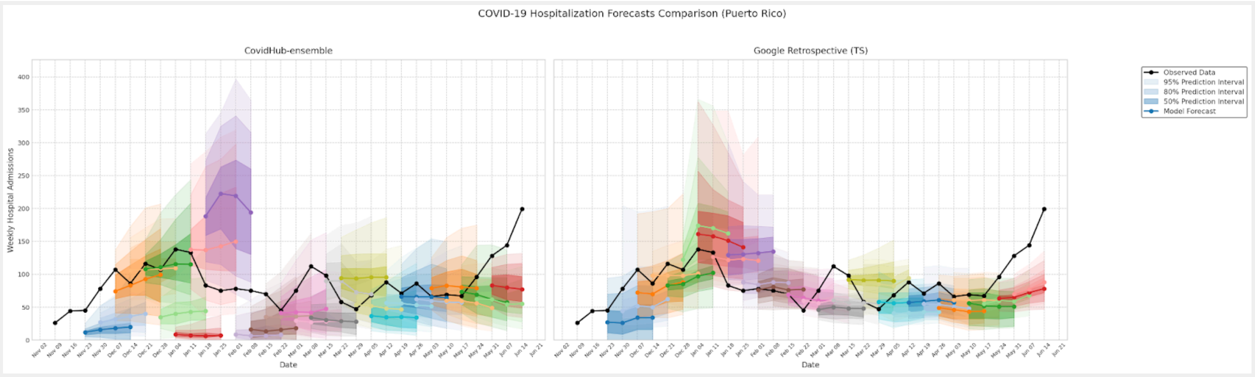
COVID-19 Hospitalization Forecasts Comparison (Puerto Rico)
CovidHub-ensemble
Google Retrospective (TS)
Weekly Hospital Admissions
Date
Observed Data
95% Prediction Interval
80% Prediction Interval
50% Prediction Interval
Model Forecast

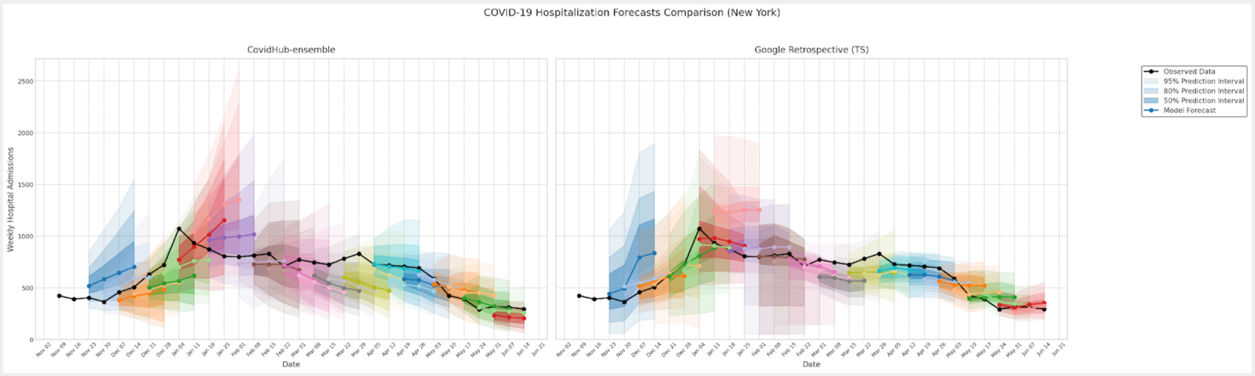
COVID-19 Hospitalization Forecasts Comparison (New York)
CovidHub-ensemble
Google Retrospective (TS)
Weekly Hospital Admissions
Date
Observed Data
95% Prediction Interval
80% Prediction Interval
50% Prediction Interval
Model Forecast

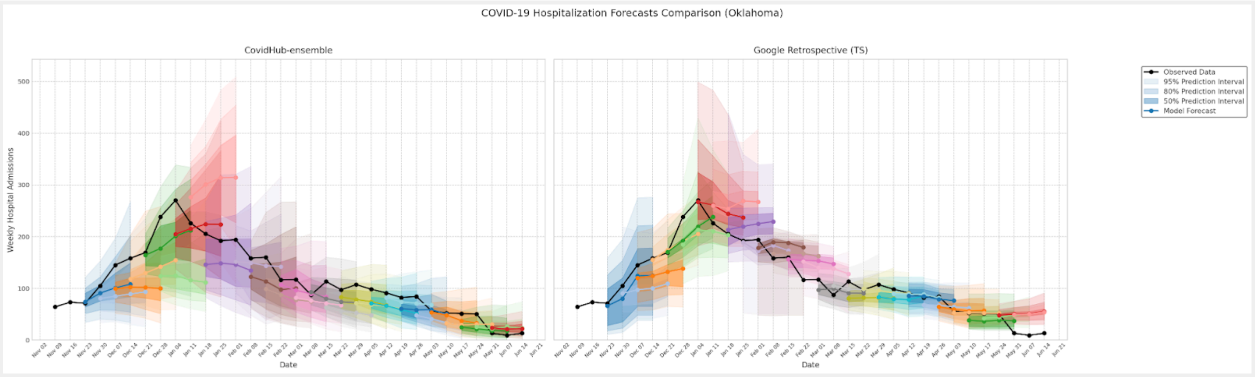
COVID-19 Hospitalization Forecasts Comparison (Oklahoma)
CovidHub-ensemble
Google Retrospective (TS)
Weekly Hospital Admissions
Date
Observed Data
95% Prediction Interval
80% Prediction Interval
50% Prediction Interval
Model Forecast

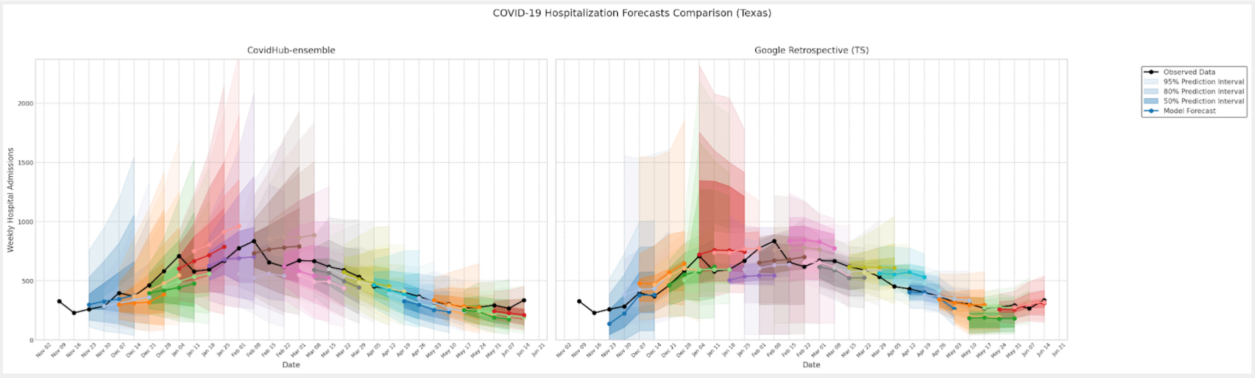
COVID-19 Hospitalization Forecasts Comparison (Texas)
CovidHub-ensemble
Google Retrospective (TS)
Weekly Hospital Admissions
Date
Observed Data
95% Prediction Interval
80% Prediction Interval
50% Prediction Interval
Model Forecast

2. Please clarify whether the proposed approach in the Covid domain is to run TS every few weeks to get new code or to discover a single piece of code that will generalize across temporal and geographic regimes.

   RESPONSE: In the retrospective study in this paper, we ran TS every two weeks to get new code. Following the suggestion of the referee, we have now included a supplemental table detailing the splits.

   It is worth noting that we are currently carrying out a follow up study over the entire 2025-6 season for flu, covid and RSV, and in this case we have used an alternative approach of generalizing a single piece of code across temporal and geographic regimes (for each disease.)

3. There are eight root nodes instead of one in the search tree. Was the root node left out? Or did TS start with the eight baseline solutions here?

   RESPONSE: Thank you for noticing this. The root node is left out. We will update this in the comments on the github repo.

4. “Models that perform worse than CovidHub-baseline are not shown.” Please clarify whether this includes solutions produced by TS, or just other models uploaded to CovidHub.

   RESPONSE: This includes both solutions produced by TS and other models uploaded to CovidHub. There are 9 models from TS and 3 from other submissions that perform worse than the baseline.

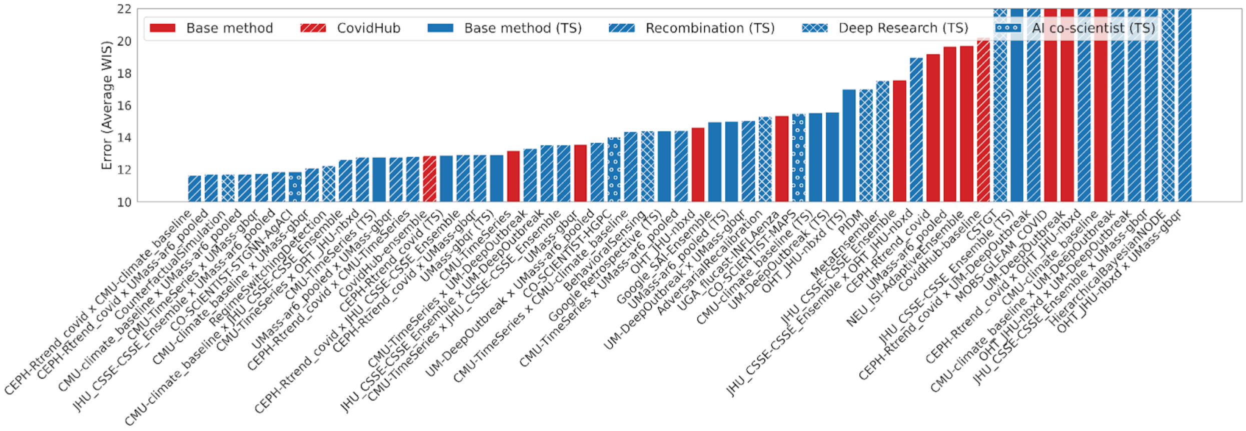

5.

   a. Please clarify what the 3-week evaluation period is in Figure 1e. Is this after the 14 search weeks in Figure 1a? Does that help explain why CovidHub-ensemble outperformed Google Retrospective here?

   RESPONSE: Across the 14 distinct models, each utilized a six-week rolling search window as a validation set followed by an evaluation over the subsequent

three weeks. The 14 rolling six-week validation sets covered the entire 2024-2025 COVID season. Google Retrospective results in Figure 1e specifically highlights the performance of a single model out of the 14 models where the search window immediately preceded this three-week assessment (2025-04-05, 2025-04-12, 2025-04-19). The selection of this particular evaluation period was made blindly; consequently, the Google Retrospective results happened to coincide with a period of relatively poor performance.

b. Was there evidence in validation that the best TS models in this test period (e.g., CEPH-Rtrend_covid x CMU-climate_baseline) were going to be the best?

   RESPONSE: Yes, there was evidence in the validation set. We show WIS scores of different models on the validation set in the figure below -- this covers the dates 2025-02-22, 2025-03-01, 2025-03-08, 2025-03-15, 2025-03-22, 2025-03-29. The performance on the validation dates matches the general trends observed in the test set. For instance, the best-performing model on the held-out test set, CEPH-Rtrend_covid x CMU-climate_baseline, was the second-best model on the validation set. While the precise ordering of models was not identical between validation and test sets, the results clearly suggested that this model would be among the top performers.

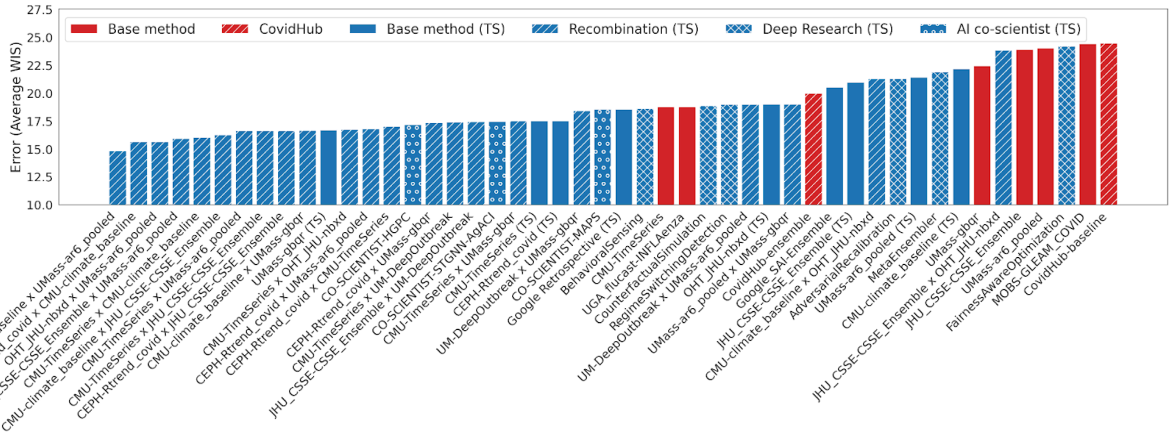

c. A Supplementary Table could be added that details the dates, time horizons, and locations used. More generally, since so many different system configurations are tried, it would help to more clearly delineate which scores correspond to validation and which to testing.

   RESPONSE. Thank you for this suggestion. We have included a supplemental table with this information below. The table is appended below for visibility.

Supplementary Table : Configuration of COVID Forecasting Data Splits

	Validation Reference Dates	Test Reference Dates	Horizons	Locations	Metric
Google Retrospective 1	2024-10-05, 2024-10-12, 2024-10-19, 2024-10-26, 2024-11-02, 2024-11-09	2024-11-16, 2024-11-23, 2024-11-30	1, 2, 3, 4 weeks	52 US jurisdictions	WIS
Google Retrospective 2	2024-10-19, 2024-10-26, 2024-11-02, 2024-11-09, 2024-11-16, 2024-11-23	2024-11-30, 2024-12-07, 2024-12-14	1, 2, 3, 4 weeks	52 US jurisdictions	WIS
Google Retrospective 3	2024-11-02, 2024-11-09, 2024-11-16, 2024-11-23, 2024-11-30, 2024-12-07	2024-12-14, 2024-12-21, 2024-12-28	1, 2, 3, 4 weeks	52 US jurisdictions	WIS
Google Retrospective 4	2024-11-16, 2024-11-23, 2024-11-30, 2024-12-07, 2024-12-14, 2024-12-21	2024-12-28, 2025-01-04, 2025-01-11	1, 2, 3, 4 weeks	52 US jurisdictions	WIS
Google Retrospective 5	2024-11-30, 2024-12-07, 2024-12-14, 2024-12-21, 2024-12-28, 2025-01-04	2025-01-11, 2025-01-18, 2025-01-25	1, 2, 3, 4 weeks	52 US jurisdictions	WIS
Google Retrospective 6	2024-12-14, 2024-12-21, 2024-12-28, 2025-01-04, 2025-01-11, 2025-01-18	2025-01-25, 2025-02-01, 2025-02-08	1, 2, 3, 4 weeks	52 US jurisdictions	WIS
Google Retrospective 7	2024-12-28, 2025-01-04, 2025-01-11, 2025-01-18, 2025-01-25, 2025-02-01	2025-02-08, 2025-02-15, 2025-02-22	1, 2, 3, 4 weeks	52 US jurisdictions	WIS
Google Retrospective 8	2025-01-11, 2025-01-18, 2025-01-25, 2025-02-01, 2025-02-08, 2025-02-15	2025-02-22, 2025-03-01, 2025-03-08	1, 2, 3, 4 weeks	52 US jurisdictions	WIS
Google Retrospective 9	2025-01-25, 2025-02-01, 2025-02-08, 2025-02-15, 2025-02-22, 2025-03-01	2025-03-08, 2025-03-15, 2025-03-22	1, 2, 3, 4 weeks	52 US jurisdictions	WIS
Google Retrospective 10	2025-02-08, 2025-02-15, 2025-02-22, 2025-03-01, 2025-03-08, 2025-03-15	2025-03-22, 2025-03-29, 2025-04-05	1, 2, 3, 4 weeks	52 US jurisdictions	WIS
Google Retrospective 11	2025-02-22, 2025-03-01, 2025-03-08, 2025-03-15, 2025-03-22, 2025-03-29	2025-04-05, 2025-04-12, 2025-04-19	1, 2, 3, 4 weeks	52 US jurisdictions	WIS
Google Retrospective 12	2025-03-08, 2025-03-15, 2025-03-22, 2025-03-29, 2025-04-05, 2025-04-12	2025-04-19, 2025-04-26, 2025-05-03	1, 2, 3, 4 weeks	52 US jurisdictions	WIS
Google Retrospective 13	2025-03-22, 2025-03-29, 2025-04-05, 2025-04-12, 2025-04-19, 2025-04-26	2025-05-03, 2025-05-10, 2025-05-17	1, 2, 3, 4 weeks	52 US jurisdictions	WIS
Google Retrospective 14	2025-04-05, 2025-04-12, 2025-04-19, 2025-04-26, 2025-05-03, 2025-05-10	2025-05-17, 2025-05-21, 2025-05-28	1, 2, 3, 4 weeks	52 US jurisdictions	WIS
All Other modelling strategies with TS	2025-02-22, 2025-03-01, 2025-03-08, 2025-03-15, 2025-03-22, 2025-03-29	2025-04-05, 2025-04-12, 2025-04-19	1, 2, 3, 4 weeks	52 US jurisdictions	WIS

Comments on the methodology:

The paper presents a convincing study of the power of combining LLMs with search to write expert-level empirical software, but the particular TS approach used is undermotivated. As is, the main motivation appears to be that TS worked well for AlphaGo and related systems, so why not try something similar here. The comparison to Best of 1000 in the Kaggle Playground confirms the value over random sampling, but not over other non-trivial search methods. Even a small amount of additional articulation of the advantages of TS could go a long way in making the approach feel less arbitrary and more informed by prior work, for example, by clarifying the following points:

1. TS comes out of a now long lineage of methods that combine LLMs with search to optimize code, including FunSearch (as referenced in the Discussion), but originating with methods using LLMs as drop-in operators in genetic programming, i.e. ELM for mutation (https://arxiv.org/abs/2206.08896) and LMX for recombination (https://arxiv.org/abs/2302.12170). TS does not “generalize” FunSearch, as claimed in the Discussion, rather it is a different class of search algorithm with different properties. E.g., one key difference is that the experiments in this paper use orders-or-magnitude fewer evaluations than those in the FunSearch paper, suggesting one potential advantage of TS is its sample efficiency. On the other hand, the Introduction claims the power comes from the ability of TS to “exhaustively” search, which suggests that if one were to run TS indefinitely, all potentially optimal solutions would be sampled. So, perhaps the advantage of TS is that it is both sample-efficient (as shown empirically across diverse domains) and (in some sense) complete.

RESPONSE. Thank you for these comments. We are updating the references to include these two papers. We agree with you that our algorithm is more than a generalization of FunSearch and we updated the text accordingly. We also agree that it is more sample efficient than evolutionary algorithms (including FunSearch and also AlphaEvolve). However we are actively doing benchmarking of these algorithms and we are not ready to make a definitive statement here -- which is beyond the scope of the current paper.

2. Another claimed advantage is the ability to recombine existing expert ideas. The approach is clearly inspired by evolutionary methods, but this inspiration is not articulated. The closest in spirit seems to be RHEA (https://arxiv.org/abs/2411.00156), where the comparative advantage of TS is that it recombines expert ideas in conceptual space instead of distilled model space.

RESPONSE. Thank you for this comment. We are including this reference in the discussion.

3. AIDE is included in Figure 1b but not mentioned in the text. Since this is the only quantitative comparison to prior approaches, it would be helpful to articulate features of TS might make it better than AIDE.

RESPONSE. Thank you. We have updated the text to read

TS also outperforms AIDE, owing to its ability to maintain a diverse tree of candidates, allowing it to backtrack when a specific line of code mutation plateaus.

4. Since many readers will have used LLMs to help write software, it would be helpful to somehow compare TS to the most common approach used: Asking the LLM for a solution and then iteratively asking the LLM to improve it. It might be possible to do this comparison without running additional experiments, but instead by analyzing the existing search trees, e.g., what would have happened if we had always expanded the most recent node? What would have happened if we had always expanded the best node so far? Presumably, both of these approaches would lead to much earlier stagnation. This gets back to the "completeness" of TS suggested in (1): It provides an elegant and practical way to get unstuck.

RESPONSE. This is a good point. In our view, the best way to think about this is as a comparison with the best of N. LLMs operate at finite temperature and are stochastic. Therefore if you ask the same question many times you get a distribution of answers. Some are better than others. Ordinarily one cannot read them all -- but by scoring the answers, we can rank them. What TS is doing is to use good answers to update the prompt to generate even better answers. By doing this at scale we are able to squeeze much more out of the model than is possible using LLMs the way they are commonly used.

Minor Comments:

1. How was the c_puct parameter tuned? A Supplementary Table with the c_puct used for each experiment would make the methodology more tangible and concrete. Ideally, the caption would include a description of how the values were tuned.

RESPONSE. We spent some time optimizing this parameter based on the Kaggle benchmark, but settled on c_puct=1, which we used throughout. This is now updated in the methods section. Thanks for flagging this.

2. For replicability, it would be helpful to include a Supplementary Table listing the Kaggle competitions included in the Kaggle Playground experiments.

RESPONSE. This is a good suggestion. We have included such a table as a new Supplementary Table 1.

3. Clarify use of TS in the following: "In total, 6/11 base methods..." -> "After TS was applied to each, 6/11 base methods…"

RESPONSE. Thank you for pointing out this ambiguity. We have updated the text exactly as suggested to improve clarity

4. "We allowed library to have" -> "We allowed the library to have"

RESPONSE. Fixed, thank you.

5. I like that the hybrid prompt asks for something “TRULY WONDERFUL”.

RESPONSE. ;-).

Referee #7 (Remarks on code availability):

The code produced by the system is provided for several experimental runs. The interface for exploring this code is useful, particularly in how it shows the diff from parent to child. Evaluation harnesses for running the AI-written code are not provided, but presumably would be not-too-difficult to replicate, since these are standard benchmarks. The code for tree search is not provided, but the algorithm is clear enough to reproduce from the pseudocode.

RESPONSE. Thank you. We are open sourcing a reference implementation of the code with the publication of this manuscript, but agree this is readily replicable from the pseudocode.

April 30, 2026

Dear Yann,

Thank you again for the very helpful referee reports on our paper, *An AI system to help scientists write expert-level empirical software*, and also for the discussion about the paper earlier this week.

We are very grateful for your help. As per our discussion, I've responded to the reports of the referees and updated the manuscript accordingly.

Additionally we discussed the comparison of Best of N vs Tree Search and other methods. Figure 1 of the manuscript compares Best of N vs Tree search for the Kaggle Benchmark that we used to develop this method, and also compares to AIDE -- which is another search method that was SOTA when we submitted the paper. As we discussed, I've now augmented this to include a comparison of Best of N and Tree search on the two main examples in the paper: an epidemiological forecasting benchmark, and the single cell batch integration benchmark. We are putting this in the methods section, as an example table after we introduce the methods. The comparison right now includes this for Gemini 2.5 Flash -- the model that we used on all analyses of the paper, and Gemini 3.1 Pro -- which is close to the current state of the art. We discussed also doing this comparison for other models (GPT5, Claude Opus). We are working on getting these numbers and will update the table before publication. Given the urgency of getting this paper done, I thought it would be better to send this back now so we can start to work on next steps.

I am also going to start the process of pushing the code that is in the code ocean capsule to the google research github repo (https://github.com/google-research/score) that hosts the tree visualizations and underlying codes. I hope to get that done next week so there is plenty of time for you to give feedback.

Finally I wanted to flag that Google finally decided on a name for this approach to iterative coding problems -- Empirical Research Assistance or ERA for short. This is the name that will be made public and so to align the paper with the name, I've updated this throughout.

Thanks so much -- I really appreciate your help with this.

Michael

Referees' comments:

Referee #1 (Remarks to the Author):

We thank the authors for their detailed rebuttal and the valuable additions to the manuscript. The inclusion of the pseudo-code and the new section on designing scoring metrics bring much-needed clarity to the optimization mechanics and the practical application of the framework. However, the overarching claim that d" still receives only limited evidence. While we appreciate the updated figure caption and the references to recent preprints, citing non-peer-reviewed work is not quite sufficient to validate the broad claims made in this manuscript.

Thank you for this comment. We certainly do not want to overclaim. We have gone through the manuscript carefully and softened the broad claims throughout. These changes include the following:

1. Introduction: " This **superhuman** performance arises because" changed to "This **expert-level** performance arises because of the ability..."
2. Discussion: "Instead of specializing in one domain, our system demonstrates a general **problem-solving capability**, achieving **expert-exceeding** performance on public leaderboards..." changed to "Instead of specializing in one domain, our system demonstrates a general **capability for empirical software optimization**, achieving **expert-level** performance on public leaderboards..."
3. Discussion: "**revolutionary** acceleration." changed to "**significant** acceleration"

Regarding the evaluation on the GIFT-Eval benchmark, we understand that the research was conducted prior to the release of several newer baselines. However, retaining the claim in the abstract that the method produces "state-of-the-art" software for time series forecasting is highly problematic given the current research landscape. Because the evaluation relies on an older snapshot and omits standard probabilistic metrics like the Continuous Ranked Probability Score (CRPS), the SOTA claim cannot be comfortably maintained. We strongly recommend either removing this specific SOTA claim or updating the evaluation to reflect current standards and baselines.

Thank you for this comment. We agree and have eliminated the "state of the art" claim in the abstract. We now state

Our method also produced state-of-the-art software for geospatial analysis, neural activity prediction in zebrafish, and numerical solution of integrals ***and a novel rule-based construction for time series forecasting***

While we agree with the referee that GIFT-Eval benchmark has progressed significantly since our snapshot. We remark though that performance is difficult to tease apart: in Fall 2025, GIFT-Eval changed their train and test sets (to resolve data leakage issues) and updated the leaderboard metrics computation. Official leaderboard entries from before this change are now

flagged, making it difficult to definitively tell if our models have dropped relative to newer architectures, or if the shift is simply because we evaluated on an older version of the data splits. (We previously detailed this shift in the methods section.)

Nonetheless, it remains the case that our solution to this problem is quite different than any current leading entry -- **we do not use foundation models at all,** and instead ask the tree search algorithm to find models that work using standard libraries. The per dataset model has extremely good performance and might still be SOTA. Even more interesting is the unified model, which builds a single model *with an adaptive configuration system of only 8 model types*. The system derives **rules** to choose which model to use given a dataset. The unified model had quite a respectable score before the dataset changes. The construction is a completely different paradigm than foundation models in that the pretraining phase is replaced with the adaptive configuration, which is much lighter weight and simpler to understand.

We considered moving the section to the supplemental information to respond to this critique -- but on thinking it over we think this example does illustrate one of the super-powers of tree search--the ability to find a rule based model that is competitive even against SOTA ML models. To emphasize these points, we wrote in the text

The resulting \texttt{unified solution} was highly competitive on the May 2025 leaderboard (\suptab{tab:leaderboard_snapshot_mase_full}), \textcolor{blue}{and remains one of the very few models on the leaderboard that is {\bf not} a foundation model; our model achieved this performance searching for rules to use variants of basic ML libraries.}

We have also made sure that nowhere in the text when we refer to the GIFT-Eval results do we use the phrase "state of the art". For example the revised abstract is quoted above.

While the proposed method represents a genuinely strong and innovative approach to empirical software optimization, the persistence of these fundamental issues prevents us from recommending the manuscript in its current form. The disconnect between the empirical evidence and the conceptual framing of "solving science," combined with the unsupported SOTA claims in the abstract, remain critical barriers. Consequently, these unresolved concerns lead us to decline the current submission.

Thank you for these comments. We hope that we have now adequately addressed your concerns.

Referee #1 (Remarks on code availability):

The results are not reproducible, as non-publicly-available imports are performed, and usage instructions and examples are missing. The submitted code is also very short and the

comments state that it is a "Generic implementation"; I therefore wonder how it relates to the code actually used to obtain the experimental results.

We did submit a Code Ocean Capsule with code and examples.

Referee #2 (Remarks to the Author):

I co-reviewed this manuscript with one of the reviewers who provided the listed reports.

Referee #4 (Remarks to the Author):

I co-reviewed this manuscript with one of the reviewers who provided the listed reports.

Referee #4 (Remarks on code availability):

There was a basic example and a README file. The code was easy to run and understand for the basic example. There was one minor issue for saving the output. Could not see `futz_progress.json` file initial, but then I edited line 252 in playground_s3e1.py to `/results`
A general problem is that the use of proprietary LLMs make it difficult to claim reproducibility without access to all the prompts and settings used for LLLs.

Referee #7 (Remarks to the Author):

Comments on requested updates:

Thank you to the authors for providing the requested additional details and updates. These go a long way in making the paper clearer and more concrete.

The extended plot including the 9 TS models and 3 from other submissions that were not better than the baseline would be great to include as a Supplemental Figure, as would the validation performance plot corresponding to the test performance plot in Figure 3e. Both are useful for concluding the results are meaningful.

Thank you for this comment. We are now including both of these plots as supplemental figures to the manuscript. We added the sentence to the manuscript

An extended performance plot including the 9 TS models and 3 other submissions that did not outperform the baseline, alongside their corresponding validation performance, is provided in Supplementary Figures \supfig{fig:covid_extended} and \supfig{fig:covid_validation}

The plots visualizing the actual predictions from different models are especially helpful in making the contribution concrete. However, since there are only a handful of prediction points on each curve, it is not immediately obvious to the reader why certain curves are better than others, so it would be very helpful to include in the caption of the figures a qualitative description of why the predictions from TS are better than the Ensemble, e.g., the middle column predictions are apparently linear, but are a very effective linear predictor, while the Retrospective predictions are relatively flat but with a small dip in the middle, and the clearest advantage of the TS methods are the narrower confidence bands that still usually contain the ground truth. This is the main behavior I see; I may have missed other qualitative advantages of the TS predictions.

We have included 4 panels of the figure provided in response to the referee as a supplemental figure. This figure has a caption that answers the referee's question

Caption Observed ground truth data (black line) plotted against predictive models across four major jurisdictions (CA, DC, FL, and NY) for the reference dates 2025-04-05, 2025-04-12, and 2025-04-19. These dates are used as the held out test set in the analysis in the paper. Columns represent the CovidHub-ensemble (left), the best-performing tree-search (TS) discovered recombination model (CEPH-Rtrend_covid x CMU-climate_baseline, middle), and the Google Retrospective TS model (right). The average WIS for this period are **11.63** for the top recombination model, **12.85** for the CovidHub-ensemble, and **14.39** for the Google Retrospective model. The TS methods generate significantly narrower confidence bands—indicating higher predictive precision—while consistently containing the ground truth data.

Thank you for mentioning AIDE in the main text. Please provide a citation there and mention what it is, e.g., "… AIDE^cite, a prior LLM-based tree-search method,..."

Thank you for this comment. We have now added this:

We change "TS substantially beats a single LLM call and best of 1000 LLM calls." to "TS substantially beats a single LLM call, the best of 1000 LLM calls, and AIDE [CITATION], a prior LLM-based tree-search method. TS outperforms AIDE owing to its ability to maintain a diverse tree of candidates, allowing it to backtrack when a specific line of code mutation plateaus."

The author response notes that RHEA will be mentioned in the discussion, but it is not in the revised version of the paper.

Thank you for noting this. We have added this to the discussion in teh section on combining LLMs and Search

"Furthermore, our approach to recombining expert ideas is conceptually related to evolutionary methods such as RHEA [CITATION], with the comparative advantage that our tree search recombines expert ideas directly in conceptual space via LLM prompts rather than solely in distilled model space."

Comments on framing:

The main consensus across reviewers seems to be that the paper offers valuable contributions, but the framing is not well-aligned with the contributions. The authors appear to agree with the framing concerns and that the framing can be clarified, but the paper text has not yet been updated to match.

Would the authors be willing to update the framing? This would be especially meaningful in the abstract and introduction.

For example, based on the reviews and author responses, an updated framing could be something like:

1. Writing scientific software is hard and time-consuming.
2. Recently, LLMs have shown the ability to improve code whose quality is “scorable”.
3. Inspired by this ability and the success of AlphaGo/AlphaZero, this paper introduces a general system that combines LLMs with tree search to write and improve scientific software.
4. The system makes meaningful improvements to scientific software across a diverse range of important scientific problems, including…
5. The system is able to effectively integrate expert ideas, and the validity and value of the improvements in each domain have each been verified by domain experts.
6. The results constitute the first demonstration of a system with the capacity to improve high-quality scientific software in general, thus representing a significant step in accelerating scientific progress.

It doesn’t need to be exactly this, but a framing like this makes it clear that, as the authors note, the main contribution is not algorithmic, but rather about presenting a broad convincing study whose value is supported by expert scientists from diverse fields.

We appreciate the referee's comments. To ensure that we are following the structure outlined by the referee, we mapped the referee's points to the sentences in the revised abstract. In the following we colored the referee's steps as green and the sentences in the abstract as purple.

✅ 1. Writing scientific software is hard and time-consuming. --> "The cycle of scientific discovery is frequently bottlenecked by the slow, manual creation of software to support computational experiments."
✅ 2. Recently, LLMs have shown the ability to improve code whose quality is “scorable”. --> "To address this, we present an AI system that creates expert-level scientific software whose goal is to maximize a quality metric."
✅ 3. Inspired by this ability and the success of AlphaGo/AlphaZero, this paper introduces a general system that combines LLMs with tree search to write and improve scientific software. --> "The system uses a Large Language Model (LLM) and Tree Search (TS) to systematically improve the quality metric and intelligently navigate the large space of possible solutions."

✅ 4. The system makes meaningful improvements to scientific software across a diverse range of important scientific problems, including…-->"
"The effectiveness of tree search is demonstrated across a \textcolor{blue}{diverse} range of \textcolor{blue}{tasks}.
"
✅ 5. The system is able to effectively integrate expert ideas, and the validity and value of the improvements in each domain have each been verified by domain experts. --> The system achieves expert-level results when it explores and integrates complex research ideas from external sources.
✅ 6. The results constitute the first demonstration of a system with the capacity to improve high-quality scientific software in general, thus representing a significant step in accelerating scientific progress. --> "By devising and implementing novel solutions to diverse tasks, the system represents a significant step towards accelerating scientific progress."

The steps match perfectly. We agree that this is a very useful framing for the paper.

Referee #7 (Remarks on code availability):

The code provided looks sufficient for implementing the algorithm and applying it to new applications. I did not try running it. Ideally, the code would also come with complete instructions for how to apply it to one or more of the applications in the paper, including complete prompts, so that future researchers can confirm their experiments are set up correctly.

Referee #8 (Remarks to the Author):

I co-reviewed this manuscript with one of the reviewers who provided the listed reports.
